# Patterns, trends and methodological associations in the measurement and valuation of childhood health utilities

**DOI:** 10.1007/s11136-019-02121-z

**Published:** 2019-02-19

**Authors:** Joseph Kwon, Sung Wook Kim, Wendy J. Ungar, Kate Tsiplova, Jason Madan, Stavros Petrou

**Affiliations:** 1 School of Health and Related Research, University of Sheffield, Sheffield, UK; 2 Warwick Clinical Trials Unit, Warwick Medical School, University of Warwick, Gibbet Hill Road, Coventry, CV4 7AL UK; 3 Program of Child Health Evaluative Sciences, The Hospital for Sick Children Research Institute, Toronto, Canada; 4 Institute of Health Policy, Management and Evaluation, University of Toronto, Toronto, Canada

**Keywords:** Systematic review, PRISMA, Childhood health states, Health utility, Economic evaluation, Cost–utility analysis

## Abstract

**Purpose:**

To systematically assess patterns and temporal changes in the measurement and valuation of childhood health utilities and associations between methodological factors.

**Methods:**

Studies reporting childhood health utilities using direct or indirect valuation methods, published by June 2017, were identified through PubMed, Embase, Web of Science, PsycINFO, EconLit, CINAHL, Cochrane Library and PEDE. The following were explored: patterns in tariff application; linear trends in numbers of studies/samples and paediatric cost–utility analyses (CUAs) and associations between them; changes in proportions of studies/samples within characteristic-based categories over pre-specified periods; impact of National Institute for Health and Care Excellence (NICE) guidance on primary UK research and associations between valuation method, age and methodological factors.

**Results:**

335 studies with 3974 samples covering all ICD-10 chapters, 23 valuation methods, 12 respondent types and 42 countries were identified by systematic review. 34.0% of samples using indirect methods compatible with childhood applied childhood-derived tariffs. There was no association between numbers of studies/samples and numbers of CUAs. Compared to 1990–2008, 2009–June 2017 saw a significant fall in the proportion of studies using case series; significant compositional changes across ICD-10 chapters and significantly higher sample proportions using childhood-specific and adult-specific indirect valuation methods, and based on pre-adolescents, self-assessment, self-administration and experienced health states. NICE guidance was weakly effective in promoting reference methods. Associations between valuation method, age and methodological factors were significant.

**Conclusion:**

1990–2017 witnessed significant changes in primary research on childhood health utilities. Health technology assessment agencies should note the equivocal effect of methodological guidance on primary research.

**Electronic supplementary material:**

The online version of this article (10.1007/s11136-019-02121-z) contains supplementary material, which is available to authorized users.

## Introduction

Sustained health budget constraints necessitate the comparison of alternative programmes and interventions in terms of costs and consequences [1]. Cost–utility analysis (CUA) remains the preferred form of economic evaluation aimed at informing the allocation of finite resources for decision-making agencies around the world [2–6]. In CUA, the quality-adjusted life-year (QALY) is the preferred measure of health outcome where the QALY combines preference-based health-related quality of life (HRQoL) outcomes, or health utility values, associated with health states, with the length of time in those states [7]. The generic nature of health utilities (and thus QALYs) allows comparison of healthcare interventions across disparate health conditions and populations. Health utilities are indexed on a cardinal scale where 0 represents death and 1 represents perfect health [1].

Valuation methods for utility assessment can be divided into two broad categories: direct and indirect. Direct methods combine the valuation and measurement process into a single step, and include the standard gamble (SG) technique and the time trade-off (TTO) approach [1]. The visual analogue scale (VAS) is another direct valuation method, although considered by some economists not to be a health utility measurement approach since the valuation procedure is not choice based and does not involve decision making under uncertainty [8]. Indirect valuation methods use multi-attribute utility instruments (MAUIs) such as the EQ-5D [9], Health Utilities Index (HUI) [10], SF-6D [11], Quality of Well-Being Scale (QWB) [12] and Assessment of Quality of Life (AQoL or AQoL-5D) [13]. MAUIs ask respondents to describe their health state using a health status classification system containing several dimensions, each with multiple levels. Algorithms, or tariff sets, elicited from representative populations using direct valuation methods such as the TTO, are then applied to convert responses into health utility values.

Unique methodological challenges arise when utility assessments are conducted for childhood (age < 18 years) health conditions or states [14, 15]. For example, infants (age < 2 years), as well as pre-adolescents (age < 12 years) and adolescents (age ≥ 12 and < 18 years) with developmental delays or cognitive deficits arising from neurodevelopmental disorders, lack comprehension to complete direct or indirect valuation methods, thus requiring proxy assessment. Moreover, bio-psychosocial development during childhood means that dimensions relevant to HRQoL change rapidly by age [16]. This means that classification systems embedded into adult-specific MAUIs (targeted at those aged ≥ 18 years), such as the EQ-5D and SF-6D, may not incorporate health dimensions relevant to developmental stages through childhood. MAUIs with childhood-specific classification systems have been developed to meet this challenge, including the EQ-5D-Y (Youth) [17], 16-Dimensional Health-Related Measure (16D) [18], 17-Dimensional Health-Related Measure (17D) [19], AQoL-6D [20] and Child Health Utility 9-Dimensions (CHU9D) [21]. Moreover, several MAUIs, such as the HUI2, HUI3 and QWB, have classification systems compatible with both adult and childhood health states. Many measures with classification systems designed specifically for or compatible with childhood, such as the EQ-5D-Y, HUI3 and QWB, rely on tariffs derived from adult populations. Thus, another methodological concern relates to potential differences between children and adults in how they value health states contained within MAUIs [22].

There has been increasing recognition of these methodological challenges in international health technology assessment (HTA) guidelines. The 2013 National Institute for Health and Care Excellence (NICE) methods guidance recognised that its preferred measure, the EQ-5D, lacked a classification system designed for use in children and recommended use of the EQ-5D-Y for children aged 7–12 years, although no separate tariff set for this measure existed [2]. The 2016s US Panel on Cost-effectiveness in Health and Medicine acknowledged the challenges surrounding childhood utility measurement and discussed the relative advantages of alternative instruments, including HUI2/3, EQ-5D-Y and CHU9D, but without recommending a preferred approach [6]. Despite these developments, adult-specific health utilities are still frequently applied to childhood health states within economic evaluations. Montgomery and Kusel [23] reviewed all NICE health technology appraisals in England until June 2015 and identified 29 submissions directly related to paediatric health, only six of which applied childhood-specific utilities. It is generally unclear how developments of national HTA guidelines have influenced priorities and designs, including choice of valuation methods, in primary assessments of childhood health utilities.

In line with the preference of several HTA agencies, CUA is now the leading analytic approach for economic evaluation of health interventions targeting children, with the number of CUAs overtaking that of cost-effectiveness analyses (CEAs) contained in the Paediatric Economic Database Evaluation (PEDE) in 2009 [24]. Although health utility data constitute vital inputs into CUAs, it is rare for analysts to estimate utility values using primary research methods unless the economic evaluation is conducted alongside a prospectively designed study with individual-level data [25]. Decision-analytic models typically contain several health states of interest that require sourcing of utility estimates from primary studies or systematic reviews. In such cases, the International Society for Pharmacoeconomics and Outcomes Research (ISPOR) guideline [26] recommends that analysts identify and extract health utilities from multiple sources in the published literature and synthesise if appropriate.

Catalogues and reviews of health utility values for childhood populations are available, but have largely focussed on a relatively small number of conditions, such as acute lymphoblastic leukaemia [27, 28], asthma [29], neurodisability [30] and childhood obesity [31], or on a single valuation source, such as EQ-5D [32], HUI3 [33] and SG [34]. The PEDE project is an alternative source of childhood health utility values and contains 2112 values from 857 CUAs in paediatric populations published between 1980 and 2016 [35]. However, only 8.5% (*n* = 73) of these CUAs conducted primary estimation of utility values [36]. Furthermore, published CUAs represent only one source of childhood health utility values.

This paper addresses three aims against a backdrop of rapid development of measurement approaches and methods guidance in this area. First, it systematically describes the patterns of primary studies and samples measuring childhood health utilities, by study design and sample characteristics, as well as patterns in the source of tariffs when MAUIs compatible with or specific to children are used. Second, linear trends in the numbers of studies and samples, their associations with the number of paediatric CUAs and changes in proportions of studies and samples before and after key transition timepoints are explored. Third, associations between valuation methods and sample age and other methodological factors are explored.

## Methods

### Systematic review

The systematic review followed PRISMA guidelines [37] and covered all studies published by 30th June 2017. The search strategy (Supplementary Material Table S1), inclusion and exclusion criteria and databases included—PubMed, Embase of OVID Medline, Web of Science, PsycINFO, Cochrane Library, CINAHL and EconLit—were based on the study by Kwon and colleagues [38], which covered a period up to 31 December 2015 and focused only on static descriptions of included studies and samples alongside a meta-regression of childhood utility values. The search strategy was developed and piloted prior to implementation and included an intersection of health utility, valuation method and childhood search terms. Non-English language articles were excluded. The PEDE database was also searched to identify CUAs published between 1980 and 2016 that incorporated primary estimation of health utilities.

Articles that met the inclusion criteria were English language primary studies reporting health utilities for childhood populations or for childhood conditions or descriptors using direct or indirect valuation methods. Two reviewers (JK and SWK) independently assessed titles and abstracts. Articles with two approvals proceeded to the next stage; those with one were referred to a third reviewer (SP) for arbitration. At the second stage, the same primary reviewers analysed full-text articles with disagreements referred to the third reviewer. Conference abstracts were included if they reported original health utilities. Studies reporting primary VAS values were included despite disagreement about their validity for QALY construction [39].

### Data extraction

From each study that met the inclusion criteria, the variables listed in Supplementary Material Table S2 were extracted using a proforma. These included bibliographic details, study design, study setting, valuation method, tariff applied to MAUIs (including source population and valuation method), respondent type, administration mode, sample target age(s), whether the health state was experienced or hypothetical, sample size and geographic setting. An International Classification of Diseases 10th revision (ICD-10) code was allocated to each sample according to the health condition studied.

### Descriptive analyses

The distribution of mean and median utility and VAS (rescaled to 0–1) scores extracted from included samples was calculated. The number of studies and samples by publication year was estimated together with the annual number of CUAs included in the PEDE database. Valuation methods applied to studies within each ICD-10 chapter were described. The numbers of samples by respondent type, administration mode, target age(s) of sample and geographical factors (including continent of origin and national income levels, with income classification taken from the World Bank [40]) were estimated. Study designs and valuation methods were grouped into key categories. For application of MAUIs that are compatible with or specific to childhood populations, the tariffs applied and sources of their values (age group and setting/nationality) and valuation method (e.g. VAS, SG, TTO) were described by MAUI.

### Statistical analyses

Linear regression tested for linear trends in numbers of PEDE-based CUAs, utility studies and utility samples by year of publication. Controlling for these annual trends, associations between number of PEDE-based CUAs and number of utility studies and samples were also estimated. We tested the hypothesis that there were significant changes to the composition of childhood utility data categorised by study- and sample-level characteristics, including study design, health condition, valuation method, age of target population, respondent type, administration mode and valuation of hypothetical health states. Tests of proportions, at the 95% confidence level, were used to assess whether the proportion in each category of study- and sample-level characteristic changed significantly before and after a pre-specified transition point of 2009, which was when CUAs became the prominent analytic approach for paediatric economic evaluations within the PEDE database [24]. In a separate analysis involving only UK studies and samples, 2013 was specified as a transition point when NICE identified EQ-5D-Y as its reference instrument for utility assessment in children aged 7–12 years, while reaffirming EQ-5D as its reference instrument for those aged 13 years and over [2]. The hypothesis was that the guidance had a significant effect on the design of UK-based primary studies marked by a greater proportion of samples using the EQ-5D-Y for pre-adolescent populations (age < 12 years) and smaller proportions using non-reference direct or indirect valuation methods. Finally, two-way Pearson’s Chi-square tests were used at the 95% confidence level to assess the associations between valuation method and sample age, respondent type, administration mode and valuation of hypothetical health states. The hypothesis tested was that there are significant associations between methodological factors selected by researchers.

## Results

### Systematic review

**Fig. 1:**
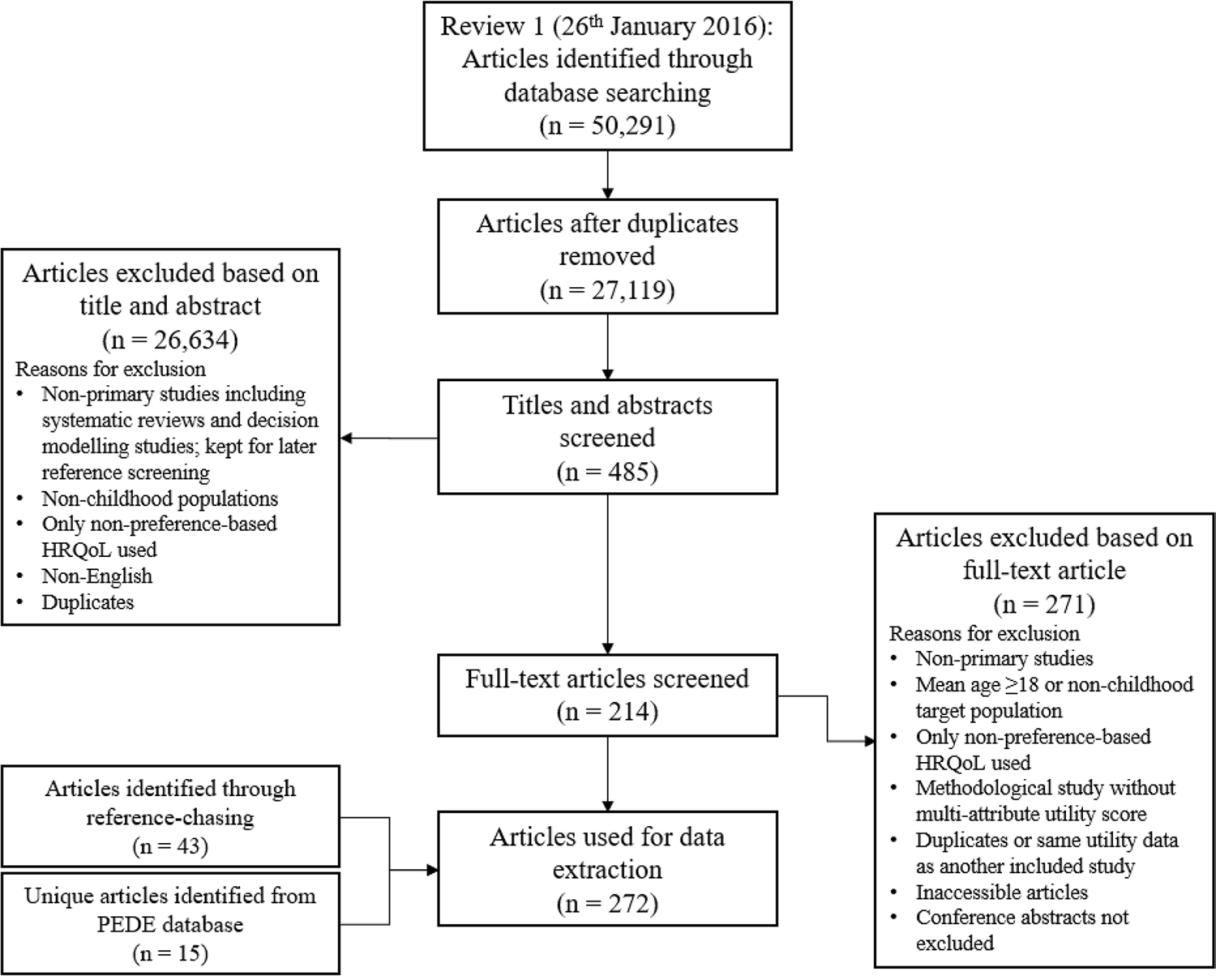
PRISMA flow diagram. *Note* PRISMA: Preferred reporting items for systematic reviews and meta-analyses. *HRQoL* Health-related quality of life. *PEDE* paediatric economic database evaluation

Figure 1 presents the PRISMA flow diagram for the systematic review. The main reasons for exclusion were secondary studies using decision-analytic models, previous systematic reviews (which were kept for manual searching), targeting of adult populations and focus on non-preference-based health outcome measures. A total of 274 articles were included for data extraction. Manual searching yielded 45 further articles, whilst a search of PEDE yielded a further 16 articles. In total, data were extracted from 335 articles, which are summarised by health condition, intervention type (where applicable), country of study population, valuation method, respondent type, administration mode and age of target population in Supplementary Material Table S3.

### Descriptive analyses

#### Distribution of sample means and medians

**Fig. 2:**
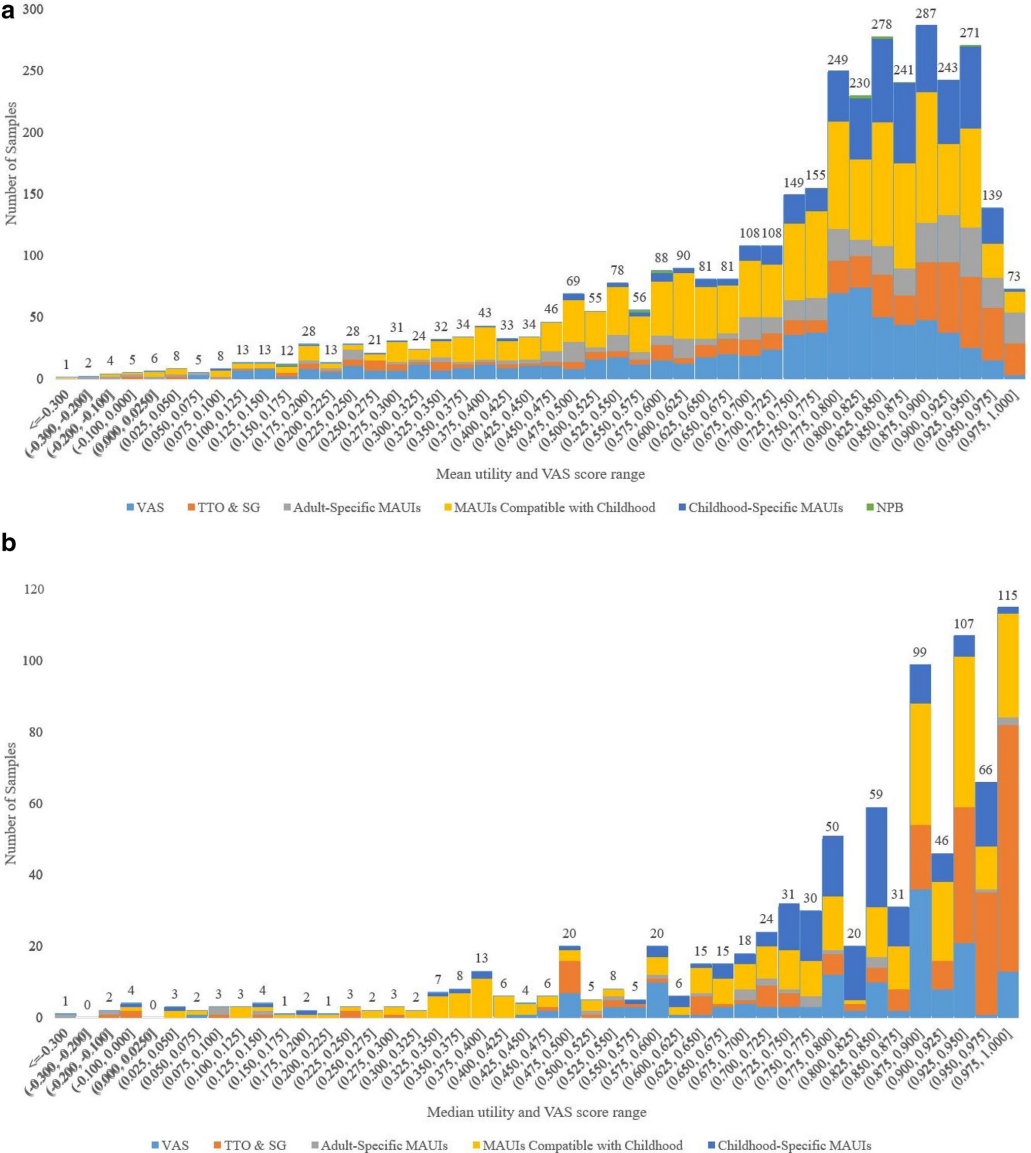
**a** Distribution of mean utility and VAS scores (*n* = 3573) by valuation method. *Note* VAS: visual analogue scale; TTO: time trade-off; SG: standard gamble; MAUI: multi-attribute utility instrument; NPB: utility mapped from non-preference-based instrument. **b** Distribution of median utility and VAS scores (*n* = 870) by valuation method. *NoteVAS* visual analogue scale, *TTO* time trade-off, *SG* standard gamble, *MAUI* multi-attribute utility instrument

Descriptive statistics for health utilities were extracted from 3974 samples contained within the 335 studies. The majority of studies (306 out of 335) contained two or more samples delineated by health condition/state, sociodemographic factors (e.g. gender, age) or methodological factors. Figure 2a depicts the frequency distribution of 3573 mean utility and VAS scores (excluding 191 samples, which only reported mean change in utility scores or regression coefficients) with each bar stratified by valuation method, while Fig. 2b does so for 870 median utility and VAS scores (some samples report both mean and median scores). The difference in distributional characteristics between mean and median scores is visible, with the negative skew much greater in median scores. Among samples reporting mean scores, 0.34% (12 out of 3573) demonstrated the ceiling effect, namely a mean score of 1. The corresponding proportion was 7.36% (64 out of 870) among samples reporting median scores. From visual inspection, there appears to be greater concentrations of trade-off-based direct valuation methods (TTO, SG and their variants) at the upper end of both mean and median utility score ranges. Trade-off-based direct valuation methods comprised 17.9% of all samples generating mean utility scores higher than 0.800, but 10.5% of all samples with mean scores ≤ 0.800. The corresponding proportions were 33.0% and 14.0% for all median utility scores higher than 0.800 and ≤ 0.800, respectively.

#### Distribution of samples by health condition and valuation method

Table 1 summarises the distribution of included samples by ICD-10 chapter. All ICD-10 chapters relevant to childhood health were covered by samples included in the systematic review. The ICD-10 mental and behavioural disorders chapter contained the highest number of samples (*n* = 698), followed by general childhood population health (*n* = 501) and cancer (*n* = 442). Across all ICD-10 chapters, 180 unique ICD-10 codes were used to label samples.

**Table 1:** Number of samples by ICD-10 chapter and the most frequently used valuation methods by ICD-10 chapter (% of samples in chapter)

*General health: 501* CHU9D (27.4) EQ-5D-Y VAS (24.4) EQ-5D-Y (10.6)	*Chapter 1 Infectious and parasitic diseases: 195* VAS (24.1) EQ-5D (15.9) TTO (15.4)	*Chapter 2 Cancer: 442* HUI2 (52.0) HUI3 (37.1) VAS (2.94)
*Chapter 3 Diseases of blood*: 85 SG (43.5) EQ-5D (24.7) VAS (12.9)	*Chapter 4 Endocrine, nutritional and metabolic disorders*: 411 SG (14.1) HUI3 (12.9) EQ-5D-Y VAS (12.4)	*Chapter 5 Mental and behavioural disorders*: 698 EQ-5D (20.2) HUI3 (18.9) EQ-5D VAS (16.6)
*Chapter 6 Nervous system disorders*: 114 HUI3 (43.0) SF-6D (9.65) EQ-5D (7.89)	*Chapter 7 Diseases of the eye*: 61 TTO (42.6) Chained Gamble (42.6) HUI3 (14.8)	*Chapter 8 Diseases of the ear*: 158 HUI3 (58.2) VAS (13.3) HUI2 (6.96)
*Chapter 9 Circulatory system disorders*: 16 VAS (31.3) HUI3 (31.3) HUI2 (18.8)	*Chapter 10 Respiratory system disorders*: 192 PAHOM (35.8) EQ-5D VAS (16.8) EQ-5D (12.1)	*Chapter 11 Digestive system disorders*: 60 16D/17D (20.0) EQ-5D-Y VAS (18.3) CHU9D (16.7)
*Chapter 12 Diseases of the skin*: 26 VAS (38.5) TTO (26.9) EQ-5D (15.4)	*Chapter 13 Musculoskeletal system disorders*: 114 EQ-5D (37.7) EQ-5D VAS (17.5) VAS (14.9)	*Chapter 14 Genitourinary system disorders*: 56 VAS (30.4) TTO (26.8) HUI3 (16.1)
*Chapter 16 Conditions originating in perinatal period*: 210 HUI3 (38.1) SG (33.3) HUI2 (15.7)	*Chapter 17 Congenital malformations*: 155 HUI3 (33.6) VAS (23.2) TTO (18.1)	*Chapter 19 Injury, poisoning and other consequences of external causes*: 297 QWB (40.7) EQ-5D (29.0) EQ-5D VAS (13.1)
*Chapter 21 Contact with health services*: 80 HUI3 (48.8) HUI2 (36.3) 15D/16D/17D (8.75)	*Combined chronic diseases*: 103 HUI3 (40.8) EQ-5D-Y VAS (23.3) EQ-5D VAS (12.6)	

No samples were found for ICD-10 chapter 15 for pregnancy, childbirth and puerperium; chapter 16 for symptoms, signs and abnormal clinical and laboratory findings, not elsewhere classified and chapter 20 for external causes of morbidity and mortality. These chapters were deemed not directly relevant to childhood health. The ‘general health’ category included samples measuring utility in general paediatric populations drawn from the general community or schools or in control groups of healthy children from observational and experimental studies. Chapter 21 classifies samples by contact with health services rather than disease type. These samples are drawn from studies delineated by interventions or programmes and a health condition is not specified. The ‘combined chronic diseases’ category included samples comprising children with diverse chronic conditions

Twenty-three unique valuation methods were identified. These were grouped into six key categories: (i) VAS—EQ-5D VAS (number of samples = 348; 8.8%), EQ-5D-Y VAS (*n* = 232; 5.8%) and stand-alone VAS (*n* = 252; 6.3%); (ii) trade-off-based direct valuation methods—TTO (*n* = 171; 4.3%), SG (*n* = 227; 5.7%), chained gamble and adjusted SG (*n* = 143; 3.6%); (iii) adult-specific MAUIs—EQ-5D (*n* = 424; 10.7%), SF-6D (*n* = 34; 0.9%), AQoL-5D (*n* = 16; 0.4%) and 15D (*n* = 2; 0.05%); (iv) MAUIs compatible with both childhood and adult populations—QWB (*n* = 224; 5.6%), HUI2 (*n* = 482; 12.1%), HUI3 (*n* = 822; 20.7%), modified HUI (10-dimension variant of HUI [41], HUI3 with ‘worst imaginable health’ as 0 instead of death [42]; *n* = 8; 0.2%) and ABC-UI (Aberrant Behaviour Checklist Utility Index) [43] (*n* = 1; 0.03%); (v) childhood-specific MAUIs—EQ-5D-Y (*n* = 108; 2.7%), CHU9D (*n* = 231; 5.8%), 16D (*n* = 73; 1.8%), 17D (*n* = 39; 1.0%), AQoL-6D (*n* = 50; 1.3%), PAHOM (Pediatric Asthma Health Outcome Measure) [44] (*n* = 69; 1.7%) and CH-6D (Child Health-6 Dimensions) [45] (*n* = 3; 0.08%);and (vi) mapping non-preference-based clinical measures to utility indices [46–50] (*n* = 15; 0.4%).

Table 1 describes the three most frequently used valuation methods by ICD-10 chapter. HUI2 or HUI3 was used by 89.1% of cancer samples, which is consistent with these measures originally being developed for paediatric cancer patients and survivors [51, 52]. Childhood-specific MAUIs represented the most frequently used valuation method for only 3 out of 20 chapters: CHU9D (27.4% for general health), PAHOM (35.8% for respiratory system disorders) and 16D/17D (20.0% for digestive system disorders). MAUIs compatible with childhood and adult populations were the most frequently used methods for eight chapters: HUI2 for chapter 2, HUI3 for Chaps. 6, 8, 16, 17 and combined chronic diseases and QWB for chapter 19.

#### Distribution of samples by methodological factors, age and geographical setting

Table 2 presents the number of study samples by respondent type, administration mode, target age of children and geographical setting. Thirty-seven percent of samples (*n* = 1498) used self-assessment by children. A total of 456 samples (11.5%) allowed children to generate responses together with proxies. The remainder of the samples relied on proxy assessment, most commonly by parents (*n* = 1091; 27.5%). Administration modes fell broadly into two categories: (i) self-administered surveys in school or clinical settings, by mail, online or by Delphi elicitation of clinicians [53]; and (ii) interview-administered surveys by face-to-face meeting or by telephone. Target age groups spanned the whole age spectrum of childhood. Six percent of samples contained infants (aged < 2 years), while some samples (*n* = 41) had a minimum age of 18 years even though they specified adolescents as the target group. A significant proportion of samples (14.4%) did not report any information on age. As for geographical setting, the skew towards high-income countries was clear (91.6%). The US produced the highest number of samples (*n* = 970; 24.4%) followed by Canada (*n* = 674; 17.0%) and the UK (*n* = 634; 16.0%).

**Table 2:** Number of samples by respondent type, mode of administration, age of children and geography (% of all samples)

Number of samples by respondent type					
*Self-assessment: 1498 (37.7)*					
Self-assessment by children: 1498					
*Proxy assessment—joint assessment by children and proxy*: 456 (11.5)					
Assessment by children and parents: 391		Assessment by children and caregivers: 65			
*Proxy assessment*: 2020 (50.8)					
Proxy assessment by parents: 1091		Proxy assessment by caregivers: 435		Proxy assessment by physicians: 256	
Proxy assessment by physicians and caregivers: 47		Proxy assessment by nurses: 76		Proxy assessment by the general public: 24	
Proxy assessment by parents from the general public: 77		Proxy assessment by adult patients: 7		Proxy assessment by parents, adult patients and the general public: 7	
Number of samples by administration mode					
*Self-administration by respondents*: 2464 (62.0)					
Non-postal survey: 1276		Postal survey: 864		Online survey: 319	
Delphi process: 5					
*Interview-administration*: 1478 (37.2)					
Face-to-face interview: 1198		Telephone interview: 265		Face-to-face or telephone: 15	
*Mode of administration not specified*: 32 (0.8)					
Number of samples by age of children in sample					
*Pre-adolescent children (age* < *12 years) in sample*: 2086 (52.5)					
Minimum age of 0: 238		Minimum age of 2: 247		Minimum age of 5: 560	
Minimum age of 8: 1041					
*No pre-adolescent children in sample*: 1315 (33.1)					
Minimum age of 12: 776		Minimum age of 15: 498		Minimum age of 18: 41	
Age unspecified: 573 (14.4)					
Number of samples by geography					
*High-income countries in Europe*^a^: 1522 (38.3)					
Austria: 12	Belgium: 5	Denmark: 10	Finland: 114	France: 4	Germany: 49
Hungary: 3	Italy: 24	Netherlands: 445	Norway: 2	Portugal: 2	Spain: 34
Sweden: 184	UK: 634				
*North America*: 1644 (41.4)					
Canada: 674	US: 970				
*Other high-income countries*: 473 (11.9)					
Australia:263	New Zealand: 14	Singapore: 44	South Korea: 11	Developed countries: 141	
*Central and South America*: 79 (2.0)					
Argentina: 2	Brazil: 5	Colombia: 22	Honduras: 8	Uruguay: 12	South America: 30
*Africa*: 89 (2.2)					
Kenya: 30	Sierra Leon: 4	South Africa: 47	Uganda: 4	Zimbabwe: 4	
*Other low- and middle-income countries*^a^: 156 (3.9)					
Bulgaria: 3	China: 36	Cuba: 16	India: 2	Iran: 14	Russia: 8
Thailand: 71	Turkey: 6				
*Other*: 11 (0.3)					
Developed and developing: 2	Not specified: 9				

^a^Low-, middle- and high-income country categories as defined by World Bank [40]

#### MAUIs and tariff application

Table 3 lists the tariffs that were used in applications of MAUIs compatible with or specific to childhood populations and their valuation populations and methods. All 16D and 17D samples that provided information applied tariffs derived from children or their proxies (Finnish schoolchildren aged 12–16 years using VAS [19] for 16D or their proxies (Finnish parents of children aged 8–11 years) using VAS [20] for 17D). For AQoL-6D, 72.0% of samples applied adolescent-derived tariffs [54], while 28.0% applied the general adult-derived tariff [55]. Adolescent-derived tariffs [56, 57] were used in 46.3% of CHU9D samples and general adult-derived tariffs [58] in 52.8%. Only a single EQ-5D-Y sample applied childhood-derived tariffs [59], while 78.7% of samples applied general adult-derived tariffs from 10 countries [60–67]. Overall, 54.3% of samples using childhood-specific MAUIs applied childhood-derived tariffs, 40.0% applied adult-derived tariffs and 5.7% gave no information on underpinning tariffs. For MAUIs compatible with both childhood and adult populations, 26.4% of samples applied childhood-derived tariffs, while 67.5% applied adult-derived tariffs. HUI2 offers both childhood- [68–70] and adult-derived [71] tariffs, while the HUI3, QWB and ABC-UI only offer adult-derived tariffs [72–74].

**Table 3:** Multi-attribute utility instruments (MAUIs) developed for children and tariff valuation population and method

MAUI	Target age range	Tariff valuation population and method	Number of samples (%)
Childhood-specific MAUIs			
16D	12–15	Finnish schoolchildren aged 12–16 (*n* = 213) using VAS [18]	68 (93.2)
		No information or reference on tariff	5 (6.8)
17D	8–11 (Proxy for < 8)	Finnish parents of children aged 8–11 (*n* = 115) using VAS [19]	39 (100.0)
AQoL-6D	Adolescents	1. Australian adolescents (*n* = 2790) using TTO [69]	36 (72.0)
		2. Australian general adult public (*n* = 411) using TTO [70]	14 (28.0)
CHU9D	7–17	1. Australian adolescents aged 11–17 (*n* = 590) using best-worst scale discrete-choice experiment [71]	91 (39.4)
		2. Australian adolescents aged 11–17 (*n* = 1982) using best-worst scale discrete-choice experiment [72]	16 (6.9)
		3. UK general adult public (*n* = 300) using SG [73]	122 (52.8)
		No information or reference on tariff	2 (0.9)
EQ-5D-Y	8–15 (Proxy for 4–7)	1. Canadian elementary schoolchildren aged 10–11 (*n* = 4485) using VAS [74]	1 (0.9)
		2. UK general adult public (*n* = 3395) using TTO [75]	25 (23.1)
		3. Dutch general adult public (*n* = 309) using TTO [76]	9 (8.3)
		4. Dutch general adult public (*n* = 303) using TTO [77]	5 (4.6)
		5. Australian general adult public (*n* = 417) using TTO [78]	14 (13.0)
		6. French general adult public (*n* = 452) using TTO [79]	3 (2.8)
		7. Swedish general adult public (*n* = 45,000) using TTO and VAS [80]	4 (3.7)
		8. General adult public from Finland, Germany, Netherlands, Spain, Sweden and UK (*n* = 6870) using VAS [81]	2 (1.9)
		9. US general adult public using TTO [82]	23 (21.3)
		No information or reference on tariff	22 (20.4)
PAHOM	7–13	1. Asthmatic children aged 6–12 (*n* = 261) using SG [83]	60 (87.0)
		2. US general adult public (*n* = 94) using VAS [44]	4 (5.8)
		3. US general adult public (*n* = 101) using SG [44]	4 (5.8)
		No information or reference on tariff	1 (1.4)
CH-6D	16–18	No information or reference on tariff	3 (100.0)
MAUIs compatible with childhood and adult populations			
HUI2	≥ 5 (Proxy for 5–8)	1. Canadian parents of schoolchildren (*n* = 194) using VAS and SG [84]	357 (74.1)
		2. Canadian parents of normal children (*n* = 300) using VAS and SG [85]	22 (4.6)
		3. Singaporean parents of paediatric cancer patients (*n* = 59) using VAS and SG [86]	10 (2.1)
		4. Singaporean general public parents of children (*n* = 194) using VAS and SG [86]	10 (2.1)
		5. UK general adult public (*n* = 201) using VAS and SG [87]	58 (12.0)
		No information or reference on tariff	25 (5.2)
HUI3	≥ 5 (Proxy for 5–8)	Canadian general adult public (*n* = 504) using VAS and SG [88]	753 (91.6)
		No information or reference on tariff	69 (8.4)
Modified HUI [39, 40]	N/A	1. Canadian parents of schoolchildren (*n* = 194) using VAS and SG [84]	7 (87.5)
		2. Canadian general adult public (*n* = 504) using VAS and SG [88]	1 (12.5)
QWB	N/A	US general public (*n* = 800) using VAS [89]	224 (100.0)
ABC-UI	N/A (Proxy completed)	UK general adult public (*n* = 349) using TTO [90]	1 (100.0)

### Statistical analyses

#### Linear trend in utility studies and samples and PEDE CUAs

**Fig. 3:**
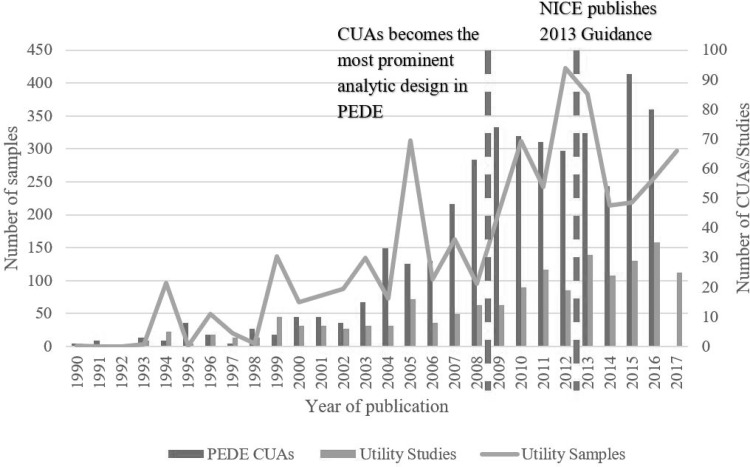
Number of studies and samples from 1990–June 2017 and CUAs in PEDE 1990–2016. *Note* Category 2017 denotes papers published up to 30th June 2017; PEDE: Paediatric Economic Database Evaluation; CUAs: cost–utility analyses; NICE: National Institute for Health and Care Excellence

Figure 3 jointly depicts trends in number of utility studies and samples and number of paediatric CUAs in the PEDE database. It also marks out years 2009 and 2013, which are the transition timepoints for the two separate periodic changes analysed below. The upward trends in all three groups are visible and confirmed by statistical tests for linear trend. The coefficients for year of publication were 1.15 (95% confidence interval: 0.96 to 1.36; *P* < 0.001) for number of utility studies, 12.70 (95% CI 9.32 to 16.08; *P* < 0.001) for number of utility samples and 3.63 (95% CI 3.01 to 4.25; *P* < 0.001) for number of PEDE CUAs. The number of utility studies increased at a steady rate across the whole review period, while the numbers of utility samples and PEDE CUAs increased markedly in the late 2000s.

#### Association between PEDE CUAs and utility studies and samples

Linear regression of the number of utility studies on number of CUAs, controlling for linear time trend, showed no association (coefficient on CUAs: 0.114; standard error 0.763; *P* value: 0.097). There was similarly no association between number of utility samples and number of CUAs (coefficient: 0.624; SE: 1.189; *P* value: 0.605). No associations were similarly found between proportion of paediatric economic evaluations that were CUAs and the number of primary utility studies and samples.

#### Periodic change in composition of utility studies and samples

Table 4 summarises the numbers of studies and samples by categories of study design, health condition, valuation method, target age, respondent type, administration mode and valuation of hypothetical health state. Supplementary Materials Tables S4-S7 present the results across 5-year publication intervals. Comparisons between two broader periods, 1990–2008 and 2009–June 2017, saw a significant decline in the proportion of patient case series (10.2% point decrease; *P* = 0.013).

**Table 4:** Periodic change in proportion of studies and samples in each study design and sample characteristic category

	Category	Number (%) of studies/samples			Test of proportion	
		1990–June 2017	1990–2008	2009–June 2017	Direction of change	*P* value
Study design	Patient case series^a^	47 (14.6)	24 (21.4)	25 (11.2)	**_**	**0.013**
	Cross-sectional survey^b^	171 (51.0)	57 (50.9)	114 (51.1)	+	0.972
	Longitudinal studies and RCTs without CUA^c^	47 (14.0)	14 (12.5)	33 (14.8)	+	0.568
	Longitudinal studies and RCTs with CUA and decision models with primary utility assessment^d^	68 (20.3)	17 (15.2)	51 (22.9)	+	0.099
	Total	335	112	223		
Health condition	General health	501 (12.6)	41 (2.9)	460 (18.0)	+	< **0.001**
	ICD-10 chapter 1: Infectious and parasitic diseases	195 (4.9)	143 (10.0)	52 (2.0)	**_**	< **0.001**
	ICD-10 chapter 2: Cancer	442 (11.1)	329 (23.1)	113 (4.4)	**_**	< **0.001**
	ICD-10 chapter 3: Diseases of the blood and immune system	85 (2.1)	49 (3.4)	36 (1.4)	_	< **0.001**
	ICD-10 chapter 4: Endocrine, nutritional and metabolic disorders	411 (10.3)	77 (5.4)	334 (13.1)	+	< **0.001**
	ICD-10 chapter 5: Mental and behavioural disorders	698 (17.6)	140 (9.8)	558 (21.9)	+	< **0.001**
	ICD-10 chapter 6: Nervous system disorders	114 (2.9)	18 (1.3)	96 (3.8)	+	< **0.001**
	ICD-10 chapter 7: Diseases of the eye	61 (1.5)	51 (3.6)	10 (0.4)	_	< **0.001**
	ICD-10 chapter 8: Diseases of the ear	158 (4.0)	70 (4.9)	88 (3.4)	_	**0.020**
	ICD-10 chapter 9: Circulatory system disorders	16 (0.4)	10 (0.7)	6 (0.2)	_	**0.014**
	ICD-10 chapter 10: Respiratory system disorders	192 (4.8)	37 (2.6)	155 (6.1)	+	< **0.001**
	ICD-10 chapter 11: Digestive system disorders	60 (1.5)	5 (0.4)	55 (2.2)	+	< **0.001**
	ICD-10 chapter 12: Diseases of the skin	26 (0.7)	19 (1.3)	7 (0.3)	_	< **0.001**
	ICD-10 chapter 13: Musculoskeletal system disorders	114 (2.9)	42 (3.0)	72 (2.8)	_	0.717
	ICD-10 chapter 14: Genitourinary system disorders	56 (1.4)	7 (0.5)	49 (1.9)	+	< **0.001**
	ICD-10 chapter 16: Conditions originating in the perinatal period	210 (5.3)	121 (8.5)	89 (3.5)	_	< **0.001**
	ICD-10 chapter 17: Congenital malformations	155 (3.9)	16 (1.1)	139 (5.4)	+	< **0.001**
	ICD-10 chapter 19: Injury, poisoning and other consequences of external causes	297 (7.5)	211 (14.8)	86 (3.4)	_	< **0.001**
	ICD-10 chapter 21: Contact with health services	80 (2.0)	10 (0.7)	70 (2.7)	+	< **0.001**
	Combined chronic diseases	103 (2.6)	27 (1.9)	76 (3.0)	+	**0.037**
	Total	3974	1423	2551		
Valuation method	Visual analogue scales^e^	832 (20.9)	230 (16.2)	602 (23.6)	+	< **0.001**
	Trade-off-based direct valuation methods^f^	541 (13.6)	337 (23.7)	204 (8.0)	_	< **0.001**
	Adult-specific MAUIs^g^	476 (12.0)	130 (9.1)	346 (13.6)	+	< **0.001**
	MAUIs compatible with childhood and adult populations^h^	1537 (38.7)	660 (46.4)	877 (34.4)	_	< **0.001**
	Childhood-specific MAUIs^i^	573 (14.4)	63 (4.4)	510 (20.0)	+	< **0.001**
	Utility from non-preference-based methods^j^	15 (0.4)	3 (0.2)	12 (0.5)	+	0.147
	Total	3974	1423	2551		
Respondent type	Self-assessment by children	1498 (37.7)	318 (22.3)	1180 (46.3)	+	< **0.001**
	Proxy assessment (includes joint assessment by proxies and children)	2476 (62.3)	1105 (77.7)	1371 (53.7)	_	
	Total	3974	1423	2551		
Administration mode	Self-administered surveys	2464 (62.0)	609 (42.8)	1855 (72.7)	+	< **0.001**
	Interview-administered surveys	1510 (38.0)	814 (57.2)	696 (27.3)	_	
	Total	3974	1423	2551		
Valuation of hypothetical state	Experienced health state	3247 (81.7)	1009 (70.9)	2238 (87.7)	+	< **0.001**
	Hypothetical health state	727 (18.3)	414 (29.1)	313 (12.3)	_	
	Total	3974	1423	2551		
Age of target population	Sample contains pre-adolescents (mean or median age below 12 or minimum age below 12 if mean/median age not reported)	2086 (52.5)	708 (49.8)	1378 (54.0)	+	**0.011**
	Sample does not contain pre-adolescents	1315 (33.1)	445 (31.3)	870 (34.1)	+	0.072
	Sample age not specified	573 (14.4)	270 (19.0)	303 (11.9)	_	< **0.001**
	Total	3974	1423	2551		

All values in bold are signify statistically significant results, i.e. *p* < 0.05

^a^Utility assessment carried out for a set period of time in a healthcare institution setting on attending patients or inpatients

^b^Surveys of varying modes of administration such as mail or internet

^c^Longitudinal observational studies include prospective and retrospective studies

^d^Cost–utility analyses alongside prospective or retrospective observational studies or RCTs and decision modelling studies which carry out primary collection of childhood utility data

^e^Stand-alone VAS, EQ-5D VAS and EQ-5D-Y VAS

^f^TTO, SG, Chained Gamble and adjusted SG

^g^EQ-5D, SF-6D, AQoL-5D and 15D

^h^QWB, HUI2, HUI3, modified HUI and ABC-UI

^i^EQ-5D-Y, CHU9D, 16D, 17D, AQoL-6D, PAHOM and CH-6D

^j^See [46–50]

In terms of health condition, comparisons between the two periods revealed significant changes in proportion of samples in all ICD-10 chapters except musculoskeletal system disorders. Categories that saw the greatest change were cancer (18.7% point decrease; *P* < 0.001), general health (15.1% point increase; *P* < 0.001), mental and behavioural disorders (12.1% point increase; *P* < 0.001) and injury (11.4% point decrease; *P* < 0.001).

There were equally significant changes for valuation method, respondent type, administration mode and valuation of hypothetical health states. For valuation method, childhood-specific MAUIs saw the greatest increase (15.6% point increase; *P* < 0.001) whilst trade-off-based direct valuation methods saw the greatest decrease (15.7% point decrease; *P* < 0.001). However, there was also a significant increase in the use of adult-specific MAUIs (4.5% point increase; *P* < 0.001) and VAS approaches (7.4% point increase; *P* < 0.001). MAUIs compatible with both childhood and adult populations experienced a significant decrease in use (12.0% point decrease; *P* < 0.001). There was a significant shift towards self-assessment of health status by children (24.0% point increase; *P* < 0.001), and the use of self-administered surveys in school and clinic settings (29.9% point increase; *P* < 0.001). Similarly, there was a significant increase in the number of samples valuing experienced rather than hypothetical health states (16.8% point increase; *P* < 0.001). As for age of target population, there was a significant shift in focus to pre-adolescent populations (4.2% point increase; *P* = 0.011) and a significant fall in the proportion of samples failing to provide information on age (7.1% point decrease; *P* < 0.001).

#### Influence of HTA guidance on primary samples

Table 5 presents similar tests of proportions comparing two periods, 1990–2012 (before the publication of the 2013 NICE HTA guidance) and 2013–June 2017, in 355 UK samples containing pre-adolescents (mean/median or minimum age 12 or below) and 207 UK samples not containing pre-adolescents. Samples containing pre-adolescents increased significantly as a proportion of all samples from 45.0% in 1990–2012 to 85.1% in 2013–June 2017 (40.1% point increase; *P* < 0.001). Seventy-two samples in 1990–2012 did not report age of target population compared to none in 2013–June 2017. The evidence supporting our hypothesis that publication of the NICE guidance would increase the use of reference instruments in relevant age groups and reduce the use of non-reference instruments was mixed. For samples containing pre-adolescents, the use of EQ-5D-Y increased from 3.9% of samples in 1990–2012 to 16.9% in 2013–June 2017 (13.0% point increase; *P* < 0.001). Moreover, the use of the non-reference HUI2/3 and trade-off-based instruments fell from 75.4% and 5.8% of samples, respectively, in 1990–2012 to no samples in 2013–June 2017. However, the use of EQ-5D also increased from 11.1 to 32.4% (21.3% point increase; *P* < 0.001), while the uses of the non-reference CHU9D (34.5% point increase; *P* < 0.001) and VAS approaches (12.3% point increase; *P* < 0.001) both increased.

**Table 5:** UK samples by valuation method and period before and after 2013 NICE guideline

Category	Valuation method	Number of samples (%)		Test of proportion	
		1990–2012	2013–June 2017	Direction of change	*P* value
Sample contains pre-adolescents^a^					
Reference for adult population	EQ-5D	23 (11.1)	48 (32.4)	+	< **0.001**
Reference for childhood population	EQ-5D-Y	8 (3.9)	25 (16.9)	+	< **0.001**
MAUIs compatible with childhood populations	HUI2	50 (24.2)	0 (0.0)		
	HUI3	106 (51.2)	0 (0.0)		
	Total	156 (75.4)	0 (0.0)	−	< **0.001**
Childhood-specific MAUIs	CHU9D	0 (0.0)	51 (34.5)	+	< **0.001**
VAS		8 (3.9)	24 (16.2)	+	< **0.001**
Trade-off direct valuation methods		12 (5.8)	0 (0.0)	−	**0.003**
Sub-total (% of total)		207 (45.0)	148 (85.1)	+	< **0.001**
Sample does not contain pre-adolescents					
Reference for adult population	EQ-5D	12 (6.6)	9 (34.6)	+	< **0.001**
Reference for childhood population	EQ-5D-Y	15 (8.3)	4 (15.4)	+	0.241
Adult-specific MAUIs	SF-6D	0 (0.0)	2 (7.7)	+	< **0.001**
MAUIs compatible with childhood populations	HUI2	11 (6.1)	2 (7.7)		
	HUI3	58 (32.0)	6 (23.1)		
	Total	69 (38.1)	8 (30.8)	−	0.471
VAS		38 (21.0)	3 (11.5)	−	0.256
Trade-off direct valuation methods		47 (26.0)	0 (0.0)	−	**0.003**
Sub-total (% of total)		181 (39.3)	26 (14.9)	−	< **0.001**
Sample did not report age of target population					
Sub-total (% of total)		72 (15.7)	0 (0.0)	−	< **0.001**
Total		460	174		

All values in bold are signify statistically significant results, i.e. *p* < 0.05

^a^Mean or median age below 12 or minimum age below 12 if mean/median age not reported; note that NICE in the UK recommends EQ-5D for adolescents aged 13 and over and EQ-5D-Y for children aged 7–12—this creates a subtle difference in the age range of pre-adolescence between this study and NICE guidance

#### Associations between methodological factors

Table 6 displays the association between valuation method, target age and three other methodological factors—respondent type, administration mode and valuation of hypothetical health states. In all four tests, there is significant evidence to reject the null hypothesis of no association, with *P* values all less than 0.001. Results suggest that VAS approaches are more likely to be used in pre-adolescent samples and to be assessed by children themselves using self-administered surveys. However, VAS approaches are also more likely to be used to assess hypothetical rather than experienced health states. Trade-off-based direct valuation methods are more likely to be used in older adolescent samples and/or involve proxy assessment. They are also more likely to be administered by interview and for assessing hypothetical health states. The associative patterns varied for the three categories of MAUIs. Adult-specific MAUIs were more likely to be used in adolescent samples, while MAUIs compatible with or specific to childhood populations were both more likely to be used in pre-adolescent samples. Adult-specific MAUIs were more likely to involve self-assessment by children, and MAUIs compatible with both populations more likely to involve proxy assessment. All MAUI categories were more likely to be self-administered than interviewer-administered and be used to assess experienced rather than hypothetical health states.

**Table 6:** Association between valuation method and (1) age of sample; (2) respondent type; (3) administration mode and (4) valuation of hypothetical states

	Visual analogue scales	Trade-off-based direct valuation methods	Adult-specific MAUIs	MAUIs compatible with childhood and adult populations	Childhood-specific MAUIs	Utility from non-preference-based methods	Total	Chi-square test statistic (*P* value)
(1) Age of sample								
Sample contains pre-adolescents*	486** **(471.7)**	152 (231.2)	250 (284.0)	**751** **(740.3)**	**443** **(349.6)**	4 (9.2)	2086	**154.4** **(**< **0.001)**
Sample does not contain pre-adolescents	283 (297.3)	**225** **(145.8)**	**213** **(179.0)**	456 (466.7)	127 (220.4)	**11** **(5.8)**	1315	5 degrees of freedom
Total	769	377	463	1207	570	15	3401	
(2) Respondent type								
Self-assessment by children	**353** **(313.6)**	140 (203.9)	**220** **(179.4)**	337 (579.4)	**439** **(216.0)**	**9** **(5.7)**	1498	**590.3** **(**< **0.001)**
Proxy assessment***	479 (518.4)	**401** **(337.1)**	256 (296.6)	**1200** **(957.6)**	134 (357.0)	6 (9.3)	2476	5 degrees of freedom
Total	832	541	476	1537	573	15	3974	
(3) Administration mode								
Self-administered surveys	**580** **(515.9)**	80 (335.4)	**321** **(295.1)**	**1036** **(953.0)**	**438** **(355.3)**	9 (9.3)	2464	**608.6** **(**< **0.001)**
Interview-administered surveys	252 (316.1)	**461** **(205.6)**	155 (180.9)	501 (584.0)	135 (217.7)	**6** **(5.7)**	1510	5 degrees of freedom
Total	832	541	476	1537	573	15	3974	
(4) Valuation of hypothetical states								
Experienced health states	582 (679.8)	152 (442.0)	**450** **(388.9)**	**1513** **(1255.8)**	**535** **(468.2)**	**15** **(12.3)**	3247	> **1500.0** **(**< **0.001)**
Hypothetical health states	**250** **(152.2)**	**389** **(99.0)**	26 (87.1)	24 (281.2)	38 (104.8)	0 (2.7)	727	5 degrees of freedom
Total	832	541	476	1537	573	15	3974	

All values in bold are signify statistically significant results, i.e. *p* < 0.05

*Samples with mean or median age below 12 or minimum age below 12 (if mean/median age not reported)

** Observed number of samples (Expected number of samples). Cells in bold denote cases where observed number is greater than expected

*** Includes joint assessment by proxies and children

## Discussion

This study represents the most comprehensive systematic review of primary studies reporting health utilities for childhood conditions, covering all studies published up to 30 June 2017. All ICD-10 chapters relevant to childhood health, 23 valuation methods, all childhood ages, 12 respondent types, 8 administration modes and 42 country settings were observed across 3974 samples from 335 studies. There were strong upward linear trends in numbers of utility studies and samples and PEDE CUAs. There was no statistically significant association between numbers of PEDE CUAs and utility studies and samples after controlling for linear trend. Adopting year 2009 (when CUA became the prominent analytic approach for paediatric economic evaluations [24]) as a key transition point, the study found evidence of significant changes in composition of primary samples across health condition, valuation method and other methodological factors. There was also evidence of more refined approaches in primary utility research as reflected by greater pre-adolescent population coverage, target age reporting and valuation of experienced health states. For a subset of UK samples and using the year 2013 as a transition point (when NICE HTA guidance [2] was published), there was weak evidence that primary utility research adhered to national guidance. Finally, tests of association found that sample age, respondent type, administration mode and valuation of hypothetical health state varied significantly by valuation method.

Previous systematic reviews of primary studies assessing childhood health utilities have largely focused on specific diseases [27–31] or valuation methods [32]. Reviews that assembled data across heterogenous childhood health conditions, valuation methods and other methodological factors are few. A review by Tarride and colleagues [54] included 77 studies measuring utilities published before 2008, but limited its scope to asthma, cancer, combined chronic diseases, diabetes and skin disease and to a limited number of valuation methods, namely HUI, EQ-5D, SG and TTO. Temporal patterns in included studies and samples were not explored. A review by Thorrington and Eames [55] included 90 studies published in 1994–2013 measuring childhood health utilities using direct or indirect valuation methods. Temporal patterns in the data led to similar findings to this review: adult-specific valuation methods increased in use despite the availability of childhood-specific measures, whilst childhood-derived tariffs were seldom applied. However, the data were not categorised by health condition or age of sample, and statistical tests were not performed to evaluate the variations over time or associations between factors. Finally, a review by Kwon and colleagues [38] used the same search strategy to identify 272 studies published by 31 December 2015, thus overlapping significantly in data sources with this study. However, their descriptive and statistical analyses focused on non-temporal patterns in studies and samples and on associations between mean utility values and health conditions and methodological factors through meta-regression. Moreover, no comparison was made to PEDE CUAs, whilst the impact of HTA methods guidance on primary research was not assessed.

To our knowledge, this review is the first to summarise the distribution of all mean and median utility scores relevant to childhood health. The results show a clear disparity in distributional features between alternative central statistics of mean and median utilities. The ceiling effect, known to be significant in distributions of individual-level utility data [56], was visible in the distribution of medians but small in that of means. Negative skew in individual-level utility would produce downward adjustment of means relative to medians, and this is likely reflected in the less pronounced ceiling effect in the distribution of means. Caution is thus warranted when using aggregate or pooled outcomes in economic evaluations since they contain heterogeneous distributional features depending on central statistic chosen, which may also differ from the distribution of individual-level data [57]. The distributional skew also varied by valuation method, with means and medians from trade-off-based direct valuation methods (TTO, SG and variants) concentrated at the upper end of the utility range. This is consistent with several meta-regression studies [58–61] on health utilities for diverse conditions, which also show higher utilities from TTO and SG than from other valuation approaches, potentially due to biases in making trade-offs such as loss and risk aversions [62].

The review is also the first to categorise all primary samples by ICD-10 chapter and code and document their compositional changes over time. Ascertaining how well the distribution of utility samples by ICD-10 chapter reflects prevalence of health needs in children is of interest. Were and colleagues [63] noted transitions in childhood morbidity patterns from infectious diseases to congenital anomalies, injuries and non-communicable diseases, including obesity, diabetes, cancer and respiratory diseases, while Laski and colleagues [64] highlighted mental health as an emerging priority for adolescent populations. These observations are reflected in our review data: the proportion of samples for infectious diseases fell across two primary time periods, 1990–2008 and 2009–June 2017, while the proportions for endocrine, nutritional and metabolic disorders, mental and behavioural disorders, respiratory system disorders and congenital malformations increased significantly, although those for cancer and injuries fell. The distribution of samples by health condition can also be compared to the focus of paediatric CUAs. Although the latter may reflect policy interest more than underlying health needs, collection of primary data should support such secondary research. The report by Sullivan and Ungar [65] catalogued the distribution of paediatric CUAs by ICD-9/10 chapter. Infectious diseases remained the dominant topic, accounting for 40.6% of all paediatric CUAs between 1980 and 2013. This was followed by nervous system disorders (8.9%), respiratory system disorders (7.8%) and diseases of the blood (5.9%). This contrasts with our observations for utility data, with the above categories only accounting for 4.9%, 2.9%, 4.8% and 2.1% of samples, respectively. Mental health accounted for the largest share of utility samples (17.6%) but just 4.3% of paediatric CUAs. The lack of concordance is consistent with the lack of association found between the number of paediatric CUAs and numbers of utility studies and samples, controlling for linear trend. Future studies should assess the extent to which primary utility data facilitate CUAs and the extent to which CUAs motivate primary utility research.

Another contribution of the review is documenting the change in patterns of valuation method over time. A clear pattern was visible in the movement away from trade-off-based direct valuation methods towards MAUIs that require only simple responses on a classification system and separate valuations using tariffs derived from representative populations. The proportion of samples using trade-off-based direct valuation methods fell from 23.7% in 1990–2008 to 8.0% in 2009–June 2017, whereas the proportion of samples using MAUIs of any type increased from 59.9% in 1990–2008 to 67.9% in 2009–June 2017. This perhaps reflects the concern over feasibility of applying cognitively demanding assessments in children [15], as well as the increasing number of national guidelines that recommend MAUIs over direct valuation methods [2–6]. The most significant growth occurred for childhood-specific MAUIs, which increased from 4.4% of samples in 1990–2008 to 20.0% in 2009–June 2017. Tests of association also showed that childhood-specific MAUIs were more likely to be self-assessed, self-administered and focussed on experienced health states. The question remains as to whether this represents an unambiguous improvement in childhood health utility assessment. Childhood-specific MAUIs have psychometric properties suited to children [15–19] and are recommended by some decision-making bodies [2, 6]. However, this raises a concern over comparability of their utilities to those of adult-specific MAUIs, particularly when used as inputs into life-course decision models. Moreover, a review of childhood-specific MAUIs found no study that maps across childhood- and adult-specific utilities [22].

The above concern over comparability may explain the finding that the publication of HTA guidance by NICE had a mixed effect on primary utility assessments. In the UK samples containing pre-adolescent populations, the proportion using the adult-specific EQ-5D increased by more than the proportion using the childhood-specific EQ-5D-Y after the guidance publication in 2013. Adlard and colleagues [66], after reviewing 43 paediatric CUAs set in the UK and published between 2004 and 2010, noted the paucity of childhood-specific utility data, and recommended increased funding for primary research that would follow NICE guidance. There is only weak evidence that this has occurred since 2013. It is conceivable that NICE guidance may have encouraged the use of an adult-specific reference instrument (i.e. EQ-5D) in both primary and secondary research, particularly for life-course models that prioritise comparability of utility scores over the whole age spectrum.

This study also catalogues all tariffs applied to MAUI-based studies, the population from whom they were derived and their valuation method. Around one-quarter (23.7%) of samples using MAUIs provided no information on the tariff applied, and this proportion increased from 19.2% in 1990–2008 to 26.0% in 2009–June 2017. Among samples that used MAUIs compatible with or specific to childhood populations, 60.0% applied adult-derived tariffs, and this proportion increased from 50.3% in 1990–2008 to 64.6% in 2009–June 2017. Only one sample using the EQ-5D-Y applied childhood-derived tariffs, while none was available for the HUI3. However, it is difficult to conclude that application of childhood-derived tariffs is more appropriate than adult-derived tariffs even for MAUIs compatible with or specific to childhood populations. Children are not autonomous legal, social and economic agents and do not bear the burden of financing public healthcare. Hence their preferences, even when conflicting with those of the general adult public, may arguably be of less importance. That said, analysts should recognise that applications of childhood and adult-derived tariffs may produce enough variation in utility values to alter policy decisions [67]. They should therefore include utility values from both tariffs where they are available in sensitivity analysis to observe the impact of alternative tariff populations.

One potential-related research area is assessing the quality of utility research and its improvement over the review period. However, the tests of association between sample-level characteristics showed that choice in one methodological area (e.g. use of proxy assessment) was strongly associated with other methodological choices (e.g. use of cognitively challenging trade-off-based method) and target population age, which makes it challenging to assess quality and its improvement in terms of methodological choices (e.g. the proportion of samples using proxy assessment). This is more so in the absence (to our knowledge) of any validated quality assessment tool for primary utility studies. That said, the increase in the proportion of samples reporting target population age over the review period was an unambiguous improvement, as was the decrease in the proportion of samples valuing hypothetical health states that may result in significant overestimation of disease burden relative to valuation of experienced health states in childhood [38]. There were also areas of concern. First, there was a lack of studies and samples set in lower and middle-income countries (LMICs). Only 8.2% of samples were from LMICs, consistent with the paucity of paediatric CUAs in LMICs, despite their substantially higher paediatric disease burdens [68, 91]. Among LMIC samples, 28.4% used VAS measures despite concerns over their validity for QALY construction [39], a proportion higher than for all samples (20.9%). Second, among the samples using MAUIs, 48.5% applied tariffs derived from populations foreign to the primary study setting, though the proportion fell from 51.7% in 1990–2008 to 46.9% in 2009–June 2017. Among LMIC samples that used MAUIs, this proportion was 75.5%. Clearly, there is a pressing need for more national tariff derivation studies, and for more accurate reporting of tariff information in publications.

There are several limitations to the study. First, some eligible studies may have been missed during the systematic review process, even though the search strategies were extensively piloted to maximise sensitivity. Second, several studies did not report or did not clearly specify important covariates, such as age and comorbidities, which may affect utility values. Third, the broad criteria for selection of valuation methods resulted in inclusion of modified forms of methods (e.g. for TTO, SG and HUI) and preference-based disease-specific instruments (e.g. PAHOM and ABC-UI), which complicated between-method comparisons. Fourth, the choice of 2009 as a transition point for all studies and samples was not based on potential causal or associative mechanisms determining the design and volume of primary utility research. Finally, cross-tabulations between methodological factors were limited to two-way Chi-square tests for association, and further research should consider application of multivariate regressions.

## Conclusion

This systematic review reveals significant growth in both volume and diversity of childhood utility studies over the past three decades. HTA agencies should note the weak adherence to guidance concerning the use of reference case valuation methods by primary studies. There is a need for studies that derive tariffs in settings relevant to primary utility collection. Geographic coverage of utility assessment is heavily skewed to high-income countries, and further research in LMICs is pressing.

## Electronic supplementary material

Below is the link to the electronic supplementary material.

Supplementary material 1 **ONLINE RESOURCE** Excel database containing information on all 3,974 sub-samples of health utilities and descriptors can be found online at http://childhoodutilities.wordpress.com. The website also contains the detailed search strategy and a guidance note for Excel navigation. (DOCX 162 KB)

## References

[1] Drummond MF, Sculpher MJ, Claxton K, Stoddart GL, Torrance GW (2015). Methods for the economic evaluation of health care programmes.

[2] NICE (2013). Guide to the methods of technology appraisal 2013. 27905712

[3] SMC (2017). Working with SMC—A guide for manufacturers.

[4] CADTH (2017). Guidelines for the economic evaluation of health technologies: Canada 4th Edition.

[5] PBAC (2015). Guidelines for preparing submissions to the Pharmaceutical Benefits Advisory Committee (Version 4.5).

[6] Neumann PJ, Sanders GD, Russell LB, Siegel JE, Ganiats TG (2016). Cost-effectiveness in health and medicine.

[7] Torrance GW, Feeny D (1989). Utilities and quality-adjusted life years. International Journal of Technology Assessment in Health Care.

[8] Dolan P, Gudex C, Kind P, Williams A (1996). Valuing health states: A comparison of methods. Journal of Health Economics.

[9] Brooks R (1996). EuroQol: The current state of play. Health Policy.

[10] Torrance GW, Furlong W, Feeny D, Boyle M (1995). Multi-attribute preference functions. Health Utilities Index. Pharmacoeconomics.

[11] Brazier J, Roberts J, Deverill M (2002). The estimation of a preference-based measure of health from the SF-36. Journal of Health Economics.

[12] Kaplan RM, Bush JW, Berry CC (1976). Health status: Types of validity and the index of well-being. Health Services Research.

[13] Hawthorne G, Richardson J, Osborne R (1999). The Assessment of Quality of Life (AQoL) instrument: A psychometric measure of health-related quality of life. Quality of Life Research.

[14] Ungar, W., & Gerber, A. (2010). The uniqueness of child health and challenges to measuring costs and consequences. *Economic Evaluation in Child Health*, 3–32.

[15] Ungar WJ (2011). Challenges in health state valuation in paediatric economic evaluation: Are QALYs contraindicated?. Pharmacoeconomics.

[16] Petrou S (2003). Methodological issues raised by preference-based approaches to measuring the health status of children. Health Economics.

[17] Ravens-Sieberer U, Wille N, Badia X, Bonsel G, Burstrom K, Cavrini G (2010). Feasibility, reliability, and validity of the EQ-5D-Y: Results from a multinational study. Quality of Life Research.

[18] Apajasalo M, Sintonen H, Holmberg C, Sinkkonen J, Aalberg V, Pihko H (1996). Quality of life in early adolescence: A sixteen-dimensional health-related measure (16D). Quality of Life Research.

[19] Apajasalo M, Rautonen J, Holmberg C, Sinkkonen J, Aalberg V, Pihko H (1996). Quality of life in pre-adolescence: A 17-dimensional health-related measure (17D). Quality of Life Research.

[20] Richardson JR, Peacock SJ, Hawthorne G, Iezzi A, Elsworth G, Day NA (2012). Construction of the descriptive system for the Assessment of Quality of Life AQoL-6D utility instrument. Health Qual Life Outcomes.

[21] Stevens KJ (2010). Working with children to develop dimensions for a preference-based, generic, pediatric, health-related quality-of-life measure. Qualitative Health Research.

[22] Chen G, Ratcliffe J (2015). A review of the development and application of generic multi-attribute utility instruments for paediatric populations. Pharmacoeconomics.

[23] Montgomery SM, Kusel J (2016). The prevalence of child-specific utilities in NICE appraisals for paediatric indications: Rise of the economic orphans?. Expert Review of Pharmacoeconomics & Outcomes Research.

[24] Sullivan SM, Tsiplova K, Ungar WJ (2016). A scoping review of pediatric economic evaluation 1980–2014: Do trends over time reflect changing priorities in evaluation methods and childhood disease?. Expert Review of Pharmacoeconomics & Outcomes Research.

[25] Kromm SK, Bethell J, Kraglund F, Edwards SA, Laporte A, Coyte PC (2012). Characteristics and quality of pediatric cost-utility analyses. Quality of Life Research.

[26] Weinstein MC, O’Brien B, Hornberger J, Jackson J, Johannesson M, McCabe C (2003). Principles of good practice for decision analytic modeling in health-care evaluation: Report of the ISPOR Task Force on Good Research Practices–Modeling Studies. Value Health.

[27] van Litsenburg RR, Kunst A, Huisman J, Ket JC, Kaspers GJ, Gemke RJ (2014). Health status utilities in pediatrics: A systematic review of acute lymphoblastic leukemia. Medical Decision Making.

[28] Pickard AS, Topfer LA, Feeny DH (2004). A structured review of studies on health-related quality of life and economic evaluation in pediatric acute lymphoblastic leukemia. JNCI Monographs.

[29] Kua WS, Davis S (2016). Systematic review of health state utilities in children with asthma. Value in Health.

[30] Janssens A, Rogers M, Gumm R, Jenkinson C, Tennant A, Logan S (2016). Measurement properties of multidimensional patient-reported outcome measures in neurodisability: A systematic review of evaluation studies. Developmental Medicine & Child Neurology.

[31] Brown V, Tan EJ, Hayes AJ, Petrou S, Moodie ML (2018). Utility values for childhood obesity interventions: A systematic review and meta-analysis of the evidence for use in economic evaluation. Obesity Reviews.

[32] Noyes J, Edwards RT (2011). EQ-5D for the assessment of health-related quality of life and resource allocation in children: A systematic methodological review. Value Health.

[33] Mittmann N, Trakas K, Risebrough N, Liu BA (1999). Utility scores for chronic conditions in a community-dwelling population. Pharmacoeconomics.

[34] Carroll AE, Downs SM (2009). Improving decision analyses: Parent preferences (utility values) for pediatric health outcomes. The Journal of Pediatrics.

[35] Ungar WJ, Santos MT (2003). The pediatric economic database evaluation (PEDE) project: Establishing a database to study trends in pediatric economic evaluation. Medical Care.

[36] Ungar WJ (2016). The pediatric economic database evaluation (PEDE) project.

[37] Moher D, Liberati A, Tetzlaff J, Altman DG, Group P (2009). Preferred reporting items for systematic reviews and meta-analyses: The PRISMA statement. Annals of Internal Medicine.

[38] Kwon J, Kim SW, Ungar WJ, Tsiplova K, Madan J, Petrou S (2018). A systematic review and meta-analysis of childhood health utilities. Medical Decision Making.

[39] Parkin D, Devlin N (2006). Is there a case for using visual analogue scale valuations in cost-utility analysis?. Health Economics.

[40] WorldBank (2018). Low & middle income countries.

[41] Juniper EF, Guyatt GH, Feeny DH, Griffith LE, Ferrie PJ (1997). Minimum skills required by children to complete health-related quality of life instruments for asthma: Comparison of measurement properties. European Respiratory Journal.

[42] Oostenbrink R, HA AM, Essink-Bot ML (2002). The EQ-5D and the Health Utilities Index for permanent sequelae after meningitis: A head-to-head comparison. Journal of Clinical Epidemiology.

[43] Raspa M, Sacco P, Candrilli SD, Bishop E, Petrillo J (2016). Validity of a condition specific outcome measure for fragile X syndrome: The Aberrant Behaviour Checklist-utility index. Journal of Intellectual Disability Research.

[44] Chiou CF, Weaver MR, Bell MA, Lee TA, Krieger JW (2005). Development of the multi-attribute Pediatric Asthma Health Outcome Measure (PAHOM). International Journal for Quality in Health Care.

[45] Kang E (2016). Validity of child health-6 dimension(Ch-6d) for adolescents. Value in Health.

[46] Lynch FL, Hornbrook M, Clarke GN, Perrin N, Polen MR, O’Connor E (2005). Cost-effectiveness of an intervention to prevent depression in at-risk teens. Archives of General Psychiatry.

[47] Domino ME, Burns BJ, Silva SG, Kratochvil CJ, Vitiello B, Reinecke MA (2008). Cost-effectiveness of treatments for adolescent depression: Results from TADS. The American Journal of Psychiatry.

[48] Domino ME, Foster EM, Vitiello B, Kratochvil CJ, Burns BJ, Silva SG (2009). Relative cost-effectiveness of treatments for adolescent depression: 36-week results from the TADS randomized trial. Journal of the American Academy of Child and Adolescent Psychiatry.

[49] McBain RK, Salhi C, Hann K, Salomon JA, Kim JJ, Betancourt TS (2016). Costs and cost-effectiveness of a mental health intervention for war-affected young persons: decision analysis based on a randomized controlled trial. Health Policy and Planning.

[50] Hartman JD, Craig BM, Blackburn C, Simmons V (2016). The Association between Maternal Smoking during Pregnancy and Child Quality-Adjusted Life Years. Value in Health.

[51] Barr RD, Furlong W, Dawson S, Whitton AC, Strautmanis I, Pai M (1993). An assessment of global health status in survivors of acute lymphoblastic leukemia in childhood. The American Journal of Pediatric Hematology/Oncology.

[52] Barr R, Pai M, Weitzman S, Feeny D, Furlong W, Rosenbaum P (1994). A multiattribute approach to health-status measurement and clinical management illustrated by an application to brain-tumors in childhood. International Journal of Oncology.

[53] Lee D, Gladwell D, Batty AJ, Brereton N, Tate E (2013). The cost effectiveness of licensed oromucosal midazolam (Buccolam((R))) for the treatment of children experiencing acute epileptic seizures: An approach when trial evidence is limited. Pediatric Drugs.

[54] Tarride JE, Burke N, Bischof M, Hopkins RB, Goeree L, Campbell K (2010). A review of health utilities across conditions common in paediatric and adult populations. Health and Quality of Life Outcomes.

[55] Thorrington D, Eames K (2015). Measuring health utilities in children and adolescents: A systematic review of the literature. PLoS ONE.

[56] Brazier J, Roberts J, Tsuchiya A, Busschbach J (2004). A comparison of the EQ-5D and SF-6D across seven patient groups. Health Economics.

[57] Petrou S, Kwon J, Madan J (2018). A practical guide to conducting a systematic review and meta-analysis of health state utility values. Pharmacoeconomics.

[58] Peasgood T, Ward SE, Brazier J (2010). Health-state utility values in breast cancer. Expert Review of Pharmacoeconomics & Outcomes Research.

[59] McLernon D, Dillon J, Donnan P (2006). Health-state utilities in liver disease: A systematic review and meta-analysis. Value in Health.

[60] Wyld M, Morton RL, Hayen A, Howard K, Webster AC (2012). A systematic review and meta-analysis of utility-based quality of life in chronic kidney disease treatments. PLoS Medicine.

[61] Bremner KE, Chong CA, Tomlinson G, Alibhai SM, Krahn MD (2007). A review and meta-analysis of prostate cancer utilities. Medical Decision Making.

[62] Van Osch SM, Wakker PP, Van Den Hout WB, Stiggelbout AM (2004). Correcting biases in standard gamble and time tradeoff utilities. Medical Decision Making.

[63] Were WM, Daelmans B, Bhutta Z, Duke T, Bahl R, Boschi-Pinto C (2015). Children’s health priorities and interventions. BMJ.

[64] Laski L, Expert Consultative Group for Every Woman Every Child on Adolescent (2015). Realising the health and wellbeing of adolescents. BMJ.

[65] Sullivan, S. M., & Ungar, W. J. (2016). Will the growth spurt continue? Trends in child health economic evaluation: 1980 to 2013.

[66] Adlard N, Kinghorn P, Frew E (2014). Is the UK NICE “Reference Case” influencing the practice of pediatric quality-adjusted life-year measurement within economic evaluations?. Value in Health.

[67] Ratcliffe J, Stevens K, Flynn T, Brazier J, Sawyer MG (2012). Whose values in health? An empirical comparison of the application of adolescent and adult values for the CHU-9D and AQOL-6D in the Australian adolescent general population. Value in Health.

[68] Ungar, W. J., & Zur, R. M. (2015). Health economic evaluation for improving child health in low-and middle-income countries. In *Optimizing treatment for children in the developing world* (pp. 213–224). Berlin: Springer.

[69] Moodie M, Richardson J, Rankin B, Iezzi A, Sinha K (2010). Predicting time trade-off health state valuations of adolescents in four Pacific countries using the Assessment of Quality-of-Life (AQoL-6D) instrument. Value Health.

[70] Richardson J, Atherton Day N, Peacock S, Iezzi A (2004). Measurement of the quality of life for economic evaluation and the Assessment of Quality of Life (AQoL) Mark 2 instrument. Australian Economic Review.

[71] Ratcliffe J, Flynn T, Terlich F, Stevens K, Brazier J, Sawyer M (2012). Developing adolescent-specific health state values for economic evaluation: an application of profile case best-worst scaling to the Child Health Utility 9D. Pharmacoeconomics.

[72] Ratcliffe J, Huynh E, Chen G, Stevens K, Swait J, Brazier J (2016). Valuing the Child Health Utility 9D: Using profile case best worst scaling methods to develop a new adolescent specific scoring algorithm. Social Science & Medicine.

[73] Stevens K, Brazier J, McKenna S, Doward L, Cork M (2005). The development of a preference-based measure of health in children with atopic dermatitis. British Journal of Dermatology.

[74] Wu X, Ohinmaa A, Johnson J, Veugelers P (2014). Assessment of children’s own health status using visual analogue scale and descriptive system of the EQ-5D-Y: Linkage between two systems. Quality of Life Research.

[75] Dolan P (1997). Modeling valuations for EuroQol health states. Medical Care.

[76] Lamers LM, Stalmeier PF, McDonnell J, Krabbe PF, van Busschbach JJ (2005). [Measuring the quality of life in economic evaluations: the Dutch EQ-5D tariff]. Ned Tijdschr Geneeskd.

[77] Oppe M, Devlin NJ, Szende A (2007). EQ-5D value sets: Inventory, comparative review and user guide.

[78] Viney R, Norman R, King MT, Cronin P, Street DJ, Knox S (2011). Time trade-off derived EQ-5D weights for Australia. Value Health.

[79] Chevalier J, de Pouvourville G (2013). Valuing EQ-5D using Time Trade-Off in France. European Journal of Health Economics.

[80] Burström K, Sun S, Gerdtham U-G, Henriksson M, Johannesson M, Levin L (2014). Swedish experience-based value sets for EQ-5D health states. Quality of Life Research.

[81] Greiner W, Weijnen T, Nieuwenhuizen M, Oppe S, Badia X, Busschbach J (2003). A single European currency for EQ-5D health states. Results from a six-country study. European Journal of Health Economics.

[82] Shaw JW, Johnson JA, Coons SJ (2005). US valuation of the EQ-5D health states: development and testing of the D1 valuation model. Medical Care.

[83] Gerald JK, McClure LA, Harrington KF, Moore T, Hernandez-Martinez AC, Gerald LB (2012). Measurement characteristics of the pediatric asthma health outcome measure. Journal of Asthma.

[84] Torrance GW, Feeny DH, Furlong WJ, Barr RD, Zhang Y, Wang Q (1996). Multiattribute utility function for a comprehensive health status classification system: Health Utilities Index Mark 2. Medical Care.

[85] Cadman D, Goldsmith C, Torrance G, Boyle M, Furlong W (1986). Development of a health status index for Ontario children. Final Report to the Ontario Ministry of Health on Research Grant DM.

[86] Wang QN, Furlong W, Feeny D, Torrance G, Barr R (2002). How robust is the Health Utilities Index Mark 2 utility function?. Medical Decision Making.

[87] McCabe CJ, Stevens KJ, Brazier JE (2005). Utility scores for the health utilities index mark 2—An empirical assessment of alternative mapping functions. Medical Care.

[88] Feeny D, Furlong W, Torrance GW, Goldsmith CH, Zhu Z, DePauw S (2002). Multiattribute and single-attribute utility functions for the health utilities index mark 3 system. Med Care.

[89] Kaplan RM, Anderson JP, Wu AW, Mathews WC, Kozin F, Orenstein D (1989). The Quality of Well-being Scale. Applications in AIDS, cystic fibrosis, and arthritis. Medical Care.

[90] Kerr C, Breheny K, Lloyd A, Brazier J, Bailey DB, Berry-Kravis E (2015). Developing a utility index for the Aberrant Behavior Checklist (ABC-C) for fragile X syndrome. Quality of Life Research.

[91] Ungar W (2007). Paediatric health economic evaluations: a world view. Healthcare Quarterly.

