# Supplementary material for: Patterns, trends and methodological associations in the measurement and valuation of childhood health utilities
Source: Qual Life Res. 2019 Feb 19;28(7):1705–24. doi: 10.1007/s11136-019-02121-z (PMC6571090; doi:10.1007/s11136-019-02121-z)
Supplement: Supplementary file 1 — Supplementary material 1 ONLINE RESOURCE Excel database containing information on all 3,974 sub-samples of health utilities and descriptors can be found online at http://childhoodutilities.wordpress.com. The website also contains the detailed search strategy and a guidance note for Excel navigation. (DOCX 162 KB) [file 11136_2019_2121_MOESM1_ESM.docx]

**Patterns, trends and methodological associations in the measurement and valuation of childhood health utilities – Supplementary Materials**

Joseph Kwon, MSc,^1^ Sung Wook Kim, PhD,^2^ Wendy J. Ungar, MSc, PhD,^3,4^ Kate Tsiplova, MSc,^3^ Jason Madan, PhD,^2^ Stavros Petrou, PhD.^2^

^1^ School of Health and Related Research, University of Sheffield, Sheffield, UK;

^2^ Warwick Medical School, University of Warwick, Coventry, UK;

^3^ Program of Child Health Evaluative Sciences, The Hospital for Sick Children Research Institute, Toronto, Canada;

^4^ Institute of Health Policy, Management and Evaluation, University of Toronto, Toronto, Canada.

**Address correspondence to**:

Professor Stavros Petrou, Warwick Clinical Trials Unit, Warwick Medical School, Gibbet Hill Road, University of Warwick, Coventry CV4 7AL, UK.

Email: [s.petrou@warwick.ac.uk](mailto:s.petrou@warwick.ac.uk)

Telephone: +44(0) 2476 151124.

| **Table S1.** Search strategy | | |
| --- | --- | --- |
| Database: PubMed, Embase, Web of Science, PsycINFO, EconLit, CINAHL, Cochrane Library | | |
| Date of search: (i) 26^th^ January 2016 for articles from database inception to 31^st^ December 2015; (ii) 1^st^ July 2017 for articles from 1^st^ January 2016 to 30^th^ June 2017 | | |
| Limit search to title and abstract and English articles | | |
| **Search Category** |  | **Search Terms** |
| **Utility Terms** | 1 | Utilit* or disutilit* or HSUV |
|  | 2 | “quality adjusted life year*” or QALY or “quality-adjusted life year*” or “quality-adjusted life-year*” |
|  | **3** | **OR (1 to 2)** |
| **Indirect Valuation Method Terms** | 4 | EQ-5D or “EQ 5D” or EQ5D or Euroqol or “Euro qol” or EQ-5D-Y or "EQ 5D Y" |
|  | 5 | Short-form survey-6D or short form 6D or SF-6D or “SF 6D” or SF6D |
|  | 6 | “health utilities index” |
|  | 7 | “quality of well being” or “quality of well-being” or QWB |
|  | 8 | 16D Health-Related Quality of Life or 16D HRQoL or 17D Health-Related Quality of Life or 17D HRQoL |
|  | 9 | AQoL-6D or Assessment of Quality of Life-6D |
|  | 10 | “Child Health Utility 9 Dimension” or CHU9D or CHU-9D or “CHU 9D” |
|  | 11 | Adolescent Health Utility Measure or AHUM |
|  | 12 | 15-dimensional instrument or 15 dimensional instrument |
|  | 13 | preference-based measure of HRQoL or preference based measure of HRQoL |
|  | 14 | multi-attribute utility instrument or multiattribute utility instrument |
|  | **15** | **OR (4 to 14)** |
| **Direct Valuation Method Terms** | 16 | Standard Gamble or standard-gamble |
|  | 17 | Time trade off or time trade-off |
|  | 19 | best worst scaling or best-worst scaling |
|  | 19 | Discrete choice experiment or discrete-choice experiment |
|  | 20 | person trade off or person trade-off |
|  | 21 | scoring algorithm or scoring-algorithm |
|  | 22 | utility elicitation or direct elicitation |
|  | **23** | **OR (16 to 22)** |
|  | **24** | **3 OR 15 OR 23** |
| **Childhood Terms** | 25 | Child* or adolesc* or kid or kids or youngster* or teen* or youth* or infant* or newborn* or perinat* or neonat* or “parent proxy” |
|  | 26 | Pediatri* or paediatri* |
|  | **27** | **OR (25 to 26)** |
| **Main Search** | **28** | **24 AND 27** |
|  | **29** | **Remove non-English Title and/or Abstract** |
|  | **30** | **Remove Duplicates Across Databases** |

| **Table S2.** Variables extracted from included studies using proforma | |
| --- | --- |
| **Variable** | **Note** |
| Bibliographic details | Author(s), title, year of publication |
| Country/geographical setting |  |
| Study setting | Hospital inpatient ward, hospital outpatient clinic, general practice, school, home or other |
| Health descriptor(s) | Health condition, disease, health state and/or intervention descriptor |
| Respondent type | Self-assessment by children or proxy-assessment by parents, caregivers, nurses, physicians or other proxies |
| Sample size | Including that of sub-samples (e.g., by age, gender or socioeconomic status) |
| Direct valuation method applied | If applicable |
| Indirect valuation method applied | If applicable |
| Tariff set | If indirect valuation method applied |
| Utility or VAS score | Including central statistics and measures of variability |
| Study design | Patient case series, cross-sectional study, longitudinal observational study (prospective or retrospective), randomised controlled trial (with or without economic evaluation), decision-analytic modelling or other |
| Administration mode | Self-administration in clinic or school setting, by postal survey or by online survey or interview administration by face-to-face or by telephone |
| Valuation of hypothetical health state |  |
| Response quality | Response rate, information on dropouts, reasons for loss to follow-up |
| Statistical methods for analysing utilities |  |
| Any reported methodological concerns | E.g., reliance of proxy respondents, low response rate, sample selection bias or other |

| **Table S3.** Characteristics of studies included in the systematic review | | | | | | |
| --- | --- | --- | --- | --- | --- | --- |
| **Reference** | **Health Condition** | **Intervention** | **Country of study population (of tariff population)** | **Valuation method** | **Respondent type and administration mode** | **Age of study population** |
| Aldebeyan et al (2017) [1] | Hypothetical adolescent idiopathic scoliosis |  | Canada | VAS, TTO, SG | Proxy-assessed by general public. Online survey | 12-18 |
| Anderson et al (2014) [2] | Risk of depression | Treatment: CBT in classroom  Control: usual school provision of health education | UK (UK) | EQ-5D-3L | Self-assessed by children. Self-administered paper questionnaire | 12-16 |
| Angelis et al (2016) [3] | Epidermolysis bullosa | Outpatient care | Bulgaria, France, Germany, Hungary, Italy, Spain, Sweden, UK | EQ-5D VAS | Self-assessed by children.  Postal survey | Mean: 7.2;  0-18 |
| Apajasalo et al (1996) [4] | Congenital skeletal dysplasia; End-stage heart, liver, kidney failure; Epilepsy; General health (control) | Patients with organ failure awaiting transplantation | Finland (Finland) | 16D | Self-assessed by children. Self-administered paper questionnaire | 12-15 |
| Apajasalo et al (1996b) [5] | Congenital skeletal dysplasia; End-stage heart, liver, kidney failure; Epilepsy; General health (control) | Patients with organ failure awaiting transplantation | Finland (Finland) | 17D | Self-assessed by children. Face-to-face interview | 8-11 |
| Apajasalo et al (1998) [6] | Congenital skeletal dysplasia |  | Finland (Finland) | 16D | Self-assessed by children. Self-administered paper questionnaire | 12-15 |
| Arkkila et al (2009) [7] | Receptive language disorder; General health (control) |  | Finland (Finland) | 16D | Self-assessed by children. Postal survey | Mean: 14.2; 12-17 |
| Arkkila et al (2011) [8] | Receptive language disorder; General health (control) |  | Finland (Finland) | 17D | Assessed together by children and parents. Postal survey | Mean: 10.1; 8-12 |
| Arnold et al (2014) [9] | Sickle cell disease; General health (control) | Stem cell transplantation | USA (Unspecified) | EQ-5D-3L VAS,  EQ-5D-3L | Self-assessed by children. Unspecified mode of administration | 12-14 |
| Autti-Ramo et al (2005) [10] | Hypothetical health states: Congenital adrenal hyperplasia; Glutaricaciduria type 1; LCHADD; MCADD; Phenylketonuria | Tandem mass spectrometry;  Screening for metabolic disease | Finland (Finland) | 16D | Proxy-assessed by physicians. Self-administered paper questionnaire | 0-2 |
| Baguelin et al (2010) [11] | Influenza A/H1N1 |  | UK (Unspecified) | EQ-5D-3L | Assessed together by children and parents. Postal survey | Unspecified |
| Balottin et al (2014) [12] | Migraine | Treatment: Psychotherapy  Control: CBT | Italy | EQ-5D-3L VAS | Self-assessed by children. Face-to-face interview | Mean: 10.2, 9.2; 6-18 |
| Banks et al (2008) [13] | Cancer patients: leukaemia, lymphoma, brain tumour | Intravenous chemotherapy | Canada (Unspecified) | HUI2, HUI3 | Proxy-assessed by parents. Self-administered paper questionnaire | Mean: 9.5; 2-18 |
| Barr et al (1993) [14] | Cancer survivors: ALL | St. Jude VIII protocol; Memorial Sloan L2 protocol; DFCI 85-01; DFCI 87-01 | Canada (Canada) | HUI2 | Proxy-assessed by nurses after physician consultation. Self-administered paper questionnaire | Unspecified |
| Barr et al (1994) [15] | Cancer survivors: brain tumours (PNET, medulloblastoma, astrocytoma, glioma, glioblastoma) | Surgery; Chemotherapy | Canada (Canada) | HUI2 | Proxy-assessed by physicians, nurses or parents. Self-administered paper questionnaire | Unspecified |
| Barr et al (1997) [16] | Cancer patients: ALL in remission | DFCI 87-01; DFCI 91-01 | Canada (Canada) | HUI2 | Proxy-assessed by physicians, nurses or parents. Self-administered paper questionnaire | Median: 3.9; 0.9-14 |
| Barr et al (1999) [17] | Cancer survivors: brain tumour (PNET, medulloblastoma, astrocytoma, glioma, ependymoma) | Surgery; Chemotherapy; Radiotherapy | Canada (Canada) | HUI2, HUI3 | Proxy-assessed by nurses. Self-administered paper questionnaire | Mean: 9.5; 1.7-17.9 |
| Barr et al (2000) [18] | Cancer survivors: Wilm’s tumour; SNS tumour (neuroblastoma) | Surgery; Chemotherapy; Radiotherapy; Stem cell transplantation | Canada (Canada) | HUI2, HUI3 | Proxy-assessed by parents. Postal survey | Mean: 9.1, 7.8; 2.8-20 |
| Barr et al (2001) [19] | Cancer survivors: ALL; NHL; Hodgkin’s disease; Wilm’s tumour; neuroblastoma; STS; retinoblastoma; AML | Unspecified cancer therapy | Colombia, Honduras, Uruguay, Cuba (Canada) | HUI2, HUI3 | Proxy-assessed by parents or physicians. Self-administered paper questionnaire or face-to-face interview | Unspecified |
| Barton et al (2006) [20] | Hearing loss | Cochlear implantation | UK (Canada) | HUI3 | Proxy-assessed by parents. Postal survey | Unspecified |
| Baumann et al (2016) [21] | Very low birthweight/very preterm birth; General health (normal birthweight/term) |  | Germany (Canada) | HUI3 | Self-assessed by children; Proxy-assessed by parents.  Postal survey | Mean: 13 |
| Beckman et al (2016) [22] | Victims of bullying; General health (control) |  | Sweden (UK) | SF-6D | Self-assessed by children. Postal survey | Mean: 16.2; 15-18 |
| Beerthuizen et al (2016) [23] | Asthma | Treatment 1: Web-based asthma management  Treatment 2: 4-monthly FENO-based asthma management  Control: Standard asthma management | Netherlands (Netherlands) | EQ-5D-3L, EQ-5D VAS | Assessed together by children and parents.  Self-administered paper questionnaire | Mean: 10.6;  4-18 |
| Belfort et al (2011) [24] | Overweight or obese; General health (normal weight) |  | USA (Canada) | HUI2, HUI3 | Self-assessed by children or assessed together by children and parents. Face-to-face interview | Mean: 10.3, 11.5; 5-18 |
| Bennett et al (2000) [25] | Bacterial meningitis; Permanent sequelae (brain damage, deafness) | Emergency department; Hospitalisation | USA | Chained Gamble | Proxy-assessed by parents. Online survey | 0.25-3 |
| Bergfors et al (2015) [26] | Asthma | Inhaled steroids | Sweden | EQ-5D-Y VAS | Assessed together by children and caregivers. Self-administered paper questionnaire | Mean: 12.1; 8-16 |
| Bilcke et al (2012) [27] | Chickenpox |  | Belgium (Unspecified) | EQ-5D-3L | Proxy-assessed by parents. Face-to-face interview | 0-3 |
| Bilcke et al (2017) [28] | Experience of severe disease; General health |  | Belgium (Belgium) | EQ-5D-3L; EQ-5D VAS | Proxy-assessed by parents.  Postal survey | 0-12 |
| Billson et al (1994) [29] | Cancer survivors: ALL; brain tumours; Wilm’s tumour; retinoblastoma; Hodgkin’s disease; NHL; AML; STS; neuroblastoma; Ewing’s sarcoma; ovarian sarcoma; osteogenic sarcoma | Unspecified cancer therapy | UK (Canada) | HUI2 | Proxy-assessed by physicians or assessed together by children and parents. Self-administered paper questionnaire | 2-17 |
| Bodden et al (2008) [30] | Anxiety disorder: social phobia; separation anxiety disorder; panic disorder | Individual CBT;  Family CBT | Netherlands (UK) | EQ-5D-3L VAS,  EQ-5D-3L | Assessed together by children and parents. Self-administered paper questionnaire. | Mean: 12.3; 8-17 |
| Bolton et al (2014) [31] | Overweight or obese; General health (normal weight) |  | Australia (Australia) | AQoL-6D | Self-assessed by children. Self-administered paper questionnaire. | Mean: 14.5; 11-19.6 |
| Bolton et al (2016) [32] | General health: students from rural or urban areas | Diet and physical activity | Australia (Australia) | AQoL-6D | Self-assessed by children. Self-administered paper questionnaire | Mean: 14.6; 11-19 |
| Boran et al (2011) [33] | Cancer patient: ALL; Ewing’s sarcoma; Hodgkin’s disease; NHL; STS; PNET; Wilm’s tumour; medulloblastoma; neuroblastoma; osteosarcoma; AML | Chemotherapy: neutropenia stage; post-neutropenia stage | Turkey (Canada) | HUI2, HUI3 | Proxy-assessed by parents. Face-to-face interview. | Mean: 7.72; 0.9-14 |
| Boulton et al (2006) [34] | Vision disorder: nystagmus; visual pathway impairment |  | UK (Canada) | HUI3 | Proxy-assessed by parents. Face-to-face interview. | Mean: 6.17; 3-8 |
| Bouwmans et al (2014) [35] | ADHD; Comorbidities (learning disorder, autistic disorder, anxiety disorder, mood disorder, asthma, tic disorder, epilepsy, mental retardation) | ADHD medication | Netherlands (Netherlands) | EQ-5D-3L | Proxy-assessed by parents. Online survey. | Mean: 11.9; 8-18 |
| Boyer et al (2014) [36] | General health | School-based intervention for improving social and emotional wellbeing | UK (Unspecified) | CHU9D | Self-assessed by children. Unspecified mode of administration. | 8-10 |
| Boyer et al (2016) [37] | General health | “Roots of Empathy”: School-based intervention for improving social and emotional wellbeing | UK (Australia) | CHU9D | Self-assessed by children. Self-administered paper questionnaire | 8-9 |
| Boyle et al (2010) [38] | Overweight or obese; General health (normal weight) | Physical activity | UK (UK) | EQ-5D-Y VAS,  EQ-5D-Y | Self-assessed by children. Postal survey. | Mean: 13.2; 11-15 |
| Braam et al (2016) [39] | Cancer: haematological malignancy; brain and CNS tumour; other solid cancer | Treatment: “Quality of Life in Motion” – 12-week physical exercise and psychosocial training  Control: usual care without physical training | Netherlands (Netherlands) | EQ-5D-Y | Self-assessed by children. Self-administered paper questionnaire; Postal survey | Mean: 13.4; 8-18 |
| Bradlyn et al (1993) [40] | Cancer: ALL; lymphoma; osteosarcoma; neuroblastoma | Surgery; Chemotherapy; Radiotherapy | USA (USA) | QWB | Proxy-assessed by parents. Face-to-face interview. | Mean: 10; 4.8-17.7 |
| Brisson et al (2010) [41] | Acute rotavirus gastroenteritis | Primary care | Canada (Canada) | EQ-5D-3L VAS,  HUI2 | Proxy-assessed by parents. Self-administered paper questionnaire. | Mean: 1.42; 0-3 |
| Brown et al (2009) [42] | Heart failure | Heart transplant; Intensive care (ECMO bridging) | UK (Canada) | HUI2 | Proxy-assessed by parents. Face-to-face interview. | 1-16 |
| Brunner et al (2003) [43] | MSKD: juvenile rheumatoid arthritis; systemic lupus erythematosus; juvenile dermatomyositis; arthropathy secondary to haemophilia |  | Canada (Canada) | SG, VAS (Linear analogue scale, Categorical scale),  HUI3 | Proxy-assessed by parents or self-assessed by children. Face-to-face interview. | 1-18 |
| Brunner et al (2004) [44] | MSKD: juvenile rheumatoid arthritis; Castleman syndrome; systemic lupus erythematosus; juvenile dermatomyositis; arthritis associated with IBD; juvenile psoriatic arthritis; juvenile ankylosing spondylitis; sarcoidosis |  | USA | VAS, SG (Lifelong arthritis as pit) | Proxy-assessed by parents or self-assessed by children. Face-to-face interview. | 3-18 |
| Brussoni et al (2013) [45] | Injury: head; upper extremity; lower extremity; dislocation; internal organ; facial; open wounds; burn; nerve; dental; poisoning; asphyxia | Emergency department; Hospitalisation | Canada (UK) | EQ-5D-3L VAS,  EQ-5D-3L | Assessed together by children and parents. Postal survey or non-postal Self-administered paper questionnaire. | 0-16 |
| Bull et al (2014) [46] | Cancer survivors: medulloblastoma (WNT molecular group, SHH group, non-WNT/SHH group) | Protocol at SIOP-UKCCSG PNET3 clinical trial | UK (Unspecified) | HUI3 | Proxy-assessed by parents or self-assessed by children. Unspecified mode of administration. | Median: 15.7, 14.5, 14.7; 10.3-18.2 |
| Burstrom et al (2011) [47] | Asthma; Diabetes; JIA; Celiac disease; General health (no disease) |  | Sweden | EQ-5D-Y VAS | Self-assessed by children. Face-to-face interview. | Mean: 9.87; 8-12 |
| Burstrom et al (2014) [48] | JIA; Arthrogryposis; Myelomeningocele; Cerebral palsy; Lower limb deformities; Achondroplasia |  | Sweden | EQ-5D-Y VAS | Self-assessed by children. Postal survey or Face-to-face interview. | Mean: 12, 13.3; 7-17 |
| Buysse et al (2008) [49] | Meningococcal sepsis; Permanent sequelae (severe mental retardation, neurological impairment, mild renal failure) | Survivors of intensive care | Netherlands (Canada) | VAS, HUI2, HUI3 | Proxy-assessed by parents. Face-to-face interview. | Median: 14.5; 5.3-31.1 |
| Byford et al (2007) [50] | Depression; Comorbidity (behavioural disorder) | Treatment: CBT and selective serotonin reuptake inhibitor (SSRI)  Control: SSRI alone | UK (UK) | EQ-5D-3L VAS,  EQ-5D-3L | Self-assessed by children. Face-to-face interview. | Median: 14; 11-17 |
| Byford et al (2013) [51] | Depression; Comorbidity (behavioural disorder) | Treatment: CBT and selective serotonin reuptake inhibitor (SSRI)  Control: SSRI alone | UK (UK) | EQ-5D-3L | Self-assessed by children. Face-to-face interview. | Median: 14; 11-17 |
| Canaway and Frew (2013) [52] | General health |  | UK (UK, Netherlands) | CHU9D,  EQ-5D-Y | Self-assessed by children. Face-to-face interview. | Mean: 6.37; 6-7 |
| Canaway and Frew (2014) [53] | Overweight or obese; General health (normal weight) |  | UK (UK, Netherlands) | CHU9D,  EQ-5D-Y | Self-assessed by children. Face-to-face interview. | 6-7 |
| Cardarelli et al (2006) [54] | Cancer survivors: brain tumour; leukaemia; lymphoma; solid tumour | Radiotherapy | Italy (Canada) | HUI2 | Proxy-assessed by parents or self-assessed by children. Self-administered paper questionnaire. | Mean: 13.3; 8-28 |
| Carroll and Downs (2009) [55] | ADHD; Allergy; Asthma; Cerebral palsy; Gastroenteritis; Hearing loss; Mental retardation; Otitis media; Seizure disorder; Vision disorder | Intensive care; Hospitalisation | USA | Chained Gamble,  TTO | Proxy-assessed by parents from general public. Face-to-face interview. | Unspecified |
| Chadha et al (2010) [56] | Recurrent respiratory papillomatosis | Surgery | Canada (Canada) | VAS, HUI3 | Assessed together by children and caregivers. Face-to-face interview. | Median: 9; 1.42-17 |
| Chen et al (2008) [57] | Acne | Hypothetical acne treatment | USA | TTO | Self-assessed by children. Self-administered paper questionnaire. | Mean: 15.4; 14-18 |
| Chen et al (2014) [58] | Overweight or obese; General health (normal weight) | Physical activity | Australia (Australia) | CHU9D | Self-assessed by children. Self-administered paper questionnaire. | Mean: 10.6, 15.1; 9-16 |
| Chen et al (2015) [59] | General health |  | Australia (Australia) | CHU9D,  EQ-5D-Y | Self-assessed by children. Online survey. | Mean: 14; 11-17 |
| Cheng et al (2000) [60] | Hearing loss | Cochlear implantation | USA (Canada) | VAS, TTO, HUI3 | Proxy-assessed by parents. Postal survey. | Mean: 7.5, 7.4, 10 |
| Cheng et al (2011) [61] | Cancer patients: leukaemia; lymphoma; solid tumour; brain tumour | Chemotherapy with hypothetical modes of administration | Canada (Unspecified) | VAS, HUI2, HUI3 | Proxy-assessed by parents or self-assessed by children. Face-to-face interview. | 0-18 |
| Cheung et al (2016) [62] | Adolescent idiopathic scoliosis | Observation with regular follow-up; Bracing; Surgery | China (China) | EQ-5D-5L | Self-assessed by children. Face-to-face interview | Mean: 15.6; 10-20 |
| Chevreul et al (2015) [63] | Cystic fibrosis |  | France (Denmark, England, Italy, Netherlands, Scotland, Poland) | EQ-5D-5L VAS,  EQ-5D-5L | Assessed together by children and caregivers.  Online survey. | Mean: 8.5 |
| Chevreul et al (2015b) [64] | Fragile X syndrome |  | France (Denmark, England, Italy, Netherlands, Scotland, Poland) | EQ-5D-5L | Assessed together by children and caregivers.  Online survey. | Mean: 10.3 |
| Chiou et al (2005) [65] | Asthma |  | USA (USA) | PAHOM | Self-assessed by children. Self-administered paper questionnaire. | Mean: 9.31 |
| Chirivella et al (2009) [66] | Cancer patients: ALL; STS; Hodgkin’s disease; NHL; Ewing’s sarcoma; osteosarcoma; Wilm’s tumour; AML; ovarian tumour; germ cell tumour | Chemotherapy; Radiotherapy | India (Canada) | HUI2, HUI3 | Proxy-assessed by physicians. Self-administered paper questionnaire. | 5-15 |
| Clement et al (2015) [67] | Unilateral slipped capital femoral epiphysis | Surgery: prophylactic fixation or unilateral fixation | UK (UK) | SF-6D | Self-assessed by children.  Self-administered paper questionnaire | Mean: 12.3;  9-16 |
| Covaciu et al (2013) [68] | Allergy; Asthma; Rhinitis; Eczema; Food hypersensitivity | Inhaled steroids | Sweden | EQ-5D-3L VAS | Proxy-assessed by parents. Self-administered paper questionnaire. | Mean: 8 |
| Cox et al (2005) [69] | Cancer patients: ALL | High-dose methotrexate therapy | USA (Unspecified) | HUI3 | Proxy-assessed by nurses. Self-administered paper questionnaire. | Median: 10; 6-18 |
| Creswell et al (2017) [70] | Anxiety disorder: Separation anxiety disorder; Social anxiety disorder; Generalised anxiety disorder; Specific phobia | Treatment: Brief guided parent-delivered cognitive behavioural therapy  Control: Solution-focused brief therapy | UK (UK, Unspecified) | CHU9D, EQ-5D-Y | Self-assessed by children; Proxy-assessed by caregivers. Self-administered paper questionnaire | Mean: 9.28; 5-12 |
| Cunha et al (2013) [71] | Admission to PICU: Elective postoperative; Respiratory; Sepsis; Trauma | Intensive care | Portugal (Canada) | HUI3 | Proxy-assessed by parents. Face-to-face or telephone interview. | 6-18 |
| Czyzewski et al (1994) [72] | Cystic fibrosis |  | USA (USA) | QWB | Proxy-assessed by parents or self-assessed by children. Face-to-face interview. | Mean: 8.74; 0.2-17.9 |
| Dakin et al (2010) [73] | Bilateral otitis media with effusion | Treatment: intranasal corticosteroids  Control: placebo | UK (Canada) | HUI2, HUI3 | Assessed together by children and parents. Self-administered paper questionnaire. | Mean: 6.39; 4-7 |
| de Bruin et al (2016) [74] | Insomnia | Treatment: Internet cognitive behavioural therapy (6 weeks)  Control: Face-to-face group cognitive behavioural therapy (6 weeks) | Netherlands (Netherlands) | EQ-5D-3L | Self-assessed by children. Online survey; Self-administered paper questionnaire | Mean: 15.4; 12-19 |
| de Jong et al (2012) [75] | Craniosynostosis:  Apert syndrome;  Crouzon syndrome;  Muenke syndrome;  Saethre-Chotzen syndrome |  | Netherlands (Canada) | VAS, HUI3 | Proxy-assessed by parents. Self-administered paper questionnaire. | Mean: 9.6; 4-18 |
| de Keizer et al (1997) [76] | Admission to PICU | Intensive care: cardiovascular and other surgery; non-surgical treatments | Netherlands (Canada) | HUI2 | Proxy-assessed by parents. Postal survey or non-postal Self-administered paper questionnaire | 1-16 |
| de Kinderen et al (2016) [77] | Epilepsy | Treatment: ketogenic diet  Control: usual care | Netherlands (Netherlands) | EQ-5D-Y | Self-assessed by children. Self-administered paper questionnaire. | Mean: 7.8, 8.1; 1.1-15.7 |
| de Lissovoy et al (2007) [78] | Cerebral palsy | Hypothetical intrathecal baclofen therapy | USA (Canada) | HUI2 | Proxy-assessed by physicians. Self-administered paper questionnaire. | Mean: 11 |
| de Sonneville-Koedoot et al (2015) [79] | Stuttering | Treatment: DCM therapy  Control: Lidcombe programme | Netherlands (Canada) | EQ-5D-3L VAS,  HUI3 | Proxy-assessed by parents. Self-administered paper questionnaire. | Mean: 4.51, 4.29; 3-6 |
| de Wolf et al (2011) [80] | Hearing loss: unilateral; bilateral | Bone-anchored hearing aid | Netherlands (Canada) | HUI3 | Assessed together by children and parents. Self-administered paper questionnaire. | Median: 4,7; 11-16 |
| Diarbakerli et al (2017) [81] | General health |  | Sweden (UK, Sweden) | EQ-5D-3L | Self-assessed by children. Postal survey | Mean: 14.5; 10-19 |
| Dillman et al (2016) [82] | Small bowel Crohn’s disease | MRI; Infliximab medical therapy | USA | VAS, TTO, SG | Assessed together by children and parents. Face-to-face interview | Mean: 15.2; 9-18 |
| Domellof et al (2014) [83] | ASD; Movement disorder; Learning disability; Hearing loss |  | Sweden | EQ-5D-Y VAS | Assessed together by children and parents. Postal survey. | Mean: 12, 11, 11.5, 12.3; 7-17 |
| Domino et al (2008) [84] | Depression | CBT alone; Fluoxetine alone; CBT and fluoxetine; Placebo | USA (USA) | Utility mapping from depression-free days | Self-assessed by children. Self-administered paper questionnaire. | Mean: 14.6; 12-18 |
| Domino et al (2009) [85] | Depression | CBT alone; Fluoxetine alone; CBT and fluoxetine; Placebo | USA (USA) | Utility mapping from depression-free days and from PQ-LES-Q | Self-assessed by children. Self-administered paper questionnaire. | Mean: 14.7; 12-17 |
| Duckworth et al (2015) [86] | Cancer survivors: brain tumours (PNET, medulloblastoma, ependymoma; germ cell tumour; astroglial tumour) | Surgery; Chemotherapy | Canada (Canada) | HUI2, HUI3 | Proxy-assessed by parents or self-assessed by children. Face-to-face interview. | Mean: 16.7 |
| Ebrahim and Parshuram (2015) [87] | Admission to PICU: Respiratory; Neurological; Circulatory; Oncological | Intensive care | Canada (Canada) | VAS, HUI3 | Proxy-assessed by parents. Self-administered paper questionnaire. | Mean: 10.3; 4-18 |
| Eidt-Koch et al (2009) [88] | Cystic fibrosis | Hospitalisation | Germany | EQ-5D-Y VAS | Self-assessed by children. Self-administered paper questionnaire. | Mean: 10.8, 15.9; 8-17 |
| Ekert et al (2001) [89] | Haemophilia | Hypothetical on-demand usual care; Hypothetical NovoSeven therapy | Australia (Unspecified) | EQ-5D-3L | Self-assessed by children. Face-to-face interview. | Mean: 14; 11-16 |
| Engelmann et al (2015) [90] | IBD: Crohn’s disease; Ulcerative colitis | IBD treatment and psychotherapy | Germany | EQ-5D-3L VAS | Assessed together by children and parents. Face-to-face interview. | Mean: 15.2; 10-18 |
| Epps et al (2005) [91] | JIA | Treatment: Hydrotherapy and land-based therapy  Control: Land-based therapy | UK (UK) | EQ-5D-3L | Proxy-assessed by parents. Self-administered paper questionnaire. | Mean: 11,12; 4-19 |
| Ersberg and Gerdhem (2013) [92] | Congenital scoliosis; Idiopathic scoliosis; Neuromuscular scoliosis | Posterior/anterior surgery for spinal deformity | Sweden (UK) | EQ-5D-3L | Self-assessed by children. Postal survey. | Mean: 14.6, 14.7, 15.7; 9-20 |
| Fantaguzzi et al (2017) [93] | Victims of bullying | “Learning Together”: Intervention to reduce aggression and bullying | UK (UK, Australia) | CHU9D | Self-assessed by children. Self-administered paper questionnaire | Mean: 11.75; 11-12 |
| Feenstra et al (2012) [94] | Personality disorder; Substance use disorder; Somatoform disorder (conversion disorder, hypochondriasis); Eating disorder (anorexia nervosa, bulimia nervosa); Anxiety disorder (social phobia, panic disorder, agoraphobia, obsessive compulsive disorder, PTSD); Enuresis; Mood disorder (dysthymic disorder, major depressive disorder | Hospitalisation; Psychotherapy; Psychosocial nursing; Nonverbal therapies | Netherlands (Netherlands) | EQ-5D-3L | Self-assessed by children. Face-to-face interview. | Mean: 16.6; 14-19 |
| Feeny et al (2004) [95] | Extremely low birthweight; Extremely preterm birth; Comorbidities (neurosensory impairment, cerebral palsy, hydrocephalus, cognitive impairment, autism, unilateral/bilateral blindness, deafness); General health (control) |  | Canada (Canada) | SG (Pit: worst health), HUI2, HUI3 | Self-assessed by children. Face-to-face interview. | Mean: 14, 14.2, 14.4; 12-16 |
| Felder-Puig et al (2000) [96] | Cancer survivors: ALL; NHL; Hodgkin’s disease; AML; chronic myeloid leukaemia; osteosarcoma; Wilm’s tumour; Ewing’s sarcoma; immunodeficiency syndrome; STS; neuroblastoma | Unspecified cancer therapy | Austria (Canada) | HUI2 | Proxy-assessed by physicians or nurses or assessed together by children and parents. Self-administered paper questionnaire. | Median: 13; 6-30 |
| Fluchel et al (2008) [97] | Cancer survivors: ALL; brain tumour; Wilm’s tumour; retinoblastoma; Hodgkin’s disease; NHL; AML; STS; neuroblastoma; Ewing’s sarcoma; ovarian sarcoma; osteogenic sarcoma; General health (control) | Surgery; Chemotherapy; Radiotherapy | Uruguay (Canada) | HUI3 | Self-assessed by children. Face-to-face interview. | Mean: 13.6; 7-27 |
| Foreman et al (1999) [98] | Cancer survivors: medulloblastoma; ependymoma; astrocytoma; craniopharyngioma; ganglioglioma; germinoma; pituitary tumour | Surgery; Chemotherapy; Radiotherapy; Shunt | USA (Canada) | HUI2 | Proxy-assessed by parents. Telephone interview. | Mean: 16.4; 12.3-20.3 |
| Foster Page et al (2015) [99] | Dental caries | Resin infiltration; Fluoride varnish | New Zealand (UK) | CHU9D | Self-assessed by children. Face-to-face interview. | Mean: 8.3; 6.6-9.9 |
| Frew et al (2015) [100] | Overweight or obese; General health (normal weight) |  | UK (UK) | CHU9D | Self-assessed by children. Face-to-face interview. | Mean: 6.3; 6-7 |
| Friedman et al (2004) [101] | Atopic dermatitis | Visual aid | USA | VAS | Proxy-assessed by parents. Online survey. | 0.25-18 |
| Fu et al (2006) [102] | Cancer survivors: ALL; leukaemia; lymphoma; renal tumour; germ cell tumour; retinoblastoma; bone tumour; CNS tumour; STS; carcinoma | Surgery; Chemotherapy; Radiotherapy | El Salvador, Honduras, Nicaragua, Panama (Canada) | HUI2, HUI3 | Proxy-assessed by parents or physicians or self-assessed by children. Face-to-face interview or Self-administered paper questionnaire. | Median: 12.8; 3.4-25.8 |
| Furber and Segal (2015) [103] | Mental disorder | Child and adolescent mental health services (CAMHS) | Australia (UK, Australia) | CHU9D | Proxy-assessed by caregivers. Telephone interview. | Mean: 11.71; 5-17 |
| Furlong et al (2012) [104] | Cancer patients: ALL  Cancer survivors: ALL | DFCI 95-01; DFCI 95-05 | USA, Canada (Canada) | HUI2, HUI3 | Proxy-assessed by parents. Self-administered paper questionnaire. | Mean: 9.8; 5-20.8 |
| Gaitan-Lopez et al (2017) [105] | General health |  | Colombia | EQ-5D-Y VAS | Self-administered paper questionnaire | Mean: 12.7; 9-17 |
| Garcia-Gordillo et al (2016) [106] | General health |  | Spain (Spain) | EQ-5D-5L, EQ-5D VAS | Self-assessed by children. Self-administered paper questionnaire | 15-17 |
| Geneid et al (2011) [107] | Laryngotracheal stenosis; Comorbidities (asthma, language disorder, stuttering, ventricular septal defect, Down syndrome) | Early infancy laryngotracheal reconstruction | Finland (Finland) | 16D, 17D | Proxy-assessed by parents or self-assessed by children. Postal survey. | 0-15.9 |
| Gerald et al (2012) [108] | Asthma | Outpatient care | USA (USA) | PAHOM | Proxy-assessed by parents or self-assessed by children. Face-to-face interview. | 6-12 |
| Glaser et al (1999) [109] | Cancer survivors: brain tumour (PNET, astrocytoma, medulloblastoma, ependymoma) | Chemotherapy; Radiotherapy | UK (Canada) | HUI2, HUI3 | Proxy-assessed by physiotherapist, parents or physicians or self-assessed by children. Self-administered paper questionnaire. | Mean: 10.5; 6-16 |
| Glotzer et al (1995) [110] | Lead poisoning; Sequelae (reading disability) |  | USA | VAS | Proxy-assessed by physicians. Self-administered paper questionnaire. | 0-2 |
| Goorden et al (2016) [111] | Cannabis use disorder | Treatment: Multidimensional family therapy  Control: Individual cognitive behavioural therapy | Netherlands (Netherlands) | EQ-5D-3L | Self-assessed by children. Face-to-face interview | Mean: 16.7; 13-18 |
| Gospodarevskaya (2013) [112] | PTSD; Comorbidity (depression) |  | Australia (Unspecified) | AQoL-5D | Self-assessed by children. Self-administered paper questionnaire. | Mean: 18.3; 16-21 |
| Grano et al (2013) [113] | Risk of psychosis | Community- and family-orientated early mental health intervention | Finland (Finland) | 16D | Self-assessed by children. Telephone interview. | Mean: 15.3; 12-21 |
| Grano et al (2014) [114] | Risk of psychosis | Community- and family-orientated early mental health intervention | Finland (Finland) | 16D | Self-assessed by children. Telephone interview. | Mean: 15.1; 12-21 |
| Grant et al (2006) [115] | Cancer survivors: leukaemia; Hodgkin’s disease; Burkitt’s lymphoma; ependymoma; astrocytoma; medulloblastoma; neuroblastoma; Wilm’s tumour; bone tumour; STS; gonadal neoplasm | Surgery; Chemotherapy; Radiotherapy | Canada (Canada) | HUI2, HUI3 | Self-assessed by children. Postal survey. | 14.6-20.3 |
| Gray et al (2007) [116] | Extremely low birthweight; Extremely preterm birth; General health (normal term birth) |  | UK (Canada) | HUI3 | Self-assessed by children. Postal survey. | 15-16 |
| Greenough et al (2014) [117] | Extremely low birthweight; Extremely preterm birth; Admission to PICU | Intensive care; Treatment: HFO ventilation  Control: usual ventilation | UK, Ireland, Australia, Singapore (Unspecified) | HUI3 | Proxy-assessed by parents or self-assessed by children. Postal survey. | Mean: 12.5, 12.6; 11.2-14.4 |
| Gulati et al (2017) [118] | Lumbar disc herniation; Sciatica | Single-level lumbar microdiscectomy | Norway (Unspecified) | EQ-5D-3L | Self-assessed by children. Self-administered paper questionnaire | Mean: 17.5; 13-19 |
| Gundle et al (2017) [119] | Bone tumour: Osteosarcoma; Ewing’s sarcoma; Adamantinoma; Undifferentiated pleomorphic sarcoma | Limb salvage surgery | USA (Unspecified) | SF-6D | Self-assessed by children. Self-administered paper questionnaire | Mean: 16 |
| Haapamaki et al (2011) [120] | IBD: Crohn’s disease; Ulcerative colitis; Comorbidities (primary sclerosing cholangitis, asthma, allergy) | IBD treatment: aminosalicylic acid; azathioprine; corticosteroids | Finland (Finland) | 15D, 16D, 17D | Self-assessed by children or assessed together by children and parents. Postal survey. | Mean: 13.8; 7-19 |
| Haavisto et al (2013) [121] | Heart failure; Kidney failure; Liver failure | Kidney transplant; Liver transplant; Awaiting transplant | Finland (Finland) | 16D, 17D | Self-assessed by children. Self-administered paper questionnaire. | Mean: 11.3; 6.3-16.7 |
| Hailer et al (2014) [122] | Legg-Calve-Perthes disease |  | Sweden | EQ-5D-3L VAS | Self-assessed by children. Face-to-face interview. | 15-17 |
| Hakkaart-van Roijen et al (2011) [123] | Dyslexia; General health (no dyslexia) |  | Netherlands (Netherlands) | EQ-5D-3L | Proxy-assessed by physicians. Online survey. | Unspecified |
| Han et al (2011) [124] | Injury; Post-injury depression | Intensive care | USA (USA) | QWB | Self-assessed by children. Telephone interview. | Mean: 15; 12-19 |
| Hanberger et al (2009) [125] | Diabetes type I; Severe hypoglycaemia | Multiple insulin therapy | Sweden (Unspecified) | EQ-5D-3L | Proxy-assessed by parents or self-assessed by children. Postal survey. | 2.6-19.6 |
| Hartman et al (2016) [126] | Foetus affected by maternal use of tobacco |  | USA | Utility from Behaviour Problems Index | Proxy-assessed by parents. Postal survey | 4-14 |
| Hatam et al (2013) [127] | Phenylketonuria; Hypothyroidism; Galactosemia; Favism | Neonatal screening and treatment | Iran | TTO | Proxy-assessed by nurses. Face-to-face interview. | 0-2 |
| Hernandez et al (2016) [128] | Asthma |  | Unspecified (France) | EQ-5D-Y | Self-assessed by children. Online survey | 6-11 |
| Hinds et al (2007) [129] | Cancer patients: ALL | St. Jude Total 15 protocol | USA (Canada) | HUI3 | Proxy-assessed by nurses. Self-administered paper questionnaire. | Median: 8.6; 4.9-18.8 |
| Hodgkins et al (2013) [130] | ADHD | Treatment: stimulant lisdexamfetamine dimesylate  Control: osmotic-release oral system methylphenidate  Placebo | Unspecified (Unspecified) | HUI2 | Proxy-assessed by parents. Unspecified mode of administration. | 6-17 |
| Hoffmann et al (2011) [131] | Gastroenteritis; Rotavirus gastroenteritis | Hospitalisation; Primary care | Denmark (Denmark) | EQ-5D-3L VAS,  EQ-5D-3L | Proxy-assessed by parents. Self-administered paper questionnaire. | Median: 1.2; 0-4.7 |
| Hogan et al (2017) [132] | Chronic pain; Comorbidities (depression, anxiety, sleep problems, arthritis, back or neck problems, neuropathic pain, migraine, fibromyalgia, abdominal pain) |  | Canada (Canada) | HUI3 | Self-assessed by children. Postal survey | 12-17 |
| Holbrook et al (2005) [133] | Injury; Post-injury acute stress disorder | Intensive care; Hospitalisation | USA (USA) | QWB | Self-assessed by children. Telephone interview. | Mean: 15; 12-19 |
| Holbrook et al (2007) [134] | Injury; General health (control) | Intensive care; Hospitalisation | USA (USA) | QWB | Self-assessed by children. Face-to-face or telephone interview. | Mean: 15; 12-19 |
| Horsman et al (2008) [135] | Cancer survivors: germ cell tumour | Unspecified cancer therapy | Brazil (Unspecified) | HUI3 | Self-assessed by children. Self-administered paper questionnaire. | 13-18 |
| Huppertz et al (2008) [136] | Gastroenteritis (diarrhoea); Comorbidities (preterm birth, chronic lung disease, immunodeficiency, heart disease, atopic dermatitis) | Hospitalisation; Outpatient care | Germany | VAS | Proxy-assessed by parents. Self-administered paper questionnaire. | 0-2 |
| Iskrov et al (2015) [137] | Cystic fibrosis |  | Bulgaria (Unspecified) | EQ-5D-3L VAS,  EQ-5D-3L | Self-assessed by children. Online survey. | Mean: 16 |
| Janse et al (2005) [138] | ALL; Asthma; Cystic fibrosis; JIA |  | Netherlands (Canada) | HUI3 | Proxy-assessed by parents or physicians. Face-to-face or Self-administered paper questionnaire. | Mean: 8.5; 1.2-16.9 |
| Janssens et al (2009) [139] | Major external injury | Intensive care; Hospitalisation | Netherlands | EQ-5D-3L VAS | Assessed together by children and parents. Postal survey. | Mean: 15.1; 8-23 |
| Jelsma (2010) [140] | Allergy; Asthma; Eczema; MSKD; Headache; Diabetes; Depression; Heart disease; ADHD; General health (no disease) |  | South Africa | EQ-5D-3L VAS,  EQ-5D-Y VAS | Self-assessed by children. Self-administered paper questionnaire. | Mean: 15.5; 13-19.17 |
| Jelsma and Ramma (2010) [141] | Chronic illness; General health (no disease) |  | South Africa | EQ-5D-Y VAS | Proxy-assessed by parents or self-assessed by children. Self-administered paper questionnaire. | 7-12 |
| Jog et al (2016) [142] | Cerebral palsy | Onabotulinumtoxin A injection | Canada (Unspecified) | SF-6D | Self-assessed by children. Self-administered paper questionnaire | Mean: 16 |
| Joosten et al (2015) [143] | Chronic illness; Malnutrition | Steroids; Antibiotics; Diuretics | Netherlands | EQ-5D-3L VAS | Proxy-assessed by nurses. Self-administered paper questionnaire. | Median: 9.8; 4.2-13.4 |
| Juniper et al (1997) [144] | Asthma | Outpatient care | Canada (Canada) | VAS, SG,  10-dimensional HUI | Self-assessed by children. Face-to-face interview. | Mean: 12; 7-17 |
| Kang (2016) [145] | General health |  | South Korea (Unspecified) | CH-6D | Self-assessed by children. Online survey | 16-18 |
| Keating et al (2011) [146] | Overweight or obese; General health (normal weight) |  | Australia (Australia) | AQoL-6D | Self-assessed by children. Self-administered paper questionnaire. | Mean: 14.6; 11-18 |
| Kennedy and Leyland (1999) [147] | Cancer survivors: brain tumour (PNET, astrocytoma, glioma, medulloblastoma, ependymoma, germinoma, craniopharyngioma, ganglioneuroblastoma, ganglioglioma, choroid plexus papilloma) | Surgery; Chemotherapy; Radiotherapy; Shunt | UK (Canada) | HUI2 | Assessed together by children and parents. Face-to-face interview. | Mean: 8.6; 2.1-19.5 |
| Kennedy et al (2017) [148] | Bacterial meningitis (worst day of illness, after recovery); Long-term complications (hearing loss, fits/seizures, concentration and memory loss, amputations) | Inpatient tertiary care | UK (UK) | EQ-5D-Y, EQ-5D-Y VAS | Assessed together by children and parents.  Postal survey | 0-16 |
| Kesztyus et al (2014) [149] | Overweight or obese; General health (normal weight) | Physical activity | Germany | EQ-5D-Y VAS | Proxy-assessed by parents. Self-administered paper questionnaire. | Mean: 7.1; 5.4-9.8 |
| Kim et al (2017) [150] | General health |  | South Korea | EQ-5D-Y VAS | Assessed together by children and parents. Postal survey | Mean: 9.5;  7-13 |
| Klaassen et al (2010) [151] | Cancer survivors: Hodgkin’s disease | Chemotherapy: POG 9425, POG 9426, AHOD 0031, GPOH-HD95;  Radiotherapy | Canada (Canada) | EQ-5D-3L VAS, HUI2, HUI3 | Self-assessed by children. Self-administered paper questionnaire. | Mean: 14.7; 8.9-18 |
| Knapp et al (2012) [152] | Life-threatening illness requiring palliative care | Palliative care | USA (Canada) | HUI2, HUI3 | Proxy-assessed by parents. Telephone interview. | Mean: 12.4; 5-21 |
| Kodra et al (2014) [153] | Haemophilia A/B |  | Italy (Spain) | EQ-5D-3L VAS,  EQ-5D-3L | Proxy-assessed by caregivers. Online survey. | Mean: 8; 2-17 |
| Koomen et al (2005) [154] | Non-haemophilus influenza type B bacterial meningitis; Permanent sequelae (learning or behavioural limitations) |  | Netherlands (Canada) | HUI2 | Proxy-assessed by parents. Self-administered paper questionnaire. | Mean: 9.7; 5.3-14.2 |
| Kotwicki et al (2001) [155] | Cystic fibrosis; Comorbidities (nasal polyposis, asthma, sinusitis) | Vitamin; Pancreatic enzymes; Oral antibiotics; Bronchodilator; Corticosteroids; Oral antiacids; Ibuprofen | USA (USA) | QWB | Proxy-assessed by parents. Face-to-face interview. | Mean: 7.7; 5-11 |
| Kramer et al (1994) [156] | Viral fever; Permanent sequelae (unilateral or bilateral hearing loss, hypertension, pneumonia, bacterial meningitis, viral meningitis, neurodevelopmental impairment, urinary tract infection) | Hypothetical screening methods for viral fever | Canada | VAS | Proxy-assessed by parents, physicians or parents from general public. Face-to-face interview. | 0.25-2 |
| Kulkarni et al (2008) [157] | Hydrocephalus | Surgery; Hospitalisation | Canada (Canada) | HUI3 | Proxy-assessed by parents. Self-administered paper questionnaire. | Mean: 14.1; 6-19 |
| Kulkarni et al (2008b) [158] | Hydrocephalus | Cerebrospinal fluid shunt | Canada (Canada) | HUI3 | Proxy-assessed by parents. Self-administered paper questionnaire. | Mean: 11.6; 5-18 |
| Kulkarni et al (2010) [159] | Hydrocephalus | Endoscopic third ventriculostomy; Cerebrospinal fluid shunt | Canada (Canada) | HUI3 | Proxy-assessed by parents. Self-administered paper questionnaire. | Mean: 10.4, 11.9; 5-18 |
| Kulkarni et al (2010b) [160] | Obstructive hydrocephalus | Endoscopic third ventriculostomy; Cerebrospinal fluid shunt | Canada (Canada) | HUI3 | Proxy-assessed by parents. Self-administered paper questionnaire. | Mean: 12.3, 12; 5-18 |
| Kulpeng et al (2013) [161] | Severe bacteraemia; Acute otitis media; Severe infection; Permanent sequelae to severe infection (epilepsy, hearing loss, pneumonia, lung disease, severe meningitis, mental retardation) | Hospitalisation | Thailand (Thailand, Canada) | EQ-5D-3L VAS, EQ-5D-3L, HUI2, HUI3 | Proxy-assessed by caregivers or assessed together by children and caregivers. Face-to-face interview. | Mean: 10; 5-14 |
| Ladner et al (2016) [162] | Chiari type I malformation |  | USA (Canada) | HUI3 | Assessed together by children and parents. Face-to-face interview. | Mean: 12; 5-17 |
| Laitakari et al (2015) [163] | Burns | Hospitalisation; Outpatient care | Finland (Finland) | 17D | Assessed together by children and parents. Postal survey. | Mean: 7 |
| Landfeldt et al (2015) [164] | Duchenne muscular dystrophy | Intensive care: ventilation support | Germany, Italy, UK, USA (Canada) | HUI3 | Proxy-assessed by caregivers. Online survey. | Mean: 14 |
| Lee et al (2005) [165] | Pertussis; Pneumonia | Vaccination | USA | Short-term TTO (8-week health state), Long-term TTO (lifetime health state), 0%/3% discount rate | Proxy-assessed by parents. Telephone interview. | 11-17 |
| Lee et al (2011) [166] | Diabetes type I; Complications (blindness, end-stage renal disease, angina, stroke, amputation) | Intensive insulin therapy | USA (Canada) | TTO, HUI3 | Proxy-assessed by parents or self-assessed by children. Face-to-face interview. | Mean: 13.7; 8-18 |
| Lee et al (2013) [167] | Epilepsy | Intensive care; Hospitalisation | UK (Unspecified) | EQ-5D-3L | Proxy-assessed by physicians. Delphi process. | 0.5-18 |
| Lenhard et al (2017) [168] | Obsessive compulsive disorder | Treatment: Internet-delivered CBT  Control: Usual care | Sweden (Sweden) | EQ-5D-Y, EQ-5D-Y VAS | Self-assessed by children. Online survey | Mean: 14.21; 12-17 |
| Lin et al (2016) [169] | Diabetes Type I |  | China (Japan) | EQ-5D-3L | Proxy-assessed by parents; Self-assessed by children. Face-to-face interview | 3-13; 13-20 |
| Liu et al (2016) [170] | Profound bilateral sensorineural hearing loss; Severe-profound deafness | Unilateral cochlear implant | China | HUI3 | Assessed together by children and parents. Postal survey | 4-11 |
| Lloyd et al (2010) [171] | Diabetes type I | Hypothetical treatments: insulin injection, infusion therapy | UK (UK) | SG, EQ-5D-3L VAS,  EQ-5D-3L | Proxy-assessed by parents or general public. Face-to-face interview. | Mean: 9.95, 15.13; 8-17 |
| Lloyd et al (2011) [172] | ADHD |  | UK | TTO, VAS | Proxy-assessed by general public. Face-to-face interview. | Mean: 11; 8-16 |
| Lloyd et al (2015) [173] | Hypophosphatasia | Intensive care: invasive ventilation | UK (UK) | EQ-5D-3L | Proxy-assessed by parents or physicians. Face-to-face interview | 0-18 |
| Lock et al (2010) [174] | Tonsillitis | Tonsillectomy | UK | Chained Gamble | Proxy-assessed by parents or self-assessed by children. Face-to-face interview. | 12-18 |
| Lovett et al (2010) [175] | Hearing loss | Cochlear implantation: unilateral/bilateral | UK (Canada) | VAS, HUI3 | Proxy-assessed by parents. Self-administered paper questionnaire. | Mean: 7.2; 1.5-16 |
| Lynch et al (2005) [176] | Depression | Treatment: CBT  Control: usual care | USA (USA) | Utility mapping from depression-free days | Proxy-assessed by physicians. Face-to-face interview. | Mean: 14.4; 13-18 |
| Lynch et al (2016) [177] | Moderate/severe depression; Subthreshold depression; No depression (general health) |  | USA (USA, Canada) | EQ-5D-3L, HUI2, HUI3, QWB,  SF-6D | Self-assessed by children. Telephone interview | Mean: 15.4; 13-17 |
| Maia et al (2016) [178] | ADHD; Comorbidities (oppositional defiant disorder, conduct disorder, anxiety disorder, mood disorder) | Immediate-release methylphenidate | Brazil (Canada) | HUI3 | Proxy-assessed by parents. Self-administered paper questionnaire. | Mean: 8.62, 13.67; 6-17 |
| Makkes et al (2013) [179] | Overweight or obese; Comorbidities (acanthosis nigricans, Blount’s disease, gallstones, hirsutism, pseudo-gynecomastia) |  | Netherlands (Netherlands) | EQ-5D-3L VAS,  EQ-5D-3L | Self-assessed by children. Self-administered paper questionnaire. | Mean: 14.8; 8-19 |
| Malik et al (2013) [180] | IBD: Ulcerative colitis | IBD treatment: usual therapy; proctocolectomy | Canada | EQ-5D-3L VAS | Proxy-assessed by parents. Postal survey. | Mean: 14.6, 13.4 |
| Marlow et al (2015) [181] | Acute rotavirus gastroenteritis | Emergency department; Hospitalisation | UK (UK) | VAS, HUI2 | Proxy-assessed by parents. Self-administered paper questionnaire. | Median: 1.16; 0-6 |
| Martin et al (2008) [182] | Acute rotavirus gastroenteritis | Hospitalisation; Primary care | UK (UK) | Adjusted EQ-5D-3L (Mobility and/or self-care domain set at 1), EQ-5D-3L | Proxy-assessed by physicians. Self-administered paper questionnaire. | 0-5 |
| Matza et al (2005) [183] | ADHD | Hospitalisation; Stimulant therapy; Non-stimulant therapy; Psychotherapy | UK, USA (UK) | VAS,  EQ-5D-3L | Proxy-assessed by parents. Face-to-face interview. | Mean: 10.2, 11.8; 7-18 |
| Matza et al (2005b) [184] | ADHD | Stimulant therapy; Non-stimulant therapy | USA | SG (Pit: death), SG (Pit: worst health), VAS | Proxy-assessed by parents. Face-to-face interview. | Mean: 10.2; 7-18 |
| McBain et al (2016) [185] | Post-traumatic stress disorder in war-affected youths | Treatment: “Youth Readiness Intervention” – group-based therapy  Control: Family and community support | Sierra Leon | Utility from WHO Disability Adjustment Scale II | Self-assessed by children. Face-to-face interview | Mean: 17.8; 15-24 |
| Midgley et al (2000) [186] | Liver disease: biliary atresia; biliary cirrhosis; alpha-antitrypsin deficiency; metabolic liver disease; fulminant hepatic failure; neonatal hepatitis | Liver transplant | USA (Canada) | HUI2 | Proxy-assessed by parents. Telephone interview. | Median: 4.94; 2-18 |
| Miller et al (2013) [187] | Risk of HIV infection | School-based prosocial care to reduce early marriage | Zimbabwe (Zimbabwe, Thailand) | EQ-5D-3L | Self-assessed by children. Face-to-face interview. | Unspecified |
| Miller et al (2014) [188] | Abusive head trauma | Intensive care: eye surgery; brain shunt | USA (Canada) | HUI2 | Proxy-assessed by physicians and caregivers.  Online survey. | Unspecified |
| Mittmann et al (1999) [189] | Acne; Allergy; Asthma; Back pain; Cardiovascular disease; Cancer survivors; Diabetes; Epilepsy; Gastric ulcer; Hypertension; JIA; Lung disease; Migraine; Sinusitis; Stroke; Urinary incontinence; Cataracts; General health (no disease) |  | Canada (Canada) | HUI3 | Self-assessed by children. Telephone interview. | 12-19 |
| Mort et al (2011) [190] | Cancer survivors: ALL; NHL; Hodgkin’s disease; neuroblastoma; Wilm’s tumour; gonadal tumour; osteosarcoma; STS; retinoblastoma | Surgery; Chemotherapy; Radiotherapy; Stem cell transplantation | Finland (Finland) | 16D, 17D | Self-assessed by children. Postal survey. | Mean: 14.4; 12-18 |
| Mpundu-Kaambwa et al (2017) [191] | General health |  | Australia (Australia) | CHU9D | Self-assessed by children. Online survey | Mean: 15.8; 15-17 |
| Mulhern (1999) [192] | Cancer survivors: medulloblastoma | Surgery; Radiotherapy | USA (Unspecified) | HUI2 | Proxy-assessed by physicians. Face-to-face interview. | 3-21 |
| Munzenberger et al (1999) [193] | Cystic fibrosis: pulmonary exacerbation | Hospitalisation; Intravenous tobramycin and ceftazidime | USA (USA) | QWB | Assessed together by children and parents. Face-to-face interview. | Mean: 14; 9-20 |
| Murillo et al (2017) [194] | Diabetes Type I | Insulin therapy: multiple daily injections or pump therapy | Spain | EQ-5D-Y VAS | Assessed together by children and parents. Online survey | 8-19 |
| Naraine et al (2002) [195] | Haemophilia | On-demand haemophilia treatment; Low-, medium-, high-dose prophylaxis | Canada | SG | Proxy-assessed by parents, adult patients or general public. Face-to-face interview. | Unspecified |
| Neufeld et al (2012) [196] | Haemophilia | Recombinant activated factor VII; Delivery type: on-demand, induction prophylaxis | USA (USA) | EQ-5D-3L VAS,  EQ-5D-3L | Proxy-assessed by caregivers. Self-administered paper questionnaire. | Unspecified |
| Nokso-Koivisto et al (2014) [197] | Tonsillitis | Tonsillectomy | Finland (Finland) | 16D, 17D | Self-assessed by children. Postal survey. | Mean: 9.4, 13.9; 7-15 |
| Nordyke et al (2011) [198] | Celiac disease; General health (control) | Intestine biopsy screening | Sweden | EQ-5D-Y VAS | Proxy-assessed by parents or Self-assessed by children. Postal survey or Self-administered paper questionnaire. | Mean: 12 |
| Nordyke et al (2013) [199] | Celiac disease; General health (control) | Intestine biopsy screening; Gluten diet | Sweden | EQ-5D-Y VAS | Self-assessed by children. Self-administered paper questionnaire. | Median: 13.2, 14.6 |
| Nwachukwu et al (2017) [200] | Patella dislocation (stable return or recurrent dislocation); General health |  | USA | VAS | Self-assessed by children. Telephone interview | Unspecified |
| Oh et al (1996) [201] | Acute otitis media; Adverse event (gastrointestinal, dermatological, serum sickness-like reaction) |  | Canada | VAS | Proxy-assessed by physicians. Postal survey. | 0.5-18 |
| Oostenbrink et al (2002) [202] | Bacterial meningitis; Permanent sequelae (hearing loss, epilepsy, mental retardation, severe retardation and tetraplegia, leg paresis, deafness) |  | Netherlands (UK, Canada) | EQ-5D-3L, HUI2, HUI3 (0: Worst health), HUI3 (0: Death) | Proxy-assessed by physicians. Self-administered paper questionnaire. | Mean: 6 |
| Orenstein et al (1990) [203] | Cystic fibrosis: pulmonary exacerbation | Antibiotic ciprofloxacin | USA (USA) | QWB | Self-assessed by children. Face-to-face interview. | 10-18 |
| Peasgood et al (2016) [204] | ADHD; General health (control, siblings of ADHD children) | Community mental health service; Specialist paediatric clinics | UK (UK) | CHU9D, EQ-5D-Y VAS | Self-assessed by children. Self-administered paper questionnaire | Mean: 11.8; 6-18, 10-15 |
| Peetsold et al (2009) [205] | Congenital diaphragmatic hernia; Comorbidities (cardiac abnormalities, neurological deficits) | Intensive care; Hospitalisation | Netherlands (Canada) | HUI3 | Self-assessed by children. Face-to-face interview. | Mean: 10.2 |
| Penn et al (2011) [206] | Cancer patients: brain tumour (ependymoma, germ cell tumour, astrocytoma, medulloblastoma, meningioma, pineoblastoma, craniopharyngioma); Comorbidity (hydrocephalus); General health (control) | Surgery; Chemotherapy; Radiotherapy | UK (Unspecified) | HUI3 | Proxy-assessed by parents or Self-assessed by children. Face-to-face interview. | Median: 11.1, 10.7; 3.6-18.9 |
| Pentek et al (2014) [207] | Duchenne muscular dystrophy; Mucopolysaccharidosis |  | Hungary (Unspecified) | EQ-5D-3L | Proxy-assessed by caregivers. Unspecified mode of administration. | Mean: 9.6 |
| Petersen et al (2017) [208] | General health; Chronic illness (long-term disability, illness or medical conditions; poor self-rated health status) |  | Australia (Australia) | CHU9D | Self-assessed by children. Online survey | 15-17 |
| Petracci and Cavrini (2013) [209] | Overweight or obese; General health (normal weight) | Physical activity | Italy | EQ-5D-Y VAS | Self-assessed by children. Self-administered paper questionnaire. | Unspecified |
| Petrou et al (2007) [210] | Bilateral permanent hearing impairment; Comorbidities (cerebral palsy, asthma, learning disabilities); General health (control) | Cochlear implantation | UK (Canada) | HUI3 | Proxy-assessed by caregivers. Face-to-face interview. | Mean: 7.9; 5.4-11.7 |
| Petrou et al (2009) [211] | Extremely low birthweight; Extremely preterm birth; Comorbidities (cognitive and/or functional disability); General health (control) |  | UK, Ireland (Canada) | HUI3 | Proxy-assessed by parents. Postal survey. | Median: 10.92 |
| Petrou et al (2010) [212] | Otitis media with effusion | Treatment: topical intranasal steroids  Control: placebo nasal spray | UK (UK, Canada) | EQ-5D-3L, HUI2, HUI3 | Proxy-assessed by parents. Self-administered paper questionnaire. | 4-11 |
| Petrou et al (2010b) [213] | Extremely low birthweight; Extremely preterm birth; Comorbidities (ADHD, ASD, behavioural disorder, tic disorder, emotional disorder, cognitive impairment); General health (control) |  | UK, Ireland (UK, Canada) | HUI2, HUI3 | Proxy-assessed by parents. Postal survey. | Median: 10.92 |
| Petrou et al (2011) [214] | Extremely low birthweight; Extremely preterm birth; Comorbidity (neurodevelopmental impairment) |  | UK, Ireland (UK, Canada) | HUI2, HUI3 | Proxy-assessed by parents. Postal survey. | Unspecified |
| Petrou and Kupek (2009) [215] | ADHD; Asthma; ASD; Behavioural disorder; Blood diseases; Bone deformities; Osteogenesis imperfecta; Cardiovascular disease; Cerebral palsy; Chromosomal abnormalities; Down syndrome; Fragile X syndrome; Williams syndrome; CNS disorder; CNS infection (meningitis, encephalitis); Malignant tumour; Non-malignant tumour; Congenital conditions of the connective tissue; Congenital skeletal facial conditions; Cystic fibrosis; Diabetes; Digestive system disorder; Epilepsy; Head injury; Deafness; Hydrocephalus; JIA; Kidney disease; Language and speech disorder; Learning and physical disabilities; Global developmental delay; Metabolic disease; Motor disorder; Muscular dystrophy; Microcephaly; Respiratory disease; Skin disease; Spina bifida; Urogenital disorder; Vision disorder; General health (control) |  | UK (Canada) | HUI3 | Proxy-assessed by caregivers. Postal survey. | 5-16 |
| Philipsson et al (2013) [216] | Internalising disorder: recurrent psychosomatic problems | Treatment: dance classes  Control: primary care | Sweden (Canada) | HUI3 | Self-assessed by children. Face-to-face interview. | 13-18 |
| Pitt et al (2006) [217] | Atopic dermatitis |  | UK (UK) | VAS, Asthma-specific MAUI | Proxy-assessed by parents. Face-to-face interview. | Unspecified |
| Poenaru et al (2017) [218] | Hypothetical congenital conditions requiring surgery: Mild/severe hypospadias; Mild/severe imperforate anus; Hydrocephalus; Mild/severe spina bifida; Hirschsprung’s disease; Mild/severe abdominal wall defect; Intestinal atresia; Undescended testes; Cleft lip; Cleft palate | Surgery | Kenya | VAS, TTO | Proxy-assessed by physicians, nurses, allied health professionals, community members. Self-administered paper questionnaire | Unspecified |
| Pogany et al (2006) [219] | Cancer survivors: leukaemia; lymphoma; brain tumour; SNS tumour; retinoblastoma; renal tumour; hepatic tumour; bone tumour; STS; germ cell tumour; carcinoma; General health (control) | Surgery; Chemotherapy; Radiotherapy | Canada (Canada) | HUI3 | Proxy-assessed by parents or self-assessed by children or assessed together by parents and children. Postal survey. | 5-37 |
| Poley et al (2001) [220] | Imperforate anus | Neonatal surgery | Netherlands (UK) | EQ-5D-3L | Proxy-assessed by parents. Postal survey. | 5-15 |
| Poley et al (2002) [221] | Congenital diaphragmatic hernia | Neonatal surgery; ECMO bridging | Netherlands (UK) | EQ-5D-3L | Proxy-assessed by parents. Postal survey. | 5-15 |
| Polinder et al (2005) [222] | Injury: head; facial; extremity dislocation; internal organ; lower extremity; spinal cord; nerve; whiplash; open wounds; poisoning | Emergency department; Hospitalisation | Netherlands (UK) | EQ-5D-3L VAS,  EQ-5D-3L | Self-assessed by children or assessed together by children and parents. Postal survey. | Mean: 9.6; 5-14 |
| Portwine et al (2016) [223] | Survivors of high-risk neuroblastoma | Hematopoietic stem cell transplantation, High-dose chemotherapy, Immunotherapy; Protocols: CCG 3891, POG 9340/41/42, POG 9640, A3973, ANBL0532, MADDOC, SIOP-COJEC | Canada (Canada) | HUI2, HUI3 | Proxy-assessed by parents. Postal survey | 5-18 |
| Powell et al (2013) [224] | Acute severe asthma | Treatment: nebulised magnesium sulphate  Control: nebulised isotonic saline | UK (UK) | EQ-5D-3L | Proxy-assessed by parents. Postal survey. | Median: 4; 1-15 |
| Prince et al (2010) [225] | Refractory JIA | Etanercept | Netherlands (Canada) | HUI3 | Proxy-assessed by parents. Postal survey. | Median: 11.9 |
| Protudjer et al (2015) [226] | Food allergy | Outpatient care; Epinephrine autoinjector | Sweden (UK) | EQ-5D-3L | Proxy-assessed by parents. Postal survey. | Mean: 6; 0-12 |
| Quinn et al (2004) [227] | Very low birthweight; Very preterm birth; Retinopathy of prematurity | Cryotherapy in neonatal period | USA (Canada) | HUI3 | Proxy-assessed by parents. Face-to-face interview. | Mean: 10 |
| Qvist et al (2004) [228] | End-stage kidney failure: congenital nephrotic syndrome, polycystic kidney disease, prune-belly syndrome, neuroblastoma | Kidney transplant | Finland (Finland) | 17D | Self-assessed by children. Face-to-face interview. | Mean: 9.6; 8-11 |
| Raat et al (2002) [229] | Chronic illness; General health (no disease) |  | Netherlands (Canada) | HUI2 | Proxy-assessed by parents. Postal survey. | 5-13 |
| Raat et al (2004) [230] | General health |  | Netherlands (Canada) | VAS, HUI3 | Proxy-assessed by parents. Postal survey. | 4-13 |
| Rae et al (2008) [231] | Cancer patients: ALL | DFCI 95-001 | USA, Canada (Unspecified) | HUI3 | Proxy-assessed by parents. Self-administered paper questionnaire. | 5-18 |
| Rae et al (2012) [232] | Cancer patients: ALL | DFCI 95-001; DFCI 00-001 | USA, Canada (Unspecified) | HUI3 | Proxy-assessed by parents. Self-administered paper questionnaire. | 5-18 |
| Rae et al (2014) [233] | Cancer survivors: ALL  Cancer patients: ALL | DFCI 95-01; BFM protocol | USA, Canada (Canada) | HUI3 | Proxy-assessed by parents. Self-administered paper questionnaire. | 5-12 |
| Rae et al (2016) [234] | Survivors of Hodgkin’s disease |  | Canada (Canada) | HUI2, HUI3 | Assessed together by children and caregivers. Postal survey | 5-15 |
| Raluy-Callado et al (2013) [235] | Mucopolysaccharidosis type II (Hunter syndrome) | Treatment for joint pain | USA, UK, Brazil, Germany (Canada) | HUI3 | Proxy-assessed by parents. Unspecified mode of administration. | Mean: 14.2; 5-30.9 |
| Raspa et al (2016) [236] | Fragile X syndrome |  | USA (Unspecified) | ABC-UI | Proxy-assessed by parents. Online survey |  |
| Ratcliffe et al (2012) [237] | General health |  | Australia (UK) | CHU9D, HUI2 | Self-assessed by children. Online survey. | Mean: 14; 11-17 |
| Ratcliffe et al (2012b) [238] | General health |  | Australia (UK, Australia) | CHU9D, AQoL-6D | Self-assessed by children. Online survey. | Mean: 15; 11-17 |
| Rautava et al (2009) [239] | Extremely low birthweight; Extremely preterm birth; Very low birthweight; Very preterm birth; General health (control) | Intensive care | Finland (Finland) | 17D | Proxy-assessed by parents. Postal survey. | Mean: 5 |
| Redouane et al (2016) [240] | Combined chronic diseases: Central nervous system disorders; Musculoskeletal disorders; Respiratory disorders; Genetic/metabolic disorders; Cardiac/renal disorders; Immunodeficiency | Invasive medical technologies: Enterostomy tube; Long-term ventilation | Canada (Canada) | HUI2, HUI3 | Proxy-assessed by parents. Self-administered paper questionnaire | Mean: 10.6; 5-18 |
| Renier et al (2012) [241] | Injury in rural area; General health (no injury) |  | USA (Unspecified) | HUI3 | Self-assessed by children. Unspecified mode of administration. | 5-19 |
| Rhodes et al (2011) [242] | Overweight (insulin resistant, prediabetes); Diabetes type II; Diabetes complications (end-stage renal disease, blindness, amputation, heart disease) | Diabetes type II treatment: diet therapy, oral medication, insulin | USA | SG | Proxy-assessed by parents or self-assessed by children. Face-to-face interview. | Mean: 15.5; 12-18 |
| Rhodes et al (2012) [243] | Overweight (insulin resistant, prediabetes); Diabetes type II |  | USA (Canada) | HUI3 | Proxy-assessed by parents or self-assessed by children. Face-to-face interview. | Mean: 15.5; 12-18 |
| Roberts et al (2013) [244] | Extremely low birthweight; Extremely preterm birth; Comorbidities (intraventricular haemorrhage, cerebral palsy, hydrocephalus, cognitive impairment, unilateral/bilateral blindness, deafness); General health (control) | Intensive care: surgery, antenatal corticosteroids, postnatal corticosteroids, exogenous surfactant | Australia (Unspecified) | HUI3 | Self-assessed by children. Self-administered paper questionnaire. | Mean: 17.9, 18.1 |
| Robertson et al (2017) [245] | Obesity | Treatment: “Families for Health” – Information on parenting skills, social and emotional development and healthy eating  Control: Usual care | UK (Unspecified) | EQ-5D-Y, EQ-5D-Y VAS | Self-assessed by children; Proxy-assessed by parents. Self-administered paper questionnaire | Mean: 9.46; 6-11 |
| Robles et al (2015) [246] | General health |  | Spain | EQ-5D-Y VAS | Self-assessed by children. Online survey; Self-administered paper questionnaire | Mean: 11.7; 8-18 |
| Rochanathimoke et al (2014) [247] | Gastroenteritis | Hospitalisation | Thailand (Thailand) | EQ-5D-3L | Proxy-assessed by caregivers. Face-to-face interview. | Mean: 1.77; 0.17-5 |
| Rocque et al (2015) [248] | Spina bifida; Complications (myelomeningocele, closed spinal dysraphism, bowel incontinence) | CSF shunt; Chiari malformation II decompression; Tethered cord release | USA (Unspecified) | HUI3 | Assessed together by children and parents. Self-administered paper questionnaire. | Mean: 12.6; 5-20 |
| Rodriguez-Martinez et al (2013) [249] | Persistent asthma | Treatments: Fluticasone; Ciclesonide; Budesonide;  Control: Beclomethasone dipropionate | Colombia | SG | Proxy-assessed by parents. Face-to-face interview. | Unspecified |
| Roman (2010) [250] | Endometriosis | Laparoscopic excision | New Zealand (UK) | EQ-5D-3L VAS,  EQ-5D-3L | Self-assessed by children. Postal survey or Self-administered paper questionnaire. | Mean: 17.4 |
| Roposch et al (2011) [251] | Developmental hip dysplasia; Complication (secondary osteonecrosis) | Hospitalisation | UK (Canada) | HUI3 | Assessed together by children and caregivers. Face-to-face interview. | Mean: 14; 4-18 |
| Rosenbaum et al (2007) [252] | Cerebral palsy |  | Canada (Canada) | HUI3 | Proxy-assessed by caregivers. Self-administered paper questionnaire. | Mean: 16; 13-20 |
| Rowley et al (1998) [253] | Cystic fibrosis |  | USA | TTO | Assessed together and children and parents. Face-to-face interview. | 5-18 |
| Sach and Barton (2007) [254] | Sensorineural hearing loss: bilateral; unilateral; congenital; acquired | Unilateral cochlear implantation | UK (UK) | EQ-5D-3L VAS,  EQ-5D-3L | Proxy-assessed by parents. Face-to-face interview. | Mean: 9.29 |
| Saigal et al (1994) [255] | Extremely low birthweight; Extremely preterm birth; General health (control) |  | Canada (Canada) | HUI2 | Proxy-assessed by caregivers. Self-administered paper questionnaire. | Mean: 8 |
| Saigal et al (1996) [256] | Extremely low birthweight; Extremely preterm birth; Comorbidities (neurosensory impairment, cerebral palsy, hydrocephalus, cognitive impairment, autism, blindness, deafness); General health (control); Hypothetical health states associated with extremely low birthweight or extremely preterm birth children | Hypothetical neonatal care and use of health equipment | Canada | VAS, SG | Self-assessed by children. Face-to-face interview. | Mean: 14, 14.4; 12-16 |
| Saigal et al (1999) [257] | Hypothetical health states associated with extremely low birthweight or extremely preterm birth children | Hypothetical neonatal care and use of health equipment | Canada | SG | Proxy-assessed by physicians, nurses or parents or self-assessed by children. Face-to-face interview. | Mean: 8, 14.2 |
| Saigal et al (2000) [258] | Extremely low birthweight; Extremely preterm birth; Comorbidities (neurosensory impairment, cerebral palsy, hydrocephalus, cognitive impairment, autism, blindness, deafness); General health (control); Hypothetical health states associated with extremely low birthweight or extremely preterm birth children | Hypothetical neonatal care and use of health equipment | Canada | SG | Proxy-assessed by parents. Face-to-face interview. | Mean: 14, 14.4; 12-16 |
| Saigal et al (2003) [259] | Hypothetical health states associated with extremely low birthweight or extremely preterm birth children | Hypothetical neonatal care and use of health equipment | Canada | SG | Proxy-assessed by parents. Face-to-face interview. | Unspecified |
| Saigal et al (2016) [260] | Extremely low birthweight; Extremely preterm birth; Comorbidity (neurosensory impairment); General health |  | Canada (Canada) | HUI3 | Self-assessed by children. Face-to-face interview | Mean: 13.9; 12-16 |
| Saw et al (2003) [261] | Myopia | Corrective visual aid | Singapore | TTO, SG (Pit: Blindness) | Self-assessed by children. Face-to-face interview. | 15-18 |
| Scalone et al (2007) [262] | Cancer patients: ALL; General health (control) | Unspecified cancer therapy | Italy | EQ-5D-Y VAS | Self-assessed by children. Postal survey or Self-administered paper questionnaire. | 8-15 |
| Scalone et al (2012) [263] | Cancer patients: ALL; General health (control) | Chemotherapy maintenance phase | Italy | EQ-5D-Y VAS | Self-assessed by children. Postal survey or Self-administered paper questionnaire. | 8-15 |
| Scott et al (2017) [264] | General health; Combined congenital physical disabilities (spina bifida, muscle diseases, cerebral palsy); Combined acquired chronic conditions (diabetes, HIV, neurological, heart, renal and respiratory impairments); Combined acute illnesses (appendicitis, septic arthritis, leukaemia, bone fracture) | Intensive care; Nursing care; Wheelchairs and assistive devices | South Africa | EQ-5D-Y VAS | Self-assessed by children. Self-administered paper questionnaire; Face-to-face interview | Mean: 10.5; 8-12 |
| Secnik et al (2005) [265] | ADHD | Stimulant therapy; Psychotherapy; Hypothetical ADHD treatments (nonstimulant therapy, immediate-release stimulant therapy, extended-release stimulant therapy); Varying patient response to treatment | UK (UK) | EQ-5D-3L VAS,  SG (Pit: Death), SG (Pit: Worst health),  EQ-5D-3L | Proxy-assessed by parents. Face-to-face interview. | Mean: 12.6; 7-18 |
| Selvadurai et al (2002) [266] | Cystic fibrosis: pulmonary exacerbation | Treatment: Physical activity (aerobic training, resistance training)  Control: outpatient care (antibiotics, physiotherapy, nutrition supplement) | Australia (USA) | QWB | Proxy-assessed by physicians. Self-administered paper questionnaire. | Mean: 13.2; 8-16 |
| Semenov et al (2013) [267] | Sensorineural hearing loss: congenital; acquired | Cochlear implantation | USA (Canada) | HUI3 | Proxy-assessed by parents. Self-administered paper questionnaire. | Unspecified |
| Seyedifar et al (2016) [268] | Beta thalassemia major; Comorbidities (heart complication, diabetes, hepatitis) | Blood transfusions and iron chelation (injection or oral) | Iran (Iran, US) | EQ-5D-3L, VAS | Assessed together by children and parents. Self-administered paper questionnaire | 10-15; 15-20 |
| Sierwald et al (2016) [269] | Combined dental disorders: dental caries, malocclusion, cleft lip/palate; General health | Orthodontic treatment | Germany (Europe) | EQ-5D-Y, EQ-5D-Y VAS | Self-assessed by children. Self-administered by paper questionnaire | Mean: 11.9, 9.9 |
| Simonova et al (2013) [270] | Cystic fibrosis |  | Russia (Unspecified) | HUI2, HUI3 | Proxy-assessed by parents or self-assessed by children. Unspecified mode of administration. | Mean: 5, 6, 12, 15, 16 |
| Sims-Williams et al (2017) [271] | Spina bifida; Comorbidities (hydrocephalus, immobility) | Spina bifida surgical repair | Uganda (Unspecified) | HUI3, VAS | Self-assessed by children; Proxy-assessed by parents. Face-to-face interview; Self-administered paper questionnaire | 10-14 |
| Smith-Olinde et al (2008) [272] | Hearing loss | Cochlear implantation | USA (USA, Canada) | QWB, HUI3 | Proxy-assessed by caregivers. Self-administered paper questionnaire. | Mean: 7.3; 5-10 |
| Soucie et al (2017) [273] | Haemophilia A |  | USA (USA, UK) | EQ-5D-3L, SF-6D | Self-assessed by children. Self-administered paper questionnaire | 14-20 |
| Sparreboom et al (2012) [274] | Sensorineural hearing loss | Cochlear implantation: bilateral; unilateral | Netherlands (Canada) | VAS, HUI3 | Proxy-assessed by parents. Face-to-face interview. | Mean: 5.3; 2.4-8.5 |
| Sruamsiri et al (2013) [275] | Severe thalassemia | Reduced-intensity hematopoietic stem cell transplantation | Thailand (Thailand) | EQ-5D-3L | Self-assessed by children. Self-administered paper questionnaire. | Mean: 13.7; 9-18 |
| Stade et al (2006) [276] | Fetal alcohol effect; Fetal alcohol syndrome; Comorbidity (severe cognitive impairment); General health (control) | Neonatal care | Canada (Canada) | HUI3 | Proxy-assessed by parents, self-assessed by children or assessed together by children and parents. Face-to-face interview. | Mean: 14.5; 8-21 |
| Stevens and Freeman (2012) [277] | Admission to PICU | Intensive care: mechanical ventilation | UK (Canada) | HUI2, HUI3 | Proxy-assessed by parents or self-assessed by children. Postal survey. | Mean: 10.5; 5-18 |
| Stevens and Ratcliffe (2012) [278] | General health |  | Australia (UK) | CHU9D | Self-assessed by children. Online survey. | Mean: 14.5; 11-17 |
| Stolk et al (2000) [279] | Imperforate anus; General health (control) | Surgery in neonatal period | Netherlands (UK) | EQ-5D-3L VAS,  EQ-5D-3L | Proxy-assessed by parents or assessed together by children and parents. Postal survey. | Mean: 16.26; 1-51 |
| Stromqvist et al (2016) [280] | Lumbar disc herniation | Disc surgery | Sweden | EQ-5D-3L, EQ-5D VAS | Self-assessed by children. Self-administered paper questionnaire | Median: 19; 13-20 |
| Sung et al (2003) [281] | Cancer patients: ALL; Ewing’s sarcoma; Wilm’s tumour; brain tumour; lymphoma; STS; neuroblastoma; osteosarcoma | Chemotherapy | Canada (Canada) | VAS, HUI2, HUI3 | Proxy-assessed by parents. Face-to-face interview. | Mean: 7.2 |
| Sung et al (2003b) [282] | Cancer patients: ALL; Ewing’s sarcoma; Wilm’s tumour; brain tumour; lymphoma; STS; neuroblastoma; osteosarcoma; Arthritis; Haemophilia; Conditions requiring bone marrow transplant | Chemotherapy; Outpatient care | Canada (Canada) | VAS, SG (Pit: Death), SG (Pit: Worst health), TTO, HUI2, HUI3 | Proxy-assessed by parents. Face-to-face interview. | Mean: 8.9; 1-18 |
| Sung et al (2004) [283] | Cancer patients; Arthritis; Haemophilia; Conditions requiring bone marrow transplant; Hypothetical asthma as example of mild disability; Hypothetical stroke as example of moderate disability; Hypothetical injury as example of severe disability |  | Canada (Canada) | VAS, SG, TTO, HUI2, HUI3 | Proxy-assessed by parents or self-assessed by children. Face-to-face interview. | Mean: 13.7; 12-18 |
| Swales et al (2016) [284] | Borderline personality disorder; Recent occurrence of self-harm behaviour | Community mental health service: Dialectical behavioural therapy | UK (Unspecified) | EQ-5D-3L | Self-assessed by children. Online survey | 14-18 |
| Szecket et al (1999) [285] | Cancer survivors: retinoblastoma | Unspecified cancer therapy | Argentina (Canada) | HUI2, HUI3 | Proxy-assessed by physicians. Self-administered paper questionnaire. | Unspecified |
| Tejwani et al (2016) [286] | Hypothetical paediatric vesicoureteral reflux |  | USA | TTO | Proxy-assessed by general public. Online survey | 0-12 |
| Teuffel et al (2011) [287] | Hypothetical cancer patients with febrile neutropenia | Hypothetical cancer therapy: inpatient intravenous antibiotic therapy, outpatient intravenous antibiotic therapy, oral antibiotic therapy, inpatient IV therapy | Canada | SG mapping from VAS | Proxy-assessed by parents. Self-administered paper questionnaire. | Unspecified |
| Thomas et al (2011) [288] | Moderate to severe atopic dermatitis | Treatment: ion-exchange water softener  Control: usual primary care | UK (UK) | EQ-5D-3L | Assessed together by children and parents. Face-to-face interview. | Mean: 5.8, 5.1; 0.5-16 |
| Thorrington et al (2017) [289] | Influenza-like illness: fever, malaise, headache, myalgia, cough, sore throat, shortness of breath; General health (after recovery from influenza-like illness) |  | UK (Unspecified) | EQ-5D-3L | Proxy-assessed by parents. Postal survey | 6-11 |
| Tilford et al (2005) [290] | Spina bifida: sacral lesion, lower lumbar lesion, thoracic lesion; General health (control) |  | USA (Canada) | HUI2 | Proxy-assessed by caregivers. Telephone interview. | 5-17 |
| Tilford et al (2007) [291] | Traumatic brain injury | Intensive care: endotracheal intubation, mechanical ventilation, intracranial pressure monitoring, craniotomy | USA (USA) | QWB | Proxy-assessed by caregivers. Telephone interview. | Mean: 12.6; 5-18 |
| Tilford et al (2012) [292] | ASD: pervasive developmental disorder, Asperger’s disorder |  | USA (USA, Canada) | HUI3, QWB | Proxy-assessed by caregivers. Postal survey. | Mean: 8.6; 4-17 |
| Tilford et al (2015) [293] | ASD: pervasive developmental disorder, Asperger’s disorder |  | USA (USA, Canada) | HUI3, QWB | Proxy-assessed by caregivers. Postal survey. | Mean: 8.2; 4-17.9 |
| Tirado et al (2011) [294] | Laryngotracheal stenosis | Laryngotracheal reconstruction | Canada (Canada) | VAS, HUI3 | Proxy-assessed by parents, self-assessed by children or assessed together by children and parents. Face-to-face interview. | Mean: 9.6; 4-15 |
| Tong et al (2011) [295] | Kidney disease | Successful kidney transplant | Australia (Canada) | VAS, TTO, HUI2, HUI3 | Assessed together by children and parents. Face-to-face interview. | 12-19 |
| Tong et al (2013) [296] | Kidney disease | Failed kidney transplant; Hemodialysis; Peritoneal dialysis | Australia (Canada) | VAS, TTO, HUI2, HUI3, SF-6D | Assessed together by children and parents. Face-to-face or telephone interview. | Mean: 17.1; 12-25 |
| Trent et al (2011) [297] | Pelvic inflammatory disease; Permanent sequelae (ectopic pregnancy, infertility, chronic abdominal pain) | Hospitalisation | USA | VAS, TTO | Proxy-assessed by parents or self-assessed by children. Online survey. | Mean: 16.2; 12-19 |
| Trevino et al (2013) [298] | Overweight or obese |  | USA (Canada) | EQ-5D-3L VAS, HUI2, HUI3 | Self-assessed by children. Self-administered paper questionnaire. | Mean: 11; 10-12 |
| Trudel et al (1998) [299] | Cancer survivors: leukaemia, lymphoma, SNS tumour, renal tumour, hepatic tumour, bone tumour, STS  Cancer patients: leukaemia, lymphoma, solid tumour, brain tumour | Surgery; Chemotherapy; Radiotherapy | Canada (Canada) | HUI2 | Proxy-assessed by parents. Self-administered paper questionnaire. | Mean: 9.1; 4.1-17.3 |
| Ulfsdotter et al (2015) [300] | General health | All Children in Focus: universal parenting programme | Sweden | EQ-5D-Y VAS | Proxy-assessed by parents. Self-administered paper questionnaire. | Mean: 6.09;  3-12 |
| van Baar et al (2011) [301] | Major injury; Burns | Hospitalisation | Netherlands (UK) | EQ-5D-3L | Assessed together by children and parents. Postal survey. | 5-15 |
| van der Kolk et al (2014) [302] | ADHD; General health (control) |  | Netherlands (Unspecified) | EQ-5D-3L | Proxy-assessed by parents. Online survey. | Mean: 11 |
| van Litsenburg et al (2013) [303] | Cancer survivors: ALL | Surgery; Chemotherapy | Netherlands (Canada) | HUI3 | Proxy-assessed by parents. Postal survey. | Mean: 9.3 |
| van Schaik et al (1999) [304] | Cancer survivors: Hodgkin’s disease | Chemotherapy; Radiotherapy | Canada (Canada) | HUI2, HUI3 | Self-assessed by children. Postal survey. | Median: 21.9; 9.4-31.5 |
| van Schooneveld et al (2015) [305] | Epilepsy | Hemispherectomy | Netherlands | EQ-5D-3L VAS | Proxy-assessed by parents. Face-to-face interview. | Median: 9.7, 13.8; 7.1-24.5 |
| Vermeulen et al (2017) [306] | Hypothetical behavioural disorders: Oppositional defiant disorder; Conduct disorder; Disruptive behavioural disorder; Comorbidities (ADHD, substance abuse); General health |  | Netherlands | EQ-5D VAS | Proxy-assessed by professionals working with children or adolescents; Self-assessed by children; Proxy-assessed by parents.  Postal survey; Self-administered paper questionnaire | Mean: 9, 15 |
| Vermeulen et al (2017b) [307] | Antisocial personality disorder: Chronically antisocial and seriously violent | Treatment: Multisystemic therapy  Control: Usual care – juvenile justice and child welfare services | Netherlands (UK) | EQ-5D-3L | Self-assessed by children; Proxy-assessed by parents. Self-administered paper questionnaire | Mean: 16;  12-18 |
| Verrips et al (2001) [308] | Very low birthweight; Very preterm birth |  | Netherlands (Canada) | HUI3 | Proxy-assessed by parents or self-assessed by children. Postal survey or face-to-face or telephone interview. | Mean: 14.3 |
| Verrips et al (2008) [309] | Extremely low birthweight; Extremely preterm birth | Prenatal steroid treatment; Ventilation | Canada, Germany, Netherlands (Canada) | HUI3 | Self-assessed by children. Postal survey or face-to-face interview. | Mean: 14; 12-16 |
| Verrips et al (2012) [310] | Very low birthweight; Very preterm birth |  | Netherlands (Canada) | HUI3 | Self-assessed by children. Postal survey. | Mean: 14 |
| Vitale et al (2001) [311] | JIA; Cerebral palsy; Scoliosis | Surgery | USA (Unspecified) | EQ-5D | Self-assessed by children. Self-administered paper questionnaire. | Mean: 14.6; 10-18 |
| Wake et al (2015) [312] | Speech disorder: receptive and/or expressive language delay at 4 years old | Speech therapy: Language for Learning (population-based intervention) | Australia | HUI3 | Proxy-assessed by parents. Self-administered paper questionnaire. | Mean: 6 |
| Wang et al (2002) [313] | Health states taken from Barr et al (1993), Kennedy and Leyland (1999), Barr et al (1999), Saigal et al (1994), Van Schaik et al (1999), Barr et al (2000); General health (control) |  | UK, Canada (Singapore) | HUI2 | Proxy-assessed by nurses, physicians or parents or assessed together by children and parents. Postal survey or Self-administered paper questionnaire. | 1.7-31.5 |
| Wasserman et al (2005) [314] | Haemophilia | Haemophilia treatment: on-demand; prophylaxis; inhibitor | USA | VAS, SG | Assessed together by children and parents. Face-to-face interview. | Mean: 10; 0.5-18 |
| Weigel et al (2016) [315] | Anorexia nervosa | Inpatient or outpatient mental health services | Germany | EQ-5D VAS | Self-assessed by children. Self-administered paper questionnaire | Mean: 15.6; 11-18 |
| Weiner et al (2016) [316] | Cancer survivors: Osteosarcoma, Ewing’s sarcoma, Myxoid liposarcoma, Fibromatosis | Unspecified cancer therapy | UK | EQ-5D VAS | Self-assessed by children. Self-administered paper questionnaire | 12-18 |
| Wijnen et al (2017) [317] | Epilepsy: Uncontrolled seizures, Not eligible for surgery | Treatment: Ketogenic diet  Control: Usual care | Netherlands (Netherlands) | EQ-5D-Y | Proxy-assessed by parents. Self-administered paper questionnaire | Mean: 7.8, 8.1;  2.1-16.5 |
| Wille et al (2010) [318] | General health |  | Germany, Spain | EQ-5D VAS;  EQ-5D-Y VAS | Self-assessed by children. Self-administered paper questionnaire | Mean: 13.9, 13;  10-18; 8-18 |
| Willems et al (2007) [319] | Low to moderately severe asthma | Treatment: Nurse-led telemonitoring for asthma symptoms  Control: usual care | Netherlands (UK) | EQ-5D-3L VAS,  EQ-5D-3L | Assessed together by children and parents. Self-administered paper questionnaire. | Mean: 10.6, 10.9; 7-18 |
| Willems et al (2009) [320] | Combined chronic illnesses: Asthma, Rheumatic disorders, Diabetes, Speech/language and/or hearing disorders |  | Netherlands | EQ-5D-Y VAS | Assessed together by children and parents. Postal survey | 7-18 |
| Wolke et al (2013) [321] | Very low birthweight; Very preterm birth; General health (control) |  | Germany (Canada) | HUI3 | Proxy-assessed by parents or self-assessed by children. Postal survey. | Mean: 13 |
| Wong et al (2017) [322] | Adolescent idiopathic scoliosis | Surgery, Braces | China (China) | EQ-5D-5L | Self-assessed by children. Self-administered paper questionnaire. | Mean: 15.5 |
| Wright et al (2003) [323] | Cancer survivors: ALL; Standard or high risk of relapse; General health (control) | DFCI 85-01; DFCI 87-01; DFCI 91-01; DFCI 95-01 | Canada (Unspecified) | HUI2, HUI3 | Assessed together by children and parents. Self-administered paper questionnaire. | Mean: 12.1, 11.9; 7-18.9 |
| Wright et al (2005) [324] | Cancer survivors: ALL; Standard or high risk of relapse; General health (control) | DFCI 85-01; DFCI 87-01; DFCI 91-01; DFCI 95-01 | Canada (Unspecified) | HUI3 | Proxy-assessed by parents. Self-administered paper questionnaire. | Mean: 12.2; 5.1-31.5 |
| Wright et al (2017) [325] | Depression | Treatment: Computerised cognitive behavioural therapy (Stressbusters)  Control: Accessing low-mood self-help website | UK (Unspecified) | EQ-5D-Y | Self-assessed by children. Self-administered paper questionnaire. | Mean: 15.5; 12-18 |
| Wu et al (2010) [326] | General health | REAL Kids Alberta programme to promote healthy body weight | Canada (USA) | EQ-5D-Y VAS,  EQ-5D-Y | Self-assessed by children. Self-administered paper questionnaire. | 10-11 |
| Wu et al (2012) [327] | Overweight or obese; General health (normal weight) | REAL Kids Alberta programme to promote healthy body weight; Physical activity | Canada | EQ-5D-Y VAS | Self-assessed by children. Self-administered paper questionnaire. | 10-11 |
| Wu et al (2014) [328] | General health | REAL Kids Alberta programme to promote healthy body weight | Canada (USA, Canada) | EQ-5D-Y VAS,  EQ-5D-Y | Self-assessed by children. Self-administered paper questionnaire. | 10-11 |
| Xu et al (2014) [329] | General health | Physical activity | China (UK, Australia) | CHU9D | Self-assessed by children. Self-administered paper questionnaire. | Mean: 14.1; 9-17 |
| Yaris et al (2001) [330] | Cancer patients: NHL; Hodgkin’s disease; brain tumour; neuroblastoma; germ cell tumour; STS; bone sarcoma | Surgery; Chemotherapy; Radiotherapy | Turkey (Canada) | HUI2 | Proxy-assessed by parents. Face-to-face interview. | Mean: 9.7; 2-16 |
| Yi et al (2003) [331] | Cystic fibrosis |  | USA (Canada) | VAS, TTO, SG, HUI2 | Self-assessed by children. Face-to-face interview. | Mean: 15.1; 12-18 |
| Yi et al (2009) [332] | IBD: Crohn’s disease; Ulcerative colitis; General health (control) |  | USA | VAS, TTO, SG | Self-assessed by children. Face-to-face interview. | Mean: 14.8, 15.5; 11-19 |
| Young et al (2010) [333] | Cerebral palsy |  | Canada (Canada) | HUI3,  AQoL-5D | Assessed together by children and caregivers. Postal survey. | Mean: 15.5; 13-17.9 |
| Young et al (2013) [334] | Spina bifida: thoracic lesion; high-lumbar lesion; low-lumbar lesion; sacral lesion |  | Canada (Canada) | HUI3,  AQoL-5D | Assessed together by children and caregivers. Postal survey. | Mean: 16; 13-17.9 |
| Rodriguez-Martinez et al (2016) [335] | Asthma: Suboptimal control, no exacerbation; Exacerbation; General health control |  | Colombia | SG | Proxy-assessed by parents. Face-to-face interview. | Unspecified |
| CBT: Cognitive-behavioural therapy; RCT: Randomised controlled trial; LCHADD: Long-chain acyl-CoA dehydrogenase deficiency; MCADD: Medium-chain acyl-CoA dehydrogenase deficiency; ALL: Acute lymphoblastic leukaemia; DFCI: Dana-Farber Cancer Institute; PNET: Primitive neuroectodermal tumour; NHL: Non-Hodgkin’s lymphoma; STS: Soft tissue sarcoma; AML: Acute myeloid leukaemia; ADHD: Attention-deficit hyperactivity disorder; MSKD: Musculoskeletal disorder; IBD: Inflammatory bowel disease; JIA: Juvenile idiopathic arthritis; PICU: Paediatric intensive care unit; DCM: Demands and capacities model; ASD: Autism spectrum disorder; PTSD: Post-traumatic stress disorder; HFO: High-frequency oscillation; BFM: Berlin-Frankfurt-Munster; CSF: Cerebral spinal fluid; REAL: Raising healthy eating and active living; CH-6D: Child Health 6 Dimensions; ABC-UI: Aberrant Behaviour Checklist Utility Index | | | | | | |

**References for studies in Table S3.**

1. Aldebayan S, Sinno H, Makhdom A, Ouellet JA, Saran N. Impact of living with scoliosis: a utility outcome score assessment. Spine. 2017;42(2):e93-e97.

2. Anderson R, Ukoumunne OC, Sayal K, Phillips R, Taylor JA, et al. Cost-effectiveness of classroom-based cognitive behaviour therapy in reducing symptoms of depression in adolescents: a trial-based analysis. J Child Psychol Psychiatry. 2014;55(12):1390-97.

3. Angelis A, Kanavos P, Lopez-Bastida J, Linertova R, Oliva-Moreno J, et al. Social/economic costs and health-related quality of life in patients with epidermolysis bullosa in Europe. Eur J Health Econ. 2016;17Supp1:31-42.

4. Apajasalo M, Sintonen H, Holmberg C, Sinkkonen J, Aalberg V, et al. Quality of life in early adolescence: a sixteen-dimensional health-related measure (16D). Qual Life Res. 1996;5(2):205-11.

5. Apajasalo M, Rautonen J, Holmberg C, Sinkkonen J, Aalberg V, et al. Quality of life in pre-adolescence: a 17-dimensional health-related measure (17D). Qual Life Res. 1996;5(6):532-38.

6. Apajasalo M, Sintonen H, Rautonen J, Kaitila I. Health-related quality of life of patients with genetic skeletal dysplasias. Eur J Pediatr. 1998;157(2):114-21.

7. Arkkila E, Rasanen P, Roine RP, Sintonen H, Saar V, et al. Health-related quality of life of adolescents with childhood diagnosis of specific language impairment. Int J Pediatr Otorhinolaryngol. 2009;73(9):1288-96.

8. Arkkila E, Rasanen P, Roine RP, Sintonen H, Saar V, et al. Health-related quality of life of children with specific language impairment aged 8-11. Folia Phoniatr Logop. 2011;63(1):27-35.

9. Arnold S, Jin Z, Weinberg A, Bishop J, Sands S, et al. Allogeneic stem cell transplantation for children with sickle cell disease achieves quality of life similar to normal children and is cost effective. Biol Blood Marrow Transplant. 2014;20(2 SUPPL.1):S79.

10. Autti-Ramo I, Makela M, Sintonen H, Koskinen H, Laajalahti L, et al. Expanding screening for rare metabolic disease in the newborn: an analysis of costs, effect and ethical consequences for decision-making in Finland. Acta Paediatrica. 2005;94(8):1126-36.

11. Baguelin M, Hoek AJ, Jit M, Flasche S, White PJ, et al. Vaccination against pandemic influenza A/H1N1v in England: a real-time economic evaluation. Vaccine. 2010;28(12):2370-84.

12. Balottin U, Ferri M, Racca M, Rossi M, Rossi, G, et al. Psychotherapy versus usual care in pediatric migraine and tension-type headache: a single-blind controlled pilot study. Ital J Pediatr. 2014;40(6).

13. Banks BA, Barrowman NJ, Klaassen R. Health-related quality of life: changes in children undergoing chemotherapy. J Pediatr Hematol Oncol. 2008;30(4):292-97.

14. Barr RD, Furlong W, Dawson S, Whitton AC, Strautmanis I, et al. An assessment of global health status in survivors of acute lymphoblastic leukemia in childhood. J Pediatr Hematol Oncol. 1993;15(3):284-90.

15. Barr RD, Pai MKR, Weitzman S, Feeny D, Furlong W, et al. A multiattribute approach to health-status measurement and clinical management illustrated by an application to brain-tumors in childhood. Int J Oncol. 1994;4(3):639-48.

16. Barr RD, Petrie C, Furlong W, Rothney M, Feeny D. Health-related quality of life during post-induction chemotherapy in children with acute lymphoblastic leukemia in remission: an influence of corticosteroid therapy. Int J Oncol. 1997;11(2):333-39.

17. Barr RD, Simpson T, Whitton A, Rush B, Furlong W, et al. Health-related quality of life in survivors of tumours of the central nervous system in childhood – a preference-based approach to measurement in a cross-sectional study. Eur J Cancer. 1999;35(2):248-55.

18. Barr RD, Chalmers D, de Pauw S, Furlong W, Weitzman S, et al. Health-related quality of life in survivors of Wilms' tumor and advanced neuroblastoma: a cross-sectional study. J Clin Oncol. 2000;18(18):3280-87.

19. Barr RD, Gonzalez A, Longchong M, Furlong W, Vizcaino MP, et al. Health status and health-related quality of life in survivors of cancer in childhood in Latin America: a MISPHO feasibility study. Int J Oncol. 2001;19(2):413-21.

20. Barton GR, Stacey PC, Fortnum HM, Summerfield AQ. Hearing-impaired children in the United Kingdom, IV: cost-effectiveness of pediatric cochlear implantation. Ear Hear. 2006;27(5):575-88.

21. Baumann N, Bartmann P, Wolke D. Health-related quality of life into adulthood after very preterm birth. Pediatrics. 2016;137(4).

22. Beckman L, Svensson M, Frisen A. Preference-based health-related quality of life among victims of bullying. Qual Life Res. 2016;25(2):303-9.

23. Beerthuizen T, Voorend-van Bergen S, van den Hout WB, Vaessen-Verberne AA, Brackel HJ. Cost-effectiveness of FENO-based and web-based monitoring in paediatric asthma management: a randomised controlled trial. Thorax. 2016;71(7):607-13.

24. Belfort MB, Zupanic JAF, Riera KM, Turner JHG, Prosser LA. Health state preferences associated with weight status in children and adolescents. BMC Pediatr. 2011;11(12).

25. Bennett JE, Sumner W, Downs SM, Jaffe DM. Parents' utilities for outcomes of occult bacteremia. Arch Pediatri Adolesc Med. 2000;154(1):43-48.

26. Bergfors S, Astrom M, Burstrom K, Egmar A. Measuring health-related quality of life with the EQ-5D-Y instrument in children and adolescents with asthma. Acta Paediatr. 2015;104(2):167-73.

27. Bilcke J, Ogunjimi B, Marais C, de Smet F, Callens M, et al. The health and economic burden of chickenpox and herpes zoster in Belgium. Epidemiol Infect. 2012;140(11):2096-109.

28. Bilcke J, Hens N, Beutels P. Quality-of-life: a many-splendored thing? Belgian population norms and 34 potential determinants explored by beta regression. Qual Life Res. 2017;26:2011-23.

29. Billson AL, Walker DA. Assessment of health status in survivors of cancer. Arch Dis Child. 1994;70(3):200-4.

30. Bodden DH, Dirksen CD, Bogels SM, Nauta MH, de Haan E, et al. Costs and cost-effectiveness of family CBT versus individual CBT in clinically anxious children. Clin Child Psychol Psychiatry. 2008;13(4):543-64.

31. Bolton K, Kremer P, Rossthorn N, Moodie M, Gibbs L, et al. The effect of gender and age on the association between weight status and health-related quality of life in Australian adolescents. BMC Public Health. 2014;14:898-905.

32. Bolton KA, Jacka F, Allender S, Kremer P, Gibbs L, et al. The association between self-reported diet quality and health-related quality of life in rural and urban Australian adolescents. Aust J Rural Health. 2016;24(5):317-25.

33. Boran P, Horsman J, Tokuc G, Furlong W, Muradoglu PU, et al. Translation and cultural adaptation of health utilities index with application to pediatric oncology patients during neutropenia and recovery in Turkey. Pediatr Blood Cancer. 2011;56(5):812-7.

34. Boulton M, Haines L, Smyth D, Fielder A. Health-related quality of life of children with vision impairment or blindness. Dev Med Child Neurol. 2006;48(8):656-61.

35. Bouwmans C, van der Kolk A, Oppe M, Schawo S, Stolk E, et al. Validity and responsiveness of the EQ-5D and the KIDSCREEN-10 in children with ADHD. Eur J Health Econ. 2014;15(9):967-77.

36. Boyer N, Miller S, Connolly P, McIntosh E. Use of the child health utility and strengths and difficulties outcome measures in economic evaluations of school-based interventions: data from a cluster-randomised controlled trial in Northern Ireland. Lancet. 2014;384:21.

37. Boyer NR, Miller S, Connolly P, McIntosh E. Paving the way for the use of the SDQ in economic evaluations of school-based population health interventions: an empirical analysis of the external validity of SDQ mapping algorithms to the CHU9D in an educational setting. Qual Life Res. 2016;25(4):913-23.

38. Boyle SE, Jones GL, Walters SJ. Physical activity, quality of life, weight status and diet in adolescents. Qual Life Res. 2010;19(7):943-54.

39. Braam KI, van Dijk-Lokkart EM, van Dongen JM, van Litsenburg RR, Takken T, et al. Cost-effectiveness of a combined physical exercise and psychosocial training intervention for children with cancer: Results from the quality of life in motion study. Eur J Cancer Care. 2016:1-11.

40. Bradlyn AS, Harris CV, Warner JE, Ritchey AK, Zaboy K. An investigation of the validity of the quality of Well-Being Scale with pediatric oncology patients. Health Psychol. 1993;12(3):246-50.

41. Brisson M, Senecal M, Drolet M, Mansi JA. Health-related quality of life lost to rotavirus-associated gastroenteritis in children and their parents: a Canadian prospective study. Pediatr Infect Dis J. 2010;29(1):73-5.

42. Brown KL, Wray J, Wood TL, McMahon AM, Burch M, et al. Cost utility evaluation of extracorporeal membrane oxygenation as a bridge to transplant for children with end-stage heart failure due to dilated cardiomyopathy. J Heart Lung Transplant. 2009;28(1):32-8.

43. Brunner HI, Maker D, Grundland B, Young NL, Blanchette V, et al. Preference-based measurement of health-related quality of life (HRQL) in children with chronic musculoskeletal disorders (MSKDs). Med Decis Making. 2003;23(4):314-22.

44. Brunner HI, Klein-Gitelman MS, Miller MJ, Trombley M, Baldwin N, et al. Health of children with chronic arthritis: relationship of different measures and the quality of parent proxy reporting. Arthritis Rheum. 2004;51(5):763-73.

45. Brussoni M, Kruse S, Walker K, Validity and reliability of the EQ-5D-3L among a paediatric injury population. Health Qual Life Outcomes. 2013;11:157-65.

46. Bull KS, Kennedy CR, Bailey S, Ellison DW, Clifford SC. Improved health-related quality of life outcomes associated with SHH subgroup medulloblastoma in SIOP-UKCCSG PNET3 trial survivors. Acta Neuropathologica. 2014;128(1):151-3.

47. Burstrom K, Svartengren M, Egmar A. Testing a Swedish child-friendly pilot version of the EQ-5D instrument – initial results. Eur J Public Health. 2011;21(2):178-83.

48. Burstrom K, Bartonek A, Brostrom EW, Sun S, Egmar A. EQ-5D-Y as a health-related quality of life measure in children and adolescents with functional disability in Sweden: testing feasibility and validity. Acta Paediatr. 2014;103(4):426-35.

49. Buysse CMP, Raat H, Hazelzet JA, Hulst JM, Cransberg K, et al. Long-term health status in childhood survivors of meningococcal septic shock. Arch Pediatr Adolesc Med. 2008;162(11):1036-41.

50. Byford S, Barrett B, Roberts C, Wilkinson P, Dubicka B, et al. Cost-effectiveness of selective serotonin reuptake inhibitors and routine specialist care with and without cognitive–behavioural therapy in adolescents with major depression. Br J Psychiatry. 2007;191(6):521-7.

51. Byford S. The validity and responsiveness of the EQ-5D measure of health-related quality of life in an adolescent population with persistent major depression. J Ment Health. 2013;22(2):101-10.

52. Canaway AG, Frew EJ. Measuring preference-based quality of life in children aged 6-7 years: a comparison of the performance of the CHU-9D and EQ-5D-Y – the WAVES pilot study. Qual Life Res. 2013;22(1):173-83.

53. Canaway AG, Frew EJ. Is utility-based quality of life in children aged 6-7 years affected by Body Mass Index (BMI)?. Int J Obes (Lond). 2014;38(8):1146.

54. Cardarelli C, Cereda C, Masiero L, Viscardi E, Faggin R, et al. Evaluation of health status and health-related quality of life in a cohort of Italian children following treatment for a primary brain tumor. Pediatr Blood Cancer. 2006;46(5):637-44.

55. Carroll AE, Downs SM. Improving decision analyses: parent preferences (utility values) for pediatric health outcomes. J Pediatrics. 2009; 155:21-5.

56. Chadha NK, Allegro J, Barton M, Hawkes M, Harlock H, et al. The quality of life and health utility burden of recurrent respiratory papillomatosis in children. Otolaryngol Head Neck Surg. 2010;143(5):685-90.

57. Chen CL, Kuppermann M, Caughey AB, Zane LT. A community-based study of acne-related health preferences in adolescents. Arch Dermatol. 2008;144(8):988-94.

58. Chen G, Ratcliffe J, Olds T, Magarey A, Jones M, et al. BMI, health behaviors, and quality of life in children and adolescents: a school-based study. Pediatrics. 2014;133(4):e868-74.

59. Chen G, Flynn T, Stevens K, Brazier J, Huynh E, et al. Assessing the Health-Related Quality of Life of Australian Adolescents: An Empirical Comparison of the Child Health Utility 9D and EQ-5D-Y Instruments. Value Health. 2015;18(4):432-8.

60. Cheng AK, Rubin HR, Powe NR, Mellon NK, Francis HW, et al. Cost-utility analysis of the cochlear implant in children. JAMA. 2000;284(7):850-6.

61. Cheng S, Teuffel O, Ethier MC, Diorio C, Martino J, et al. Health-related quality of life anticipated with different management strategies for paediatric febrile neutropaenia. Br J Cancer. 2011;105(5):606-11.

62. Cheung PW, Wong CK, Samartzis D, Luk KD, Lam CL, et al. Psychometric validation of the EuroQoL 5-Dimension 5-Level (EQ-5D-5L) in Chinese patients with adolescent idiopathic scoliosis. Scoliosis Spinal Disord. 2016;11:19-30.

63. Chevreul K, Brigham KB, Michel M, Rault G, et al. Costs and health-related quality of life of patients with cystic fibrosis and their carers in France. J Cyst Fibros. 2015;14(3):384-91.

64. Chevreul K, Brigham KB, Brunn M, des Portes V, et al. Fragile X syndrome: economic burden and health-related quality of life of patients and caregivers in France. J Intellect Disabil Res. 2015;59(12):1108-20.

65. Chiou CF, Weaver MR, Bell MA, Lee TA, Krieger JW. Development of the multi-attribute Pediatric Asthma Health Outcome Measure (PAHOM). Int J Qual Health Care. 2005;17(1):23-30.

66. Chirivella S, Rajappa S, Sinha S, Eden T, Barr RD. Health-related quality of life among children with cancer in Hyderabad, India. Indian J Pediatr. 2009;76(12):1231-5.

67. Clement ND, Vats A, Duckworth AD, Gaston MS, Murray AW. Slipped capital femoral epiphysis: is it worth the risk and cost not to offer prophylactic fixation of the contralateral hip? Bone Joint J 2015;97-B;1428-34.

68. Covaciu C, Bergstrom A, Lind T, Svartengren M, Kull I. Childhood allergies affect health-related quality of life. J Asthma. 2013;50(5):522-8.

69. Cox CL, Lensing S, Rai SN, Hinds P, Burghen E, et al. Proxy assessment of quality of life in pediatric clinical trials: application of the Health Utilities Index 3. Qual Life Res. 2005;14(4):1045-56.

70. Creswell C, Violato M, Fairbanks H, White E, Parkinson M, et al. Clinical outcomes and cost-effectiveness of brief guided parent-delivered cognitive behavioural therapy and solution-focused brief therapy for treatment of childhood anxiety disorders: a randomised controlled trial. Lancet Psychiatry 2017;4(7):529-39.

71. Cunha F, Mota T, Teixeira-Pinto A, Carvalho L, Estrada J, et al. Factors associated with health-related quality of life changes in survivors to pediatric intensive care. Pediatr Crit Care Med. 2013;14(1):e8-15.

72. Czyzewski DI, Mariotto MJ, Bartholomew K, LeCompte SH, Sockrider MM. Measurement of quality of well being in a child and adolescent cystic fibrosis population. Med Care. 1994;32(9):965-72.

73. Dakin H, Petrou S, Haggard M, Benge S, Williamson I. Mapping analyses to estimate health utilities based on responses to the OM8-30 Otitis Media Questionnaire. Qual Life Res. 2010;19(1):65-80.

74. de Bruin EJ, van Steensel FJ, Meijer AM. Cost-effectiveness of group and internet cognitive behavioural therapy for insomnia in adolescents: results from a randomised controlled trial. Sleep 2016;39(8):1571-81.

75. de Jong T, Maliepaard M, Bannink N, Raat H, Mathijssen IMJ. Health-related problems and quality of life in patients with syndromic and complex craniosynostosis. Childs Nervous System. 2012;28(6):879-82.

76. de Keizer NF, Bonsel GJ, Gemke RJ, Health status prediction in critically ill children: a pilot study introducing Standardized Health Ratios. Qual Life Res. 1997;6(2):192-9.

77. de Kinderen RJA, Lambrechts DAJE, Wijnen BFM, Postulart D, Aldenkamp AP, et al. An economic evaluation of the ketogenic diet versus care as usual in children and adolescents with intractable epilepsy: an interim analysis. Epilepsia. 2016;57(1):41-50.

78. de Lissovoy G, Matza LS, Green H, Werner M, Edgar T. Cost-effectiveness of intrathecal baclofen therapy for the treatment of severe spasticity associated with cerebral palsy. J Child Neurol. 2007;22(1):49-59.

79. de Sonneville-Koedoot C, Bouwmans C, Franken M, Stolk E. Economic evaluation of stuttering treatment in preschool children: the RESTART study. J Commun Disord. 2015;58:106-18.

80. de Wolf MJF, Hol MKS, Mylanus EAM, Snik AFM, Cremers WRJ. Benefit and quality of life after bone-anchored hearing aid fitting in children with unilateral or bilateral hearing impairment. Arch Otolaryngol Head Neck Surg. 2011;137(2):130-8.

81. Diarbakerli E, Grauers A, Gerdhem P. Population-based normative data for the Scoliosis Research Society 22r questionnaire in adolescents and adults, including a comparison with EQ-5D. Eur Spine J 2017;26(6):1631-7.

82. Dillman JR, Carlos RC, Smith EA, Davenport MS, et al. Relationship of bowl MR imaging to health-related quality of life measures in newly diagnosed pediatric small bowel Crohn disease. Radiology 2016;280(2):568-75.

83. Domellof E, Hedlund L, Odman P. Health-related quality of life of children and adolescents with functional disabilities in a northern Swedish county. Qual Life Res. 2014;23(6):1877-82.

84. Domino ME, Burns BJ, Silva SG, Kratochvil CJ, Vitiello B, et al. Cost-effectiveness of treatments for adolescent depression: results from TADS. Am J Psychiatry. 2008;165:588-96.

85. Domino ME, Foster M, Vitiello B, Kratochvil CJ, Burns BJ, et al. Relative cost-effectiveness of treatments for adolescent depression: 36-week results from the TADS randomised trial. J Am Acad Child Adolesc Psychiatry. 2009;48(7):711-20.

86. Duckworth J, Nayiager T, Pullenayegum E, Whitton A, Hollenberg R, et al. Health-related Quality of Life in Long-term Survivors of Brain Tumors in Childhood and Adolescence: A Serial Study Spanning a Decade. J Pediatr Hematol Oncol. 2015;37(5):362-7.

87. Ebrahim S, Parshuram C. Comparison of utility scores from the Visual Analog Scale and Health Utilities Index 3 in children following pediatric intensive care unit admission. J Child Health Care. 2015;19(1):53-62.

88. Eidt-Koch D, Mittendorf T, Greiner W. Cross-sectional validity of the EQ-5D-Y as a generic health outcome instrument in children and adolescents with cystic fibrosis in Germany. BMC Pediatr. 2009;9:55-62.

89. Ekert H, Brewin T, Boey W, Davey P, Tilden D. Cost-utility analysis of recombinant factor VIIa (NovoSeven) in six children with long-standing inhibitors to factor VIII or IX. Haemophilia. 2001;7(3):279-85.

90. Engelmann G, Erhard D, Petersen M, Parzer P, Schlarb AA, et al. Health-related quality of life in adolescents with inflammatory bowel disease depends on disease activity and psychiatric comorbidity. Child Psychiatry Hum Dev. 2015;46(2):300-7.

91. Epps H, Ginnelly L, Utley M, Southwood T, Gallivan S, et al. Is hydrotherapy cost-effective? A randomised controlled trial of combined hydrotherapy programmes compared with physiotherapy land techniques in children with juvenile idiopathic arthritis. Health Technol Assess. 2005;9(39):iii-iv,ix-x,1-59.

92. Ersberg A, Gerdhem P. Pre-and postoperative quality of life in patients treated for scoliosis Initial experiences with the SweSpine Registry. Acta Orthopaedica. 2013;84(6):537-43.

93. Fantaguzzi C, Allen E, Miners A, Christie D, Opondo C, et al. Health-related quality of life associated with bullying and aggression: a cross-sectional study in English secondary schools. Eur J Health Econ 2017.

94. Feenstra DJ, Hutsebaut J, Laurenssen EMP, Verheul R, Busschbach JJV, et al. The burden of disease among adolescents with personality pathology: quality of life and costs. J Pers Disord. 2012;26(4):593-604.

95. Feeny D, Furlong W, Saigal S, Sun J. Comparing directly measured standard gamble scores to HUI2 and HUI3 utility scores: group- and individual-level comparisons. Soc Sci Med. 2004;58(4):799-809.96.

96. Felder-Puig R, Frey E, Sonnleithner G, Feeny D, Gadner H, et al. German cross-cultural adaptation of the Health Utilities Index and its application to a sample of childhood cancer survivors. Eur J Pediatr. 2000;159(4):283-8.

97. Fluchel M, Horsman JR, Furlong W, Castillo L, Alfonz Y, et al. Self and proxy-reported health status and health-related quality of life in survivors of childhood cancer in Uruguay. Pediatr Blood Cancer. 2008;50(4):838-43.

98. Foreman NK, Faestel PM, Pearson J, Disabato J, Poole M, et al. Health status in 52 long-term survivors of pediatric brain tumors. J Neurooncol. 1999;41(1):47-53.

99. Foster Page LA, Beckett DM, Cameron CM, Thomson WM. Can the Child Health Utility 9D measure be useful in oral health research?. Int J Paediatr Dent. 2015;25(5):349-57.

100. Frew EJ, Pallan M, Lancashire E, Hemming K, Adab P, et al. Is utility-based quality of life associated with overweight in children? Evidence from the UK WAVES randomised controlled study. BMC Pediatr. 2015;15(1):211-20.

101. Friedman JY, Reed SD, Weinfurt KP, Kahler KH, Walter EB, et al. Parents' reported preference scores for childhood atopic dermatitis disease states. BMC Pediatr. 2004;4(1):1-8.

102. Fu L, Talsma D, Baez F, Bonilla M, Moreno B, et al. Measurement of health-related quality of life in survivors of cancer in childhood in Central America: feasibility, reliability, and validity. J Pediatr Hematol Oncol. 2006;28(6):331-41.

103. Furber G, Segal L. The validity of the Child Health Utility instrument (CHU9D) as a routine outcome measure for use in child and adolescent mental health services. Health Qual Life Outcomes. 2015;13:22-35.

104. Furlong W, Rae C, Feeny D, Gelber RD, Laverdiere C, et al. Health-related quality of life among children with acute lymphoblastic leukemia. Pediatr Blood Cancer. 2012;59(4):717-24.

105. Gaitan-Lopez DF, Correa-Bautista JE, Vinaccia S, Ramirez-Velez R. Self-report health-related quality of life among children and adolescents from Bogota, Colombia. The FUPRECOL study. Colomb Med 2017;48(1):12-8.

106. Garcia-Gordillo MA, Adsuar JC, Olivares PR. Normative values of EQ-5D-5L: in a Spanish representative population sample from Spanish Health Survey, 2011. Qual Life Res 2016;25(5):1313-21.

107. Geneid A, Pakkasjarvi N, Aherto A, Roine R, Sintonen H, et al. Outcomes of early infancy laryngeal reconstruction on health- and voice-related quality of life. Int J Pediatr Otorhinolaryngol. 2011;75(3):351-5.

108. Gerald JK, Mcclure LA, Harrington KF, Moore T, Hernandez-Martinez AC, et al. Measurement characteristics of the pediatric asthma health outcome measure. J Asthma. 2012;49(3):260-6.109. Glaser AW, Furlong W, Walker DA, Fielding K, Davies K, et al. Applicability of the Health Utilities Index to a population of childhood survivors of central nervous system tumours in the U.K. Eur J Cancer. 1999;35(2):256-61.

110. Glotzer DE, Freedberg KA, Bauchner H. Management of childhood lead poisoning: clinical impact and cost-effectiveness. Med Dec Making. 1995;15(1):13-24.

111. Goorden M, van der Schee E, Hendriks VM, Hakkaart-van Roijen L. Cost-effectiveness of multidimensional family therapy compared to cognitive behavioral therapy for adolescents with a cannabis use disorder: Data from a randomized controlled trial. Drug Alcohol Depend 2016;162:154-61.

112. Gospodarevskaya E. Post-traumatic stress disorder and quality of life in sexually abused Australian children. J Child Sex Abuse. 2013;22(3):277-96.

113. Grano N, Karjalainen M, Edlund V, Saari E, Itkonen, et al. Changes in health-related quality of life and functioning ability in help-seeking adolescents and adolescents at heightened risk of developing psychosis during family- and community-oriented intervention model. Int J Psychiatry Clin Pract. 2013;17(4):253-8.114.

114. Grano N, Karjalainen M, Edlund V, Saari E, Itkonen A, et al. Health-related quality of life among adolescents: a comparison between subjects at risk for psychosis and other help seekers. Early Interv Psychiatry. 2014;8(2):163-9.

115. Grant J, Cranston A, Horsman J, Furlong W, Barr N, et al. Health status and health-related quality of life in adolescent survivors of cancer in childhood. J Adolesc Health. 2006;38(5):504-10.

116. Gray R, Petrou S, Hockley C, Gardner F. Self-reported health status and health-related quality of life of teenagers who were born before 29 weeks' gestational age. Pediatrics. 2007;120(1):e86-93.

117. Greenough A, Peacock J, Zivanovic S, Alcazar-Paris M, Lo J, et al. United Kingdom Oscillation Study: long-term outcomes of a randomised trial of two modes of neonatal ventilation. Health Technol Assess. 2014;18(41):v-xx,1-95.

118. Gulati S, Madsbu MA, Solberg TK, Sorlie A, Giannadakis C, et al. Lumbar microdiscectomy for sciatica in adolescents: a multicentre observational registry-based study. Acta Neurochir 2017;159(3):509-16.

119. Gundle KR, Punt SE, Mattioli-Lewis T, Conrad EU. Can a made-for-consumer activity monitor assess physical activity in adolescents and young adults after lower extremity limb salvage for osseous tumors? J Pediatr Orthop 2017;37(3):e192-6.

120. Haapamaki J, Roine RP, Sintonen H, Kolho K. Health-related quality of life in paediatric patients with inflammatory bowel disease related to disease activity. J Paediatr Child Health. 2011;47(11):832-7.

121. Haavisto A, Korkman M, Sintonen H, Holmberg C, Jalanko H, et al. Risk factors for impaired quality of life and psychosocial adjustment after pediatric heart, kidney, and liver transplantation. Pediatr Transplant. 2013;17(3):256-65.122.

122. Hailer YD, Haag AC, Nilsson O. Legg-Calve-perthes disease: quality of life, physical activity, and behavior pattern. J Pediatr Orthop. 2014;34(5):514-21.

123. Hakkaart-van Roijen L, Goettsch WG, Ekkebus M, Gerretsen P, Stolk EA. The cost-effectiveness of an intensive treatment protocol for severe dyslexia in children. Dyslexia. 2011;17(3):256-67.

124. Han PP, Holbrook TL, Sise MJ, Sack DI, Sise CB, et al. Postinjury depression is a serious complication in adolescents after major trauma: injury severity and injury-event factors predict depression and long-term quality of life deficits. J Trauma. 2011;70(4):923-30.

125. Hanberger L, Ludvigsson J, Nordfeldt S. Health-related quality of life in intensively treated young patients with type 1 diabetes. Pediatr Diabetes. 2009;10(6):374-81.

126. Hartman JD, Craig BM, Blackburn C, Simmons V. The association between maternal smoking during pregnancy and child quality-adjusted life years. Value Health 2016;19(3):A117.

127. Hatam N, Shirvani S, Javanbakht M, Askarian M, Rastegar M. Cost-utility analysis of neonatal screening program, Shiraz University of Medical Sciences, Shiraz, Iran, 2010. Iranian J Pediatri. 2013;23(5):493-500.

128. Hernandez HG, Avila M, Garin O, Pont A, Ferrer M. Validity of the EQ-5D-Youth in asthmatic children. J Thoracic Dis. 2016;8.

129. Hinds PS, Burghen EA, Zhou Y, Zhang L, West N, et al. The Health Utilities Index 3 invalidated when completed by nurses for pediatric oncology patients. Cancer Nurs. 2007;30(3):169-77.130.

130. Hodgkins P, Setyawan J, Banaschewski T, Soutullo C, Lecendreux M, et al. Health utility scores in children and adolescents with attention-deficit/hyperactivity disorder: Response to stimulant treatment. Eur Child Adolesc Psychiatry. 2013;22(2 SUPPL.1):S127.131.

131. Hoffmann T, Iturriza M, Faaborg-Andersen J, Kraaer C, Nielsen CP, et al. Prospective study of the burden of rotavirus gastroenteritis in Danish children and their families. Eur J Pediatr. 2011;170(12):1535-39.

132. Hogan ME, Taddio A, Katz J, Shah V, Krahn M. Health utilities in people with chronic pain using a population-level survey and linked health care administrative data. Pain 2017;158(3):408-16.

133. Holbrook TL, Hoyt DB, Coimbra R, Potenza B, Sise M, et al. High rates of acute stress disorder impact quality-of-life outcomes in injured adolescents: mechanism and gender predict acute stress disorder risk. J Trauma. 2005;59(5):1126-30.

134. Holbrook TL, Hoyt DB, Coimbra R, Potenza B, Sise M, et al. Trauma in adolescents causes long-term marked deficits in quality of life: adolescent children do not recover preinjury quality of life or function up to two years postinjury compared to national norms. J Trauma. 2007;62(3):577-83.135.

135. Horsman JR, Shimoda S, Furlong W, Barr RD, de Camargo B. Disability and health-related quality of life in long-term survivors of cancer in childhood in Brazil: An assessment of the construct validity of the health utilities index (HUI3). Value Health. 2008;11(3):A75-A76.

136. Huppertz H, Forster J, Heininger U, Roos R, Neumann H, et al. The parental appraisal of the morbidity of diarrhea in infants and toddlers (PAMODI) survey. Clin Pediatr. 2008;47(4):363-71.

137. Iskrov GG, Stefanov RS, Lopez-Bastida J, Linertova R, Oliva-Moreno J, et al. Economic burden and health-related quality of life of patients with cystic fibrosis in Bulgaria. Folia Med (Plovdiv). 2015;57(1):56-64.

138. Janse AJ, Uiterwaal CSPM, Gemke RJBJ, Kimpen JLL, Sinnema G. A difference in perception of quality of life in chronically ill children was found between parents and pediatricians. J Clin Epidemiol. 2005;58(5):495-502.

139. Janssens L, Gorter JW, Ketelaar M, Kramer WLM, Holtslag HR. Long-Term Health-Related Quality of Life in Major Pediatric Trauma: A Pilot Study. Eur J Trauma Emerg Surg. 2009;35(4):371-7.

140. Jelsma J. A comparison of the performance of the EQ-5D and the EQ-5D-Y health-related quality of life instruments in South African children. Int J Rehabil Res. 2010;33(2):172-7.

141. Jelsma J, Ramma L. How do children at special schools and their parents perceive their HRQoL compared to children at open schools? Health Qual Life Outcomes. 2010;8:72-8.

142. Jog M, Wein T, Bhogal M, Dhani S, Miller R, et al. Real-world, long-term quality of life following therapeutic OnabotulinumtoxinA treatment. Can J Neurol Sci 2016;43(5):687-96.

143. Joosten K, van der Velde K, Joosten P, Rutten H, Hulst J, et al. Association between nutritional status and subjective health status in chronically ill children attending special schools. Qual Life Res. 2016;25:969-77.

144. Juniper EF, Guyatt GH, Feeny DH, Griffith LE, Ferrie PJ. Minimum skills required by children to complete health-related quality of life instruments for asthma: comparison of measurement properties. Eur Respir J. 1997;10:2285-94.

145. Kang E. Validity of child health-6 dimension (CH-6D) for adolescents. Value Health. 2016;19(7):A854.146.

146. Keating CL, Moodie ML, Richardson J, Swinburn BA. Utility-based quality of life of overweight and obese adolescents. Value Health. 2011;14(5):752-8.

147. Kennedy CR, Leyland K, Comparison of screening instruments for disability and emotional/behavioral disorders with a generic measure of health-related quality of life in survivors of childhood brain tumors. Int J Cancer Suppl. 1999;12:106-11.

148. Kennedy ITR, van Hoek AJ, Ribeiro S, Christensen H, Edmunds WJ, et al. Short-term changes in the health state of children with group B meningococcal disease: A prospective, national cohort study. PLoS One 2017;12(5)

149. Kesztyus D, Schreiber A, Kobel S, Wartha O, Kesztyus T, et al. Illness and determinants of health-related quality of life in a cross-sectional sample of schoolchildren in different weight categories. Ger Med Sci. 2014;12.

150. Kim SK, Jo MW, Kim SH. A cross sectional survey on health-related quality of life of elementary school students using the Korean version of the EQ-5D-Y. PeerJ 2017;5:e3115.

151. Klaassen RJ, Krahn M, Gaboury I, Hughes J, Anderson R, et al. Evaluating the ability to detect change of health‐related quality of life in children with Hodgkin disease. Cancer. 2010;116(6):1608-14.152.

152. Knapp C, Madden V, Revicki D, Feeny D, Wang H, et al. Health status and health-related quality of life in a pediatric palliative care program. J Palliat Med. 2012;15(7):790-7.153.

153. Kodra Y, Cavazza M, Schieppati A, de Santis M, Armeni P, et al. The social burden and quality of life of patients with haemophilia in Italy. Blood Transfus. 2014;12(Suppl3):s567-75.

154. Koomen I, Raat H, Jennekens-Schinkel A, Grobbee DE, Roord JJ, et al. Academic and behavioral limitations and health-related quality of life in school-age survivors of bacterial meningitis. Qual Life Res. 2005;14(6):1563-72.

155. Kotwicki RJ, Condra L, Vermeulen L, Wolf T, Douglas J, et al. Assessing the quality of life in children with cystic fibrosis. Wisconsin Med J. 2001;100(5):50-4.

156. Kramer MS, Etezadi-Amoli J, Ciampi A, Tange SM, Drummond KN, et al. Parents' versus physicians' values for clinical outcomes in young febrile children. Pediatrics. 1994;93(5):697-702.

157. Kulkarni AV, Cochrane DD, McNeely PD, Shams I. Comparing children's and parents' perspectives of health outcome in paediatric hydrocephalus. Dev Med Child Neurol. 2008;50(8):587-92.

158. Kulkarni AV, Cochrane DD, McNeely PD, Shams I. Medical, social, and economic factors associated with health-related quality of life in Canadian children with hydrocephalus. J Pediatr. 2008;153(5):689-95.

159. Kulkarni AV, Hui S, Shams I, Donnelly R. Quality of life in obstructive hydrocephalus: endoscopic third ventriculostomy compared to cerebrospinal fluid shunt. Childs Nerv Syst. 2010;26(1):75-9.

160. Kulkarni AV, Shams I, Cochrane DD, McNeely PD. Quality of life after endoscopic third ventriculostomy and cerebrospinal fluid shunting: an adjusted multivariable analysis in a large cohort. J Neurosurg Pediatr. 2010;6(1):11-16.

161. Kulpeng W, Sornsrivichai V, Chongsuvivatwong V, Rattanavipapong W, Leelahavarong P, et al. Variation of health-related quality of life assessed by caregivers and patients affected by severe childhood infections. BMC Pediatr. 2013;13:122-30.

162. Ladner TR, Westrick AC, Wellons JC, Shannon CN. Health-related quality of life in pediatric Chiari Type I malformation: the Chiari Health Index for Pediatrics. J Neurosurg Pediatr. 2016;17(1):76-85.

163. Laitakari E, Koljonen V, Pyorala S, Rintala R, Roine RP, et al. The long-term health-related quality of life in children treated for burns as infants 5-9 years earlier. Burns. 2015;41(6):1186-92.

164. Landfeldt E, Lindgren P, Bell CF, Guglieri M, Straub V, et al. Health-related quality of life in patients with Duchenne muscular dystrophy: a multinational, cross-sectional study. Dev Med Child Neurol. 2015

165. Lee GM, Salomon JA, LeBaron CW, Lieu TA. Health-state valuations for pertussis: methods for valuing short-term health states. Health Qual Life Outcomes. 2005;3:17-30.

166. Lee JM, Rhee K, O’Grady MJ, Basu A, Winn A, et al. Health utilities for children and adults with type 1 diabetes. Med Care. 2011;49(10):924-31.

167. Lee D, Gladwell D, Batty AJ, Brereton N, Tate E. The cost effectiveness of licensed oromucosal midazolam (Buccolam(R)) for the treatment of children experiencing acute epileptic seizures: an approach when trial evidence is limited. Paediatric Drugs. 2013;15(2):151-62.

168. Lenhard F, Ssegonja R, Andersson E, Feldman I, Ruck C, et al. Cost-effectiveness of therapist-guided internet-delivered cognitive behaviour therapy for paediatric obsessive-compulsive disorder: results from a randomised controlled trial. BMJ Open 2017;7(5):e015246.

169. Lin K, Yang X, Yin G, Lin S. Diabetes self-care activities and health-related quality-of-life of individuals with type I diabetes mellitus in Shantou, China. J Int Med Res 2016;44(1):147-56.

170. Liu H, Liu HX, Kang HY, Gu Z, Hong SL. Evaluation on health-related quality of life in deaf children with cochlear implant in China. Int J Pediatr Otorhinolaryngol 2016;88:136-41.

171. Lloyd A, Swinburn P, Boye KS, Curtis B, Sarpong E, et al. A valuation of infusion therapy to preserve islet function in type 1 diabetes. Value Health. 2010;13(5):636-42.

172. Lloyd A, Hogkins P, Sasane R, Akehurst R, Sonuga-Barke EJS, et al. Estimation of utilities in Attention-Deficit Hyperactivity Disorder for economic evaluations. Patient. 2011;4(4):247-57.173.

173. Lloyd A, Gallop K, Hutchings A, Acaster S. How do we estimate quality adjusted life years (QALYs) in rare diseases? A case study in Hypophosphatasia. Value Health. 2015;18(7):A651.

174. Lock C, Wilson J, Steen N, Eccles M, Mason H, et al. North of England and Scotland Study of Tonsillectomy and Adeno-tonsillectomy in Children (NESSTAC): a pragmatic randomised controlled trial with a parallel non-randomised preference study. Health Technol Assess. 2010;14(13):iii-iv,1-164.

175. Lovett RES, Kitterick PT, Hewitt CE, Summerfield AQ. Bilateral or unilateral cochlear implantation for deaf children: an observational study. Arch Dis Child. 2010;95(2):107-12.

176. Lynch FL, Hornbrook M, Clarke GN, Perrin N, Polen MR, et al. Cost-effectiveness of an intervention to prevent depression in at-risk teens. Arch Gen Psychiatry. 2005;62:1241-48.

177. Lynch FL, Dickerson JF, Feeny DH, Clarke GN, MacMillan AL. Measuring health-related quality of life in teens with and without depression. Med Care 2016;54(12):1089-97.

178. Maia CR, Stella CF, Wagner F, Pianca TG, Krieger FV, et al. Cost-utility analysis of methylphenidate treatment for children and adolescents with ADHD in Brazil. Rev Bras Psiquiatr. 2016;38(1):30-38.

179. Makkes S, Renders CM, Bosmans JE, van der Baan-Slootweg OH, Seidell JC. Cardiometabolic risk factors and quality of life in severely obese children and adolescents in the Netherlands. BMC Pediatr. 2013;13:62-70.180.

180. Malik BA, Gibbons K, Spady D, Lees G, Otley A, et al. Health-related quality of life in pediatric ulcerative colitis patients on conventional medical treatment compared to those after restorative proctocolectomy. Int J Colorectal Dis. 2013;28(3):325-33.

181. Marlow. R, Finn A, Trotter C. Quality of life impacts from rotavirus gastroenteritis on children and their families in the UK. Vaccine. 2015;33(39):5212-6.

182. Martin A, Cottrell S, Standaert B. Estimating utility scores in young children with acute rotavirus gastroenteritis in the UK. J Med Econ. 2008;11(3):471-84.

183. Matza LS, Secnik K, Mannix S, Sallee FR. Parent-proxy EQ-5D ratings of children with attention-deficit hyperactivity disorder in the US and the UK. PharmacoEcon. 2005;23(8):777-90.

184. Matza LS, Secnik K, Rentz AM, Mannix S, Sallee FR, et al. Assessment of health state utilities for attention-deficit/hyperactivity disorder in children using parent proxy report. Qual Life Res. 2005;14(3):735-47.

185. McBain RK, Salhi C, Hann K, Salomon JA, Kim JJ, et al. Costs and cost-effectiveness of a mental health intervention for war-affected young persons: decision analysis based on a randomized controlled trial. Health Policy Plan 2016;31(4):415-24.

186. Midgley DE, Bradley TA, Donohoe C, Kent KP, Alonso EM. Health-related quality of life in long-term survivors of pediatric liver transplantation. Liver Transpl. 2000;6(3):333-9.

187. Miller T, Hallfors D, Cho H, Luseno W, Waehrer G. Cost-effectiveness of school support for orphan girls to prevent HIV infection in Zimbabwe. Prev Sci. 2013;14(5):503-12.

188. Miller TR, Steinbeigle R, Wicks A, Lawrence BA, Barr M, et al. Disability-adjusted life-year burden of abusive head trauma at ages 0-4. Pediatrics. 2014;134(6):e1545-50.

189. Mittmann N, [Trakas K](https://www.ncbi.nlm.nih.gov/pubmed/?term=Trakas%20K%5BAuthor%5D&cauthor=true&cauthor_uid=10537955), [Risebrough N](https://www.ncbi.nlm.nih.gov/pubmed/?term=Risebrough%20N%5BAuthor%5D&cauthor=true&cauthor_uid=10537955), [Liu BA](https://www.ncbi.nlm.nih.gov/pubmed/?term=Liu%20BA%5BAuthor%5D&cauthor=true&cauthor_uid=10537955). Utility scores for chronic conditions in a community-dwelling population. Pharmacoeconomics. 1999;15(4):369-76.

190. Mort S, Salantera S, Matomaki J, Salmi TT, Lahteenmaki PM. Self-reported health-related quality of life of children and adolescent survivors of extracranial childhood malignancies: a Finnish nationwide survey. Qual Life Res. 2011;20(5):787-97.

191. Mpundu-Kaambwa C, Chen G, Russo R, Stevens K, Petersen KD, et al. Mapping CHU9D utility scores from the PedsQLTM 4.0 SF-15. Pharmacoeconomics 2017;35(4):453-67.

192. Mulhern RK. Correlation of the Health Utilities Index Mark 2 cognition scale and neuropsychological functioning among survivors of childhood medulloblastoma. Int J Cancer Suppl. 1999;12:91-4.

193. Munzenberger PJ, van Wagnen CA, Abdulhamid I, Walker PC. Quality of life as a treatment outcome in patients with cystic fibrosis. Pharmacotherapy. 1999;19(4):393-8.

194. Murillo M, Bel J, Perez J, Corripio R, Carreras G, et al. Health-related quality of life (HRQOL) and its associated factors in children with Type 1 Diabetes Mellitus (T1DM). BMC Pediatr 2017;17(1):16.

195. Naraine VS, Risebrough NA, Oh P, Blanchette VS, Lee S, et al. Health-related quality-of-life treatments for severe haemophilia: utility measurements using the Standard Gamble technique. Haemophilia. 2002;8(2):112-20.

196. Neufeld EJ, Recht M, Sabio H, Saxena K, Solem CT, et al. Effect of acute bleeding on daily quality of life assessments in patients with congenital Hemophilia with inhibitors and their families: observations from the dosing observational study in Hemophilia. Value Health. 2012;15(6):916-25.197.

197. Nokso-Koivisto J, Blomgren K, Roine RP, Sintonen H, Pitkaranta A. Impact of tonsillectomy on health-related quality of life and healthcare costs in children and adolescents. Int J Pediatr Otorhinolaryngol. 2014;78(9):1508-12.

198. Nordyke K, Norstrom F, Lindholm L, Carlsson A, Danielsson L, et al. Health-related quality-of-life in children with coeliac disease, measured prior to receiving their diagnosis through screening. J Med Screen. 2011;18(4):187-92.

199. Nordyke K, Norstrom F, Lindholm L, Stenlund H, Rosen A, et al. Health-related quality of life in adolescents with screening-detected celiac disease, before and one year after diagnosis and initiation of gluten-free diet, a prospective nested case-referent study. BMC Public Health. 2013;13:142-8.

200. Nwachukwu BU, So C, Schairer WW, Shubin-Stein BE, Strickland SM, et al. Economic decision model for first-time traumatic patellar dislocations in adolescents. Am J Sports Med 2017;20(10).

201. Oh PI, Maerov P, Pritchard D, Knowles SR, Einarson TR, et al. A cost-utility analysis of second-line antibiotics in the treatment of acute otitis media in children. Clin Ther. 1996;18(1):160-82.

202. Oostenbrink R, Moll HA, Essink-Bot ML. The EQ-5D and the Health Utilities Index for permanent sequelae after meningitis: a head-to-head comparison. J Clin Epidemiol. 2002;55:791-99.203.

203. Orenstein DM, Pattishall EN, Nixon PA, Ross EA, Kaplan RM. Quality of well-being before and after antibiotic treatment of pulmonary exacerbation in patients with cystic fibrosis. Chest. 1990;98(5):1081-84.

204. Peasgood T, Bhardwaj A, Biggs K, Brazier JE, Coghill D, et al. The impact of ADHD on the health and well-being of ADHD children and their siblings. Eur Child Adolesc Psychiatry 2016;25(11):1217-31.

205. Peetsold MG, Huisman J, Hofman VE, Heij HA, Raat H, et al. Psychological outcome and quality of life in children born with congenital diaphragmatic hernia. Arch Dis Child. 2009;94(11):834-40.

206. Penn A, Lowis SP, Stevens MCG, Shortman RI, Hunt LP, et al. A detailed prospective longitudinal assessment of health status in children with brain tumors in the first year after diagnosis. J Pediatr Hematol Oncol. 2011;33(8):592-9.207.

207. Péntek M, Baji P, Pogany G, Brodszky V, Boncz I, et al. Health related quality of life of patients and their caregivers in rare diseases results of the Burqol-Rd project in Hungary. Value Health. 2014;17(7): A538.

208. Petersen KD, Chen G, Mpundu-Kaambwa C, Stevens K, Brazier J, et al. Measuring health-related quality of life in adolescent populations: An empirical comparison of the CHU9D and the PedsQLTM 4.0 Short Form 15. Patient 2017.

209. Petracci E, Cavrini G. The effect of weight status, lifestyle, and body image perception on health-related quality of life in children: a quantile approach. Qual Life Res. 2013;22(9):2607-15.

210. Petrou S, McCann D, Law CM, Watkin PM, Worsfold S, et al. Health status and health-related quality of life preference-based outcomes of children who are aged 7 to 9 years and have bilateral permanent childhood hearing impairment. Pediatrics. 2007;120(5):044-52.211.

211. Petrou S, AbangmaG, Johnson S, Wolke D, Marlow N. *Costs and health utilities associated with extremely preterm birth: evidence from the EPICure study.* Value Health. 2009;12(8):1124-34.

212. Petrou S, Dakin H, Abangma G, Benge S, Williamson I. Cost-utility analysis of topical intranasal steroids for otitis media with effusion based on evidence from the GNOME trial. Value Health. 2010;13(5):543-51.

213. Petrou S, Johnson S, Wolke D, Hollis C, Kochhar P, et al. Economic costs and preference-based health-related quality of life outcomes associated with childhood psychiatric disorders. Br J Psychiatry. 2010;197(5):395-404.

214. Petrou S, Johnson S, Wolke D, Marlow N. The association between neurodevelopmental disability and economic outcomes during mid-childhood. Child Care Health Dev. 2011;39(3):345-57.

215. Petrou S, Kupek E. Estimating preference-based health utilities index mark 3 utility scores for childhood conditions in England and Scotland. Med Decis Making. 2009;29(3):291-303.

216. Philipsson A, Duberg A, Moller M, Hagberg L. Cost-utility analysis of a dance intervention for adolescent girls with internalizing problems*.* Cost Eff Resour Alloc. 2013;11(1):4.

217. Pitt M, Garside R, Stein K. A cost-utility analysis of pimecrolimus vs. topical corticosteroids and emollients for the treatment of mild and moderate atopic eczema*.* Br J Dermatol. 2006;154(6):1137-46.

218. Poenaru D, Pemberton J, Frankfurter C, Cameron BH, Stolk E. Establishing disability weights for congenital pediatric surgical conditions: a multi-modal approach. Popul Health Metr 2017;15(1):8.

219. Pogany L, Barr RD, Shaw A, Speechley KN, Barrera M, et al. Health status in survivors of cancer in childhood and adolescence. Qual Life Res. 2006;15(1):143-157.

220. Poley MJ, Stolk EA, Langemeijer RA, Molenaar JC, Busschbach JJ. The cost-effectiveness of neonatal surgery and subsequent treatment for congenital anorectal malformations. J Pediatr Surg. 2001;36(10):1471-1478.221.

221. Poley MJ, Stolk EA, Tibboel D, Molenaar JC, Busschbach JJ. The cost-effectiveness of treatment for congenital diaphragmatic hernia. J Pediatr Surg. 2002;37(9):1245-1252.

222. Polinder S, Meerding WJ, Toet H, Mulder S, Essink-Bot ML, van Beech EF. Prevalence and prognostic factors of disability after childhood injury*.* Pediatrics. 2005;116(6):e810-e817.

223. Portwine C, Rae C, Davis J, Teira P, Schechter T, et al. Health-related quality of life in survivors of high-risk neuroblastoma after stem cell transplant: A national population-based perspective. Pediatr Blood Cancer 2016;63(9):1615-21.

224. Powell CV, Kolamunnage-Dona R, Lowe J, Boland A, Petrou S, et al. MAGNEsium Trial In Children (MAGNETIC): a randomised, placebo-controlled trial and economic evaluation of nebulised magnesium sulphate in acute severe asthma in children. Health Technol Assess. 2013;17(45):v-vi, 1-216.

225. Prince FH, Geerdink LM, Borsboom GJ, Twilt M, van Rossum MA, et al. Major improvements in health-related quality of life during the use of etanercept in patients with previously refractory juvenile idiopathic arthritis. Ann Rheum Dis. 2010;69(1):138-42.226.

226. Protudjer JL, Jansson SA, Ostblom E, Arnlind MH, Bengtsson U, et al. Health-related quality of life in children with objectively diagnosed staple food allergy assessed with a disease-specific questionnaire. Acta Paediatr. 2015;104(10):1047-54.

227. Quinn GE, Dobson V, Saigal S, Phelps DL, Hardy RJ, et al. Health-related quality of life at age 10 years in very low-birth-weight children with and without threshold retinopathy of prematurity. Arch Ophthalmol. 2004;122(11):1659-66.

228. Qvist E, Narhi V, Apajasalo M, Ronnholm K, Jalanko H, et al. Psychosocial adjustment and quality of life after renal transplantation in early childhood. Pediatr Transplant. 2004;8(2):120-5.

229. Raat H, Bonsel GJ, Essink-Bot ML, Landgraf JM, Gemke RJ. Reliability and validity of comprehensive health status measures in children: The Child Health Questionnaire in relation to the Health Utilities Index. J Clin Epidemiol. 2002; 55(1):67-76.

230. Raat H, Bonsel GJ, Hoogeveen WC, Essink-Bot ML, Dutch HUI Group. Feasibility and reliability of a mailed questionnaire to obtain visual analogue scale valuations for health states defined by the Health Utilities Index Mark 3. Med care. 2004;42(1):13-18.

231. Rae C, Furlong W, de Pauw S, Barr RD, Gelber RD, et al. PR1 Variability of Health Utilities Index Mark 3 (HUI3) measurements during treatment for acute lymphoblastic leukemia in childhood. Value Health. 2008;11(3):A25.

232. Rae C, Horsman JR, Furlong W, Silverman LB, Sallan SE, et al. PCN110 Differences in Health-Related Quality of Life Between Children Treated for Acute Lymphoblastic Leukemia (ALL) on Dana Farber Cancer Institute (DFCI) Protocols. Value Health. 2012;15(7):A429.

233. Rae C, Furlong W, Jankovic M, Moghrabi A, Naqvi A, et al. Economic evaluation of treatment for acute lymphoblastic leukaemia in childhood. Eur J Cancer Care. 2014;23(6):779-785.

234. Rae C, Benger A, Fowler JA, MacEachern J, Pullenayegum E, et al. Need and use of healthcare services in survivors of Hodgkin Lymphoma. J Adolesc Young Adult Oncol 2016;5(2):174-80.

235. Raluy-Callado M, Chen MH, Whiteman DA, Fang J, Wiklund I. The impact of Hunter syndrome (mucopolysaccharidosis type II) on health-related quality of life. Orphanet J Rare Dis. 2013;8:101.

236. Raspa M, Sacco P, Candrilli SD, Bishop E, Petrillo J. Validity of a condition specific outcome measure for fragile X syndrome: the Aberrant Behaviour Checklist-utility index. J Intellect Disabil Res 2016;60(9):844-55.

237. Ratcliffe J, Stevens K, Flynn T, Brazier J, Sawyer M. *An assessment of the construct validity of the CHU9D in the Australian adolescent general population.* Qual Life Res. 2012;21(4):717-25.

238. Ratcliffe J, Stevens K, Flynn T, Brazier J, Sawyer MG. Whose values in health? An empirical comparison of the application of adolescent and adult values for the CHU-9D and AQOL-6D in the Australian adolescent general population*.* Value Health. 2012;15(5):730-736.239.

239. Rautava L, Hakkinen U, Korvenranta E, Andersson S, Gissier M, et al. Health-Related Quality of Life in 5-Year-Old Very Low Birth Weight Infants*.* J Pediatr. 2009;155(3):338.

240. Redouane B, Cohen E, Stephens D, Keilty K, Mouzaki M, et al. Parental perceptions of quality of life in children on long-term ventilation at home as compared to enterostomy tubes. PLoS One 2016;11(2):e0149999.

241. Renier CM, Gerberich SG, McGovern PM, Alexander BH, Church TR, et al. Health Utilities Index-3 Among Injured Youth Residing in Agricultural Households: Regional Rural Injury Study-III (RRIS-III)(PP). Qual Life Res. 2012;20:100-100.

242. Rhodes ET, Prosser LA, Lieu TA, Songer TJ, Ludwig DS, et al. Preferences for type 2 diabetes health states among adolescents with or at risk of type 2 diabetes mellitus*.* Pediatr Diabetes. 2011,12(8):724-32.243.

243. Rhodes ET, Goran MI, Lieu TA, Lustig RH, Prosser LA, et al. Health-related quality of life in adolescents with or at risk for type 2 diabetes mellitus. J Pediatr. 2012; 160(6):911-7.

244. Roberts G, Burnett AC, Lee KJ, Cheong J, Wood SJ, et al. Quality of life at age 18 years after extremely preterm birth in the post-surfactant era*.* J Pediatr. 2013;163(4):1008-13 e1.

245. Robertson W, Fleming J, Kamal A, Hamborg T, Khan KA. Randomised controlled trial and economic evaluation of the 'Families for Health' programme to reduce obesity in children. Arch Dis Child 2017;102(5):416-26.

246. Robles N, Rajmil L, Rodriguez-Arjona D, Azuara M, Codina F, et al. Development of the web-based Spanish and Catalan versions of the Euroqol 5D-Y (EQ-5D-Y) and comparison of results with the paper version. Health Qual Life Outcomes 2015;13(1):72.

247. Rochanathimoke O, Postma M, Thavorncharoensap M, Riewpaiboon A, Thinyounyong W. Quality Of Life Of Diarrheal Children And Caregivers In Thailand. Value Health. 2014;17(7):A368-A369.

248. Rocque BG, Bishop ER, Scogin MA, Hopson BD, Arynchyna AA, et al. Assessing health-related quality of life in children with spina bifida*.* J Neurosurg Pediatr. 2015;15(2):144-9.249.

249. Rodriguez-Martinez CE, Sossa-Briceno MP, Castro-Rodriguez JA. Cost-utility analysis of the inhaled steroids available in a developing country for the management of pediatric patients with persistent asthma. J Asthma. 2013;50(4):410-8.

250. Roman JD. Adolescent endometriosis in the Waikato region of New Zealand--a comparative cohort study with a mean follow-up time of 2.6 years. Aust N Z J Obstet Gynaecol. 2010;50(2):179-83.

251. Roposch A, Liu LQ, Offiah AC, Wedge JH. Functional outcomes in children with osteonecrosis secondary to treatment of developmental dysplasia of the hip*.* J Bone Joint Surg Am. 2011;93(24):e145.

252. Rosenbaum PL, Livingston MH, Palisano RJ, Galuppi BE, Russell DJ. Quality of life and health-related quality of life of adolescents with cerebral palsy. Dev Med Child Neurol. 2007;49(7):516-21.

253. Rowley PT, Loader S, Kaplan RM. Prenatal screening for cystic fibrosis carriers: an economic evaluation. Am J Hum Genet. 1998;63(4):1160-74.

254. Sach TH, Barton GR. Interpreting parental proxy reports of (health-related) quality of life for children with unilateral cochlear implants*.* Int J Pediatr Otorhinolaryngol. 2007;71(3):435-45.

255. Saigal S, Feeny D, Furlong W, Rosenbaum P, Burrows E, et al. Comparison of the health-related quality of life of extremely low birth weight children and a-reference group of children at age eight years. J Pediatr. 1994;125(3):418-425.

256. Saigal S, Feeny D, Rosenbaum P, Furlong W, Burrows E, et al. Self-perceived health status and health-related quality of life of extremely low-birth-weight infants at adolescence. JAMA. 1996;276(6):453-9.

257. Saigal S, Stoskopf BL, Feeny D, Furlong W, Burrows E, et al. Differences in preferences for neonatal outcomes among health care professionals, parents, and adolescents. JAMA. 1999;281(21):1991-7.

258. Saigal S, Rosenbaum PL, Feeny D, Burrows E, Furlong W, et al. Parental perspectives of the health status and health-related quality of life of teen-aged children who were extremely low birth weight and term controls. Pediatrics. 2000;105(3 Pt 1):569-74.

259. Saigal S, Stoskopf BL, Burrows E, Streiner DL, Rosenbaum PL. Stability of maternal preferences for pediatric health states in the perinatal period and 1 year later. Arch Pediatr Adolesc Med. 2003;157(3):261-9.

260. Saigal S, Ferro MA, van Lieshout RJ, Schmidt LA, Morrison KM, et al. Health-related quality of life trajectories of extremely low birth weight survivors into adulthood. J Pediatr 2016;179:68-73.

261. Saw SM, Gazzard G, Au Eong KG, Koh D. Utility values and myopia in teenage school students. Br J Ophthalmol. 2003;87(3):341-345.

262. Scalone L, Cavrini G, Broccoli S, Selleri P. PMC15 Convergent, discriminant, concurrent validity and reliability of the EQ-5D (Child): Results. Value Health. 2007;10(6):A455.263.

263. Scalone L, Tomasetto C, Matteucci MC, Selleri P, Broccoli S, et al. Assessing quality of life in children and adolescents: development and validation of the Italian version of the EQ-5D-Y*.* Ital J Public Health. 2012;8(4).

264. Scott D, Ferguson GD, Jelsma J. The use of the EQ-5D-Y health related quality of life outcome measure in children in the Western Cape, South Africa: psychometric properties, feasibility and usefulness - a longitudinal, analytical study. Health Qual Life Outcomes 2017;15(1):12.

265. Secnik K, Matza LS, Cottrell S, Edgell E, Tilden D, et al. Health state utilities for childhood attention-deficit/hyperactivity disorder based on parent preferences in the United kingdom. Med Decis Making. 2005;25(1):56-70.

266. Selvadurai HC, Blimkie CJ, Meyers N, Mellis CM, Cooper PJ, et al. Randomized controlled study of in-hospital exercise training programs in children with cystic fibrosis. Pediatr Pulmonol. 2002; 33(3):194-200.

267. Semenov YR, Yeh ST, Seshamani M, Wang NY, Tobey EA, et al. Age-dependent cost-utility of pediatric cochlear implantation*.* Ear Hear. 2013;34(4):402-12.

268. Seyedifar M, Dorkoosh FA, Hamidieh AA, Naderi M, Karami H, et al. Health-related quality of life and health utility values in Beta Thalassemia Major patients receiving different types of iron chelators in Iran. Int J Hematol Oncol Stem Cell Res 2016;10(4):224-31.

269. Sierwald I, John MT, Sagheri D, Neuschulz J, Schuler E, et al. The German 19-item version of the Child Oral Health Impact Profile: translation and psychometric properties. Clin Oral Investig 2016;20(2):301-13.

270. Simonova O, Gorinova Y, Vinyarskaya I, Samsonova M, Chernikov V. Dynamics of Quality of Life in Children with Cystic Fibrosis Aged 5-16 Years. Value Health. 2013;16(7):A625.

271. Sims-Williams HJ, Sims-Williams HP, Mbabazi Kabachelor E, Warf BC. Quality of life among children with spina bifida in Uganda. Arch Dis Child 2017;0:1.

272. Smith-Olinde L, Grosse SD, Olinde F, Martin PF, Tilford JM. Health state preference scores for children with permanent childhood hearing loss: a comparative analysis of the QWB and HUI3. Qual Life Res. 2008;17(6):943-53.

273. Soucie JM, Grosse SD, Siddiqi AEA, Byams V, Thierry J, et al. The effects of joint disease, inhibitors and other complications on health-related quality of life among males with severe haemophilia A in the United States. Haemophilia 2017;e287-93.

274. Sparreboom M, Snik AF, Mylanus EA. Sequential bilateral cochlear implantation in children: quality of life. Arch Otolaryngol Head Neck Surg. 2012;138(2):134-41.

275. Sruamsiri R, Chaiyakunapruk N, Pakakasama S, Sirireung S, Sripaiboonkij N, et al. Cost utility analysis of reduced intensity hematopoietic stem cell transplantation in adolescence and young adult with severe thalassemia compared to hypertransfusion and iron chelation program*.* BMC Health Serv Res. 2013;13(1):1.

276. Stade BC, Stevens B, Ungar WJ, Beyene J, Koren G. Health-related quality of life of Canadian children and youth prenatally exposed to alcohol. Health Qual Life Outcomes. 2006;4:no pagination.

277. Stevens KJ, Freeman JV. An assessment of the psychometric performance of the Health Utilities Index 2 and 3 in children following discharge from a U.K. pediatric intensive care unit. Pediatr Crit Care Med. 2012; 13(4):387-92.

278. Stevens K, Ratcliffe J. Measuring and valuing health benefits for economic evaluation in adolescence: an assessment of the practicality and validity of the child health utility 9D in the Australian adolescent population. Value Health. 2012;15(8):1092-9.

279. Stolk EA, Busschbach JJ, Vogels T. Performance of the EuroQol in children with imperforate anus. Qual Life Res. 2000;9(1):29-38.

280. Stromqvist F, Stromqvist B, Jonsson B, Gerdhem P, Karlsson MK. Predictive outcome factors in the young patient treated with lumbar disc herniation surgery. J Neurosurg Spine 2016;25(4):448-55.

281. Sung L, Greenberg ML, Doyle JJ, Young NL, Ingber S, et al. Construct validation of the Health Utilities Index and the Child Health Questionnaire in children undergoing cancer chemotherapy. Br J Cancer. 2003;88(8):1185-90.

282. Sung L, Greenberg ML, Young NL, McLimont M, Ingber S, et al. Validity of a modified standard gamble elicited from parents of a hospital-based cohort of children. J clin epidemiol. 2003;56(9):848-855.

283. Sung L, Young NL, Greenberg ML, McLimont M, Samanta T, et al. Health-related quality of life (HRQL) scores reported from parents and their children with chronic illness differed depending on utility elicitation method. J Clin Epidemiol. 2004;57(11):1161-6.

284. Swales M, Hibbs RA, Bryning L, Hastings RP. Health related quality of life for young people receiving dialectical behaviour therapy (DBT): a routine outcome-monitoring pilot. Springerplus 2016;5(1):1137.

285. Szecket N, Medin G, Furlong WJ, Feeny DH, Barr RD, et al. Preliminary translation and cultural adaptation of Health Utilities Index questionnaires for application in Argentina. Int J Cancer. 1999;83(S12):119-124.

286. Tejwani R, Wang HH, Lloyd J, Routh J. Utility estimation for pediatric vesicoureteral reflux: Methodological considerations using an online survey platform. J Urol 2016;195(4Suppl1):e737-8.

287. Teuffel O, Amir E, Alibhai SM, Beyene J, Sung L. Cost-effectiveness of outpatient management for febrile neutropenia in children with cancer. Pediatrics. 2011;127(2):e279-86.288.

288. Thomas KS, Koller K, Dean T, O’Leary CJ, Sach TH, et al. A multicentre randomised controlled trial and economic evaluation of ion-exchange water softeners for the treatment of eczema in children: the Softened Water Eczema Trial (SWET). Health technol assess. 2011;15(8):v-156.

289. Thorrington D, Balasegaram S, Cleary P, Hay C, Eames K. Social and economic impacts of school influenza outbreaks in England: survey of caregivers. J Sch Health 2017;87(3):209-16.

290. Tilford JM, Grosse SD, Robbins JM, Pyne JM, Cleves MA, et al. Health state preference scores of children with spina bifida and their caregivers*.* Qual Life Res. 2005;14(4):1087-98.291.

291. Tilford JM, Aitken ME, Goodman AC, Fiser DH, Killingsworth JB, et al. Child health-related quality of life following neurocritical care for traumatic brain injury: an analysis of preference-weighted outcomes*.* Neurocrit Care. 2007;7(1):64-75.

292. Tilford JM, Payakachat N, Kovacs E, Pyne JM, Brouwer W, et al. Preference-based health-related quality-of-life outcomes in children with autism spectrum disorders: a comparison of generic instruments. Pharmacoeconomics. 2012;30(8):661-79.

293. Tilford JM, Payakachat N, Kuhlthau KA, Pyne JM, Kovacs E, et al. Treatment for sleep problems in children with autism and caregiver spillover effects. J autism dev disord. 2015;45(11):3613-3623.

294. Tirado Y, Chadha NK, Allegro J, Forte V, Campisi P. Quality of life and voice outcomes after thyroid ala graft laryngotracheal reconstruction in young children. Otolaryngol Head Neck Surg. 2011;144(5):770-7.

295. Tong A, Tjaden L, Howard K, Wong G, Morton R, et al. Quality of life of adolescent kidney transplant recipients. J Pediatr. 2011;159(4):670-5 e2.

296. Tong A, Wong G, McTaggart S, Henning P, Mackie F, et al. Quality of life of young adults and adolescents with chronic kidney disease*.* J Pediatr. 2013;163(4):1179-85 e5.

297. Trent M, Lehmann HP, Qian Q, Thompson CB, Ellen JM, et al. Adolescent and parental utilities for the health states associated with pelvic inflammatory disease (PID). J Adolesc Health. 2011;48(2 SUPPL. 1):S69.

298. Trevino RP, Pham TH, Edelstein SL. Obesity and preference-weighted quality of life of ethnically diverse middle school children: the HEALTHY study*.* J Obes, 2013;206074.

299. Trudel JG, Rivard M, Dobkin PL, Leclerc JM, Robaey P. Psychometric properties of the Health Utilities Index Mark 2 system in paediatric oncology patients. Qual Life Res. 1998;7(5):421-32.

300. Ulfsdotter M, Lindberg L, Mansdotter A. A cost-effectiveness analysis of the Swedish universal parenting program All Children in Focus. PLoS ONE 2015;10(12):e0145201.

301. van Baar ME, Polinder S, Essink-Bot ML, van Loey NE, Oen IM, et al. Quality of life after burns in childhood (5-15 years): children experience substantial problems. Burns. 2011;37(6):930-8.

302. van der Kolk A, Bouwmans CA, Schawo SJ, Buitelaar JK, van Agthoven M, et al. Association between quality of life and treatment response in children with attention Deficit Hyperactivity Disorder and their parents. J Ment Health Policy Econ. 2014;17(3):119-29.

303. van Litsenburg RR, Huisman J, Raat H, Kaspers GJL, Gemke RJBJ. Health-related quality of life and utility scores in short-term survivors of pediatric acute lymphoblastic leukemia. Qual Life Res. 2013;22(3):677-81.

304. van Schaik CS, Barr RD, Depauw S, Furlong W, Feeny D. Assessment of health status and health‐related quality of life in survivors of Hodgkin's disease in childhood. Int J Cancer. 1999;83(S12):32-38.

305. van Schooneveld MMJ, Braun KPJ, van Rijen PC, van Nieuwenhuizen O, Jennekens-Schinkel A. The spectrum of long-term cognitive outcome after hemispherectomy in childhood*.* Eur J Paediatr Neurol. 2016;20:376-84.

306. Vermeulen KM, Jansen D, Buskens E, Knorth EJ, Reijneveld SA. Serious child and adolescent behaviour disorders; a valuation study by professionals, youth and parents. BMC Psychiatry 2017;17(1):208.

307. Vermeulen KM, Jansen D, Knorth EJ, Buskens E, Reijneveld SA. Cost-effectiveness of multisystemic therapy versus usual treatment for young people with antisocial problems. Crim Behav Ment Health 2017;27(1):89-102.

308. Verrips GH, Stuifbergen MC, den Ouden AL, Bonsel GJ, Gemke RJ, et al. Measuring health status using the Health Utilities Index: agreement between raters and between modalities of administration. J Clin Epidemiol. 2001;54(5):475-81.309.

309. Verrips E, Vogels T, Saigal S, Wolke D, Meyer R, et al. Health-related quality of life for extremely low birth weight adolescents in Canada, Germany, and the Netherlands. Pediatrics. 2008;122(3):556-61.

310. Verrips G, Brouwer L, Vogels T, Taal E, Drossaert C, et al. Long term follow-up of health-related quality of life in young adults born very preterm or with a very low birth weight. Health Qual Life Outcomes. 2012;10:49.

311. Vitale MG, Levy DE, Johnson MG, Gelijins AC, Moskowitz AJ, et al. Assessment of quality of life in adolescent patients with orthopaedic problems: are adult measures appropriate?. J Pediatr Orthop. 2001;21(5):622-8.

312. Wake M, Levickis P, Tobin S, Gold L, Ukoumunne OC, et al. Two-year outcomes of a population-based intervention for preschool language delay: an RCT. Pediatrics 2015;136(4):e838-47.

313. Wang QN, Furlong W, Feeny D, Torrance G, Barr R. How robust is the Health Utilities Index Mark 2 utility function?. Med Decis Making. 2002;22(4):350-358.

314. Wasserman J, Aday LA, Begley CE, Ahn C, Lairson DR. Measuring health state preferences for hemophilia: development of a disease-specific utility instrument*.* Haemophilia. 2005;11(1):49-57.

315. Weigel A, Konig HH, Gumz A, Lowe B, Brettschneider C. Correlates of health related quality of life in anorexia nervosa. Int J Eat Disord 2016;49(6):630-4.

316. Weiner B, Michelagnoli M, Drake R, Christie D. Screening for distress in young people after treatment for sarcoma: A feasibility study. J Pediatr Oncol Nurs 2016;33(1):25-32.

317. Wijnen BF, de Kinderen RJ, Lambrechts D, Postulart D, Aldenkamp AP, et al. Long-term clinical outcomes and economic evaluation of the ketogenic diet versus care as usual in children and adolescents with intractable epilepsy. Epilepsy Res 2017;132:91-9.

318. Wille N, Badia X, Bonsel G, Burstrom K, Cavrini G, et al. Development of the EQ-5D-Y: a child-friendly version of the EQ-5D. Qual Life Res 2010;19(6):875-86.

319. Willems DC, Joore MA, Hendriks JJ, Wouters EF, Severens JL. Cost-effectiveness of a nurse-led telemonitoring intervention based on peak expiratory flow measurements in asthmatics: results of a randomised controlled trial*.* Cost Eff Resour Alloc. 2007;5(1):1.320.

320. Willems DC, Joore MA, Nieman FH, Severens JL, Wouters EF, et al. Using EQ-5D in children with asthma, rheumatic disorders, diabetes, and speech/language and/or hearing disorders. Int J Technol Assess 2009;25(3):391-9.

321. Wolke D, Chernova J, Eryigit-Madzwamuse S, Samara M, Zwierzynska K, et al. Self and parent perspectives on health-related quality of life of adolescents born very preterm*.* J Pediatr. 2013;163(4):1020-6 e2.

322. Wong CK, Cheung PW, Samartzis D, Luk KD, Cheung KM, et al. Mapping the SRS-22r questionnaire onto the EQ-5D-5L utility score in patients with adolescent idiopathic scoliosis. PLoS One 2017;12(4):e0175847.

323. Wright MJ, Galea V, Barr RD. Self-perceptions of physical activity in survivors of acute lymphoblastic leukemia in childhood. Pediatr Exerc Sci. 2003;15(2):191-201.

324. Wright MJ, Galea V, Barr RD. Proficiency of balance in children and youth who have had acute lymphoblastic leukemia*.* Phys Ther. 2005;85(8):782-90.

325. Wright B, Tindall L, Littlewood E, Allgar V, Abeles P, et al. Computerised cognitive-behavioural therapy for depression in adolescents: feasibility results and 4-month outcomes of a UK randomised controlled trial. BMJ Open 2017;7(1):e012834.

326. Wu XY, Ohinmaa A, Veugelers PJ. Sociodemographic and neighbourhood determinants of health-related quality of life among grade-five students in Canada*.* Qual Life Res. 2010;19(7):969-76.

327. Wu XY, Ohinmaa A, Veugelers PJ. Diet quality, physical activity, body weight and health-related quality of life among grade 5 students in Canada*.* Public Health Nutr. 2012;15(1):75-81.

328. Wu XY, Ohinmaa A, Johnson JA, Veugelers PJ. Assessment of children's own health status using visual analogue scale and descriptive system of the EQ-5D-Y: linkage between two systems*.* Qual Life Res. 2014;23(2):393-402.

329. Xu F, Chen G, Stevens K, Zhou H, Qi S, et al. Measuring and valuing health-related quality of life among children and adolescents in mainland China--a pilot study*.* PLoS One. 2014;9(2):e89222.

330. Yaris N, Yavuz MN, Yavuz AA, Okten A. Assessment of quality of life in pediatric cancer patients at diagnosis and during therapy*.* Turk J Cancer. 2001;31(4):139-149.

331. Yi MS, Britto MT, Wilmott RW, Kotagal UR, Eckman MH, et al. Health values of adolescents with cystic fibrosis*.* J Pediatr. 2003;142(2):133-40.

332. Yi MS, Britto MT, Sherman SN, Moyer MS, Cotton S, et al. Health values in adolescents with or without inflammatory bowel disease*.* J Pediatr. 2009;154(4):527-34.

333. Young NL, Rochon TG, McCormick A, Law M, WedgeJH, et al. The health and quality of life outcomes among youth and young adults with cerebral palsy*.* Arch Phys Med Rehabil. 2010;91(1):143-8.

334. Young NL, Sheridan K, Burke TA, Mukherjee S, McCormick A. Health outcomes among youths and adults with spina bifida*.* J Pediatr. 2013;162(5):993-8.

335. Rodriguez-Martinez CE, Sossa-Briceno MP, Castro-Rodriguez JA. Cost-utility analysis of once-daily versus twice-daily inhaled corticosteroid dosing for maintenance treatment of asthma in pediatric patients. J Asthma 2016;53(5):538-45.

| **Table S4.** Number of studies (% in interval) by study design and 5-year publication interval | | | | | | | |
| --- | --- | --- | --- | --- | --- | --- | --- |
|  | 1990-1994 | 1995-1999 | 2000-2004 | 2005-2009 | 2010-2014 | 2015-2017* | Total |
| Patient case series | 5  (62.5) | 4  (19.0) | 8  (23.5) | 8  (12.7) | 16  (13.3) | 8  (9.0) | 49  (14.6) |
| Cross-sectional survey | 2  (25.0) | 12  (57.1) | 18  (52.9) | 35  (55.6) | 59  (49.2) | 45  (50.6) | 171  (51.0) |
| Longitudinal studies and RCTs without CUA | 1  (12.5) | 2  (9.5) | 4  (11.8) | 7  (11.1) | 20  (16.7) | 13  (14.6) | 47  (14.0) |
| Longitudinal studies and RCTs with CUA and decision models with primary utility assessment | 0  (0.0) | 3  (14.4) | 4  (11.8) | 13  (20.6) | 25  (20.8) | 23  (25.8) | 68  (20.4) |
|  | 8  (2.4) | 21  (6.3) | 34  (10.1) | 63  (18.8) | 120  (35.8) | 89  (26.6) | 335 |
| * Covers up to 30^th^ June 2017; RCT: Randomised controlled trial; CUA: Cost-utility analysis | | | | | | | |

| **Table S5.** Number of samples (% in interval) by ICD-10 chapter and 5-year publication interval | | | | | | | |
| --- | --- | --- | --- | --- | --- | --- | --- |
|  | 1990-1994 | 1995-1999 | 2000-2004 | 2005-2009 | 2010-2014 | 2015-2017* | Total |
| General health | 1  (1.0) | 9  (4.3) | 15  (3.4) | 23  (2.6) | 316  (20.1) | 137  (17.8) | 501  (12.6) |
| ICD-10 Chapter 1: Infectious and parasitic diseases | 36  (36.0) |  | 49  (11.2) | 64  (7.3) | 39  (2.5) | 7  (0.9) | 195  (4.9) |
| ICD-10 Chapter 2: Cancer | 33  (33.0) | 108  (50.9) | 105  (24.0) | 89  (10.1) | 93  (5.9) | 14  (1.8) | 442  (11.1) |
| ICD-10 Chapter 3: Diseases of the blood and immune system |  |  | 31  (7.1) | 20  (2.3) | 22  (1.4) | 12  (1.6) | 85  (2.1) |
| ICD-10 Chapter 4: Endocrine, nutritional and metabolic disorders | 3  (3.0) | 6  (2.8) | 39  (8.9) | 43  (4.9) | 258  (16.4) | 62  (8.0) | 411  (10.3) |
| ICD-10 Chapter 5: Mental and behavioural disorders | 3  (3.0) |  |  | 166  (18.9) | 305  (19.4) | 224  (29.0) | 698  (17.6) |
| ICD-10 Chapter 6: Nervous system disorders | 3  (3.0) | 3  (1.4) | 1  (0.2) | 33  (3.8) | 38  (2.4) | 36  (4.7) | 114  (2.9) |
| ICD-10 Chapter 7: Diseases of the eye |  | 1  (0.5) | 44  (10.1) | 16  (1.8) |  |  | 61  (1.5) |
| ICD-10 Chapter 8: Diseases of the ear | 6  (6.0) | 6  (2.8) | 6  (1.4) | 64  (7.3) | 74  (4.7) | 2  (0.3) | 158  (4.0) |
| ICD-10 Chapter 9: Circulatory system disorders | 3  (3.0) | 3  (1.4) | 4  (0.9) | 5  (0.6) | 1  (0.1) |  | 16  (0.4) |
| ICD-10 Chapter 10: Respiratory system disorders | 3  (3.0) | 6  (2.8) | 4  (0.9) | 36  (4.1) | 123  (7.8) | 20  (2.6) | 192  (4.8) |
| ICD-10 Chapter 11: Digestive system disorders |  | 1  (0.5) | 4  (0.9) | 6  (0.7) | 25  (1.6) | 24  (3.1) | 60  (1.5) |
| ICD-10 Chapter 12: Diseases of the skin |  | 1  (0.5) | 9  (2.1) | 11  (1.3) | 5  (0.3) |  | 26  (0.7) |
| ICD-10 Chapter 13: Musculoskeletal system disorders |  | 2  (0.9) | 34  (7.8) | 8  (0.9) | 17  (1.1) | 53  (6.9) | 114  (2.9) |
| ICD-10 Chapter 14: Genitourinary system disorders | 3  (3.0) | 1  (0.5) | 3  (0.7) | 4  (0.5) | 44  (2.8) | 1  (0.1) | 56  (1.4) |
| ICD-10 Chapter 16: Conditions originating in the perinatal period | 6  (6.0) | 47  (22.2) | 54  (12.3) | 30  (3.4) | 64  (4.1) | 9  (1.2) | 210  (5.3) |
| ICD-10 Chapter 17: Congenital malformations |  | 4  (1.9) | 8  (1.8) | 26  (3.0) | 29  (1.8) | 88  (11.4) | 155  (3.9) |
| ICD-10 Chapter 19: Injury, poisoning and other consequences of external causes |  | 3  (1.4) | 4  (0.9) | 211  (24.0) | 65  (4.1) | 14  (1.8) | 297  (7.5) |
| ICD-10 Chapter 21: Contact with health services |  | 10  (4.7) |  | 3  (0.3) | 35  (2.2) | 32  (4.2) | 80  (2.0) |
| Combined chronic diseases |  | 1  (0.5) | 24  (5.5) | 20  (2.3) | 21  (1.3) | 37  (4.8) | 103  (2.6) |
| Total | 100  (2.5) | 212  (5.3) | 438  (11.0) | 878  (22.1) | 1,574  (39.6) | 772  (19.4) | 3,974 |
| * Covers up to 30^th^ June 2017 | | | | | | | |

| **Table S6.** Number of samples (% in interval) by valuation method and 5-year publication interval | | | | | | | |
| --- | --- | --- | --- | --- | --- | --- | --- |
|  | 1990-1994 | 1995-1999 | 2000-2004 | 2005-2009 | 2010-2014 | 2015-2017* | Total |
| Visual analogue scales | 57  (57.0) | 22  (10.4) | 53  (12.1) | 104  (11.9) | 326  (20.7) | 270  (35.0) | 832  (20.9) |
| Trade-off-based direct valuation methods |  | 37  (17.5) | 184  (42.0) | 178  (20.3) | 102  (6.5) | 40  (5.2) | 541  (13.6) |
| Adult-specific MAUIs |  |  | 20  (4.6) | 116  (13.2) | 210  (13.3) | 130  (16.8) | 476  (12.0) |
| MAUIs compatible with childhood and adult populations | 43  (43.0) | 139  (65.6) | 178  (40.6) | 416  (47.4) | 613  (39.0) | 148  (19.2) | 1,537  (38.7) |
| Children- and/or adolescent-specific MAUIs |  | 14  (6.6) | 3  (0.7) | 57  (6.5) | 323  (20.5) | 176  (22.8) | 573  (14.4) |
| Utility from non-preference-based methods |  |  |  | 7  (0.8) |  | 8  (1.0) | 15  (0.4) |
| Total | 100  (2.5) | 212  (5.3) | 438  (11.0) | 878  (22.1) | 1,574  (39.6) | 772  (19.4) | 3,974 |
| * Covers up to 30^th^ June 2017 | | | | | | | |

| **Table S7.** Number of samples (% in interval) by (i) age of sample, (ii) respondent type, (iii) administration mode and (iv) valuation of hypothetical state and 5-year publication category | | | | | | | |
| --- | --- | --- | --- | --- | --- | --- | --- |
|  | 1990-1994 | 1995-1999 | 2000-2004 | 2005-2009 | 2010-2014 | 2015-2017* | Total |
| **(i) Age of sample**** | | | | | | | |
| Sample contains pre-adolescent children | 55  (100) | 78  (44.6) | 161  (52.3) | 484  (64.2) | 931  (66.3) | 377  (53.6) | 2,086  (61.3) |
| Sample does not contain pre-adolescent children |  | 97  (55.4) | 147  (47.7) | 270  (35.8) | 474  (33.7) | 327  (46.4) | 1,315  (38.7) |
| Total | 55  (1.6) | 175  (5.1) | 308  (9.1) | 754  (22.2) | 1,405  (41.3) | 704  (20.7) | 3,401 |
| **(ii) Respondent type ^a^** | | | | | | | |
| Self-assessment by children | 2  (2.0) | 73  (34.4) | 127  (29.0) | 135  (15.4) | 778  (49.4) | 383  (49.6) | 1,498  (37.7) |
| Proxy assessment | 98  (98.0) | 139  (65.6) | 311  (71.0) | 743  (84.6) | 796  (50.6) | 389  (50.4) | 2,476  (62.3) |
| Total | 100  (2.5) | 212  (5.3) | 438  (11.0) | 878  (22.1) | 1,574  (39.6) | 772  (19.4) | 3,974 |
| **(iii) Administration mode** | | | | | | | |
| Self-administered surveys | 39  (39.0) | 74  (34.9) | 155  (35.4) | 478  (54.4) | 1,079  (68.6) | 639  (82.8) | 2,464  (62.0) |
| Interview-administered surveys | 61  (61.0) | 138  (65.1) | 283  (64.6) | 400  (45.6) | 495  (31.4) | 133  (17.2) | 1,510  (38.0) |
| Total | 100  (2.5) | 212  (5.3) | 438  (11.0) | 878  (22.1) | 1,574  (39.6) | 772  (19.4) | 3,974 |
| **(iv) Valuation of hypothetical state** | | | | | | | |
| Actual health state | 43  (43.0) | 163  (76.9) | 304  (69.4) | 646  (73.6) | 1,467  (93.2) | 624  (80.8) | 3,247  (81.7) |
| Hypothetical health state | 57  (57.0) | 49  (23.1) | 134  (30.6) | 232  (26.4) | 107  (6.8) | 148  (19.2) | 727  (18.3) |
| Total | 100  (2.5) | 212  (5.3) | 438  (11.0) | 878  (22.1) | 1,574  (39.6) | 772  (19.4) | 3,974 |
| * Covers up to 30^th^ June 2017; ** Divided into two categories: samples with mean or median age below 12 or minimum age (if mean/median age not reported) below 12 and samples with mean or median age or minimum age 12 or above.  ^a^ Assessment together by children and parents or caregivers is treated as proxy assessment. | | | | | | | |
